# Design, Synthesis, and Biological Evaluation of Novel Morpholine–Coumarin Derivatives for Inflammation-Associated Depression

**DOI:** 10.3390/biom16071002

**Published:** 2026-07-09

**Authors:** Hui Liu, Lina Hu, Yalan Wang, Zheshan Quan, Zheng Liu, Shiben Wang, Qingkun Shen

**Affiliations:** 1 Key Laboratory of Natural Medicines of the Changbai Mountain, Yanbian University, Ministry of Education, Yanji 133002, China; 2023001120@ybu.edu.cn (H.L.); 2019010636@ybu.edu.cn (Y.W.); zsquan@ybu.edu.cn (Z.Q.); 2 State Key Laboratory of Macromolecular Drugs and Large-Scale Preparation, School of Pharmaceutical Sciences and Food Engineering, Liaocheng University, Liaocheng 252059, China; 2019207801@stu.lcu.edu.cn; 3 School of Medicine, Foshan University, Foshan 528000, China; liuzheng8709@fosu.edu.cn

**Keywords:** morpholine–coumarin derivatives, dual IDO1/TDO inhibitor, tryptophan–kynurenine, antidepressant activity, neuroinflammation

## Abstract

The tryptophan–kynurenine pathway, mediated by indoleamine 2,3-dioxygenase 1 (IDO1) and tryptophan 2,3-dioxygenase (TDO), is critically involved in the pathogenesis of depression. A series of novel morpholine–coumarin derivatives were designed and synthesized as dual IDO1/TDO inhibitors. Through in vitro enzyme screening, compound **14d** exhibited potent inhibitory activity with IC_50_ values of 0.34 µM and 0.75 µM, respectively. In lipopolysaccharide (LPS)-stimulated BV2 microglial cells, **14d** downregulated IDO1/TDO expression, suppressed pro-inflammatory cytokines (IL-1β, COX-2, iNOS, TNF-α), and upregulated the anti-inflammatory cytokine IL-10. In an LPS-induced acute depressive mouse model established in C57BL/6 mice, intraperitoneal administration of **14d** (20 mg/kg) significantly reduced immobility time in the forced swim and tail suspension tests, without affecting spontaneous locomotor activity. Mechanistic studies revealed that **14d** inhibited microglial activation in the hippocampal dentate gyrus, reduced cerebral kynurenine levels, increased serotonin content, and upregulated BDNF/PKA signaling. Molecular docking further predicted the binding interactions of **14d** with the active sites of IDO1 and TDO. These findings suggest that **14d** represents a promising dual IDO1/TDO inhibitor lead compound for the treatment of inflammation-associated depression through modulation of the kynurenine pathway and neuroinflammatory responses.

## 1. Introduction

Major depressive disorder (MDD) is a common mental health disorder that affects millions of individuals, causing significant disability, distress, impairment of daily functioning, and reduced quality of life [1,2]. Its core symptoms include persistent low mood and anhedonia, often accompanied by cognitive decline and neurological dysfunction, and in severe cases, it may even lead to suicide [3]. The pathogenesis involves genetic susceptibility, neurotransmitter imbalances (e.g., norepinephrine, dopamine, serotonin), neuroinflammation, and hippocampal neuronal damage [4,5]. About 30% of patients are resistant to conventional antidepressants; first-line selective serotonin reuptake inhibitors (SSRIs, e.g., fluoxetine) have only 33–50% remission rates and cause side effects like sexual dysfunction and discontinuation syndrome [6]. Thus, developing highly effective, low-toxicity antidepressants that modulate neurotransmitters, reduce inflammation, and protect neurons is a key research direction.

**Figure 1:**
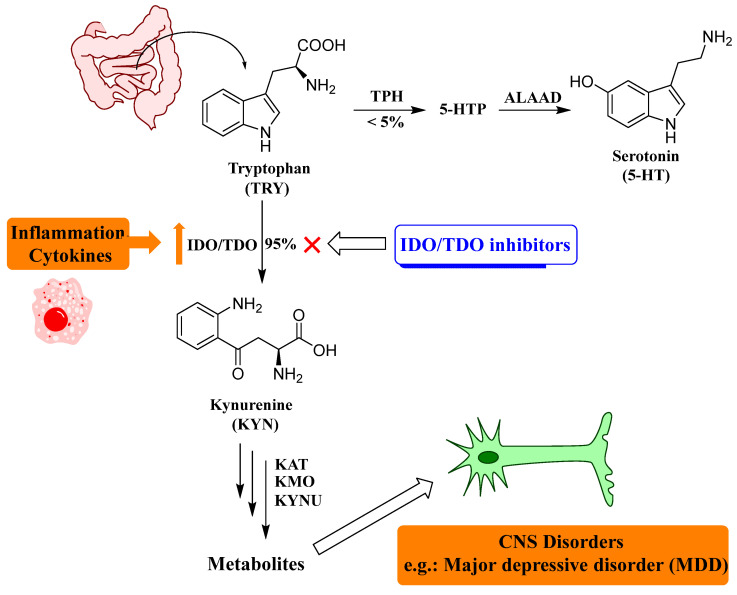
Tryptophan–kynurenine pathway.

Tryptophan (TRY), an essential amino acid in humans, is metabolized approximately 95% via the kynurenine (KYN) pathway (Figure 1). The initial step of this pathway is catalyzed by two rate-limiting enzymes: indoleamine 2,3-dioxygenase (IDO) and tryptophan 2,3-dioxygenase (TDO), which convert TRY to *N*-formylkynurenine, subsequently yielding KYN [7,8]. Currently, many inhibitors targeting IDO1 (a subtype), TDO, or the IDO1/TDO dual inhibition have been reported. To date, most of these studies have focused on disease areas such as cancer and Parkinson’s disease [9,10,11,12,13,14,15,16]; in contrast, research on IDO1/TDO dual inhibitors in the context of depression remains relatively limited [17,18].

Dysregulation of KYN metabolism plays a critical role in the pathogenesis of depression and has become an important target for antidepressant development. First, under inflammatory conditions, IDO1 is overactivated, diverting TRY metabolism toward KYN, leading to TRY depletion in the brain and consequently impairing serotonin (5-HT) synthesis, which results in mood disorders [19]. Clinical studies have shown that serum TRY levels in depressed patients are significantly lower than those in healthy individuals, whereas the KYN/TRY ratio is significantly elevated, indicating enhanced IDO1 activity [20]. Second, studies have demonstrated that various antidepressants can specifically bind to TDO, suggesting that TDO may be a key target for antidepressant action [21]. In summary, KYN dysregulation contributes to the pathogenesis of depression by disrupting neurotransmitter synthesis and promoting excitotoxicity. Modulating the activity of key KYN enzymes (IDO1 and TDO) and the balance of their metabolites represents an important strategy for antidepressant therapy. Future research should further explore the dynamic changes in KYN metabolism and develop more selective drugs targeting this pathway.

**Figure 2:**
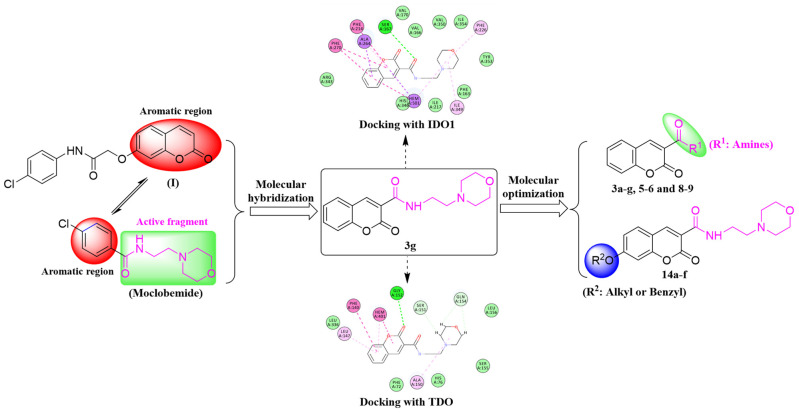
Design of compounds.

Coumarin serves as the core scaffold of numerous natural coumarin compounds. This structure is readily amenable to modification, and by linking various functional groups, it enables the construction of drug candidate molecules with broad biological activities, including anticancer, antibacterial, antiviral, antitubercular, and antidepressant effects [22,23,24,25,26]. Our research group previously designed and synthesized a series of novel coumarin derivatives (**I**) with antidepressant activity. Mechanistic studies revealed that the active compounds elevated cerebral 5-HT levels in experimental animals [27], suggesting that they enhance the serotonergic pathway by inhibiting KYN metabolism. Specifically, these compounds inhibited IDO1 and TDO with half maximal inhibitory concentration (IC_50_) values of 12.34 and 9.52 μM, respectively (Figure 2). Moclobemide is a clinical antidepressant in which the 2-morpholinoethan-1-amine moiety plays an important role in its activity. Studies have shown that moclobemide exhibits superior efficacy compared to some other antidepressants in the treatment of acute-phase depression, with fewer anticholinergic side effects [28].

Based on the above findings, while retaining the coumarin core structure of lead compound **I**, we hybridized it with moclobemide to design and synthesize compound **3g**. Pharmacological results indicated that compound **3g** exhibited better inhibitory activity against both IDO1 and TDO enzymes, with IC_50_ values of 4.09 and 6.20 μM, respectively. Compound **3g**, which showed superior activity, was selected for molecular docking studies with both enzymes. The results revealed that the coumarin structure primarily engaged in hydrophobic interactions (e.g., Pi-Pi stacking) around the enzyme’s active pocket, whereas the amide and morpholine moieties mainly formed hydrogen bonds. Accordingly, it was inferred that the coumarin core and the 2-morpholinoethan-1-amine moiety derived from the moclobemide structure contribute significantly to the activity.

Based on these findings, we adopted two strategies for further structural modification of the compounds. The first strategy involved replacing the 2-morpholinoethan-1-amine moiety (the hydrogen-bonding region) of moclobemide with various heterocyclic rings to investigate their impact on activity. The second strategy, aimed at obtaining compounds with improved activity, was based on the binding modes of compound **3g** with IDO1 and TDO derived from molecular docking. We kept the core structure of **3g** unchanged and introduced different hydrophobic fragments (R^2^) into the amenable hydrophobic region, hoping to generate additional interactions and thereby enhance inhibitory potency. Consequently, a series of target compounds were designed and synthesized with the goal of obtaining antidepressant candidates possessing stronger dual IDO1/TDO inhibitory activity (Figure 2).

## 2. Materials and Methods

### 2.1. Chemistry

Instruments and test conditions: Melting points were determined on an X-4 micro melting point apparatus (Shanghai Precision Scientific Instrument Co., Ltd., Shanghai, China) and were uncorrected. ^1^H-NMR and ^13^C-NMR spectra were acquired on a Bruker AV-500 nuclear magnetic resonance spectrometer (Bruker, Fällanden, Switzerland) using DMSO-*d*_6_ or CDCl_3_ as the solvent and tetramethylsilane (TMS) as the internal standard. Chemical shifts (δ) are reported in parts per million (ppm), and coupling constants (J) are reported in hertz (Hz). The following abbreviations are used to describe signal splitting patterns: singlet (s), doublet (d), doublet of doublets (dd), triplet (t), quartet (q), and multiplet (m). High-resolution mass spectrometry (HRMS) data were measured on a high-resolution mass spectrometer (Bruker Daltonics, Bremen, Germany).

Reagents and materials: All chemical reagents and solvents used in this study were of analytical grade and were purchased from commercial suppliers, including Energy Chemical (Shanghai, China), Aladdin (Shanghai, China), and Sinopharm Chemical Reagent Co., Ltd. (Shanghai, China), and were used without further purification. Thin-layer chromatography (TLC) plates and column chromatography silica gel (200–300 mesh) were purchased from Qingdao Haiyang Chemical Co., Ltd. (Qingdao, China).

#### 2.1.1. Synthesis of 2-Oxo-2H-chromene-3-carbonyl Chloride (**2**)

A catalytic amount of DMF (0.1 mL) was added to a solution of compound 1 (1.0 mmol) in CH_2_Cl_2_ (30 mL), followed by the dropwise addition of SOCl_2_ (1 mL). The reaction mixture was heated and stirred in a constant-temperature oil bath at 50 °C for 3 h. The reaction progress was monitored by TLC. After complete consumption of the starting material, all solvents were removed under reduced pressure using a rotary evaporator. The resulting crude product **2** was used directly in the next step without further purification.

#### 2.1.2. Synthesis of 3-(Substituted-1-carbonyl)-2H-chromen-2-one (**3a**–**g**, **4** and **7**)

The corresponding amines (1.2 mmol) were added to a solution of compound **2** (1.0 mmol) in CH_2_Cl_2_ (30 mL). The mixture was cooled to 0 °C in an ice-water bath. Triethylamine (3.0 mmol) was added slowly dropwise with magnetic stirring. After the addition, the ice bath was removed, and the reaction mixture was allowed to warm naturally to room temperature and stirred for an additional 2 h. The reaction progress was monitored by TLC until the starting material was consumed. After completion, the reaction mixture was concentrated under reduced pressure using a rotary evaporator to afford a solid residue. Water (15 mL) was added to the residue, and a solid precipitated. The solid was collected by suction filtration, and the filter cake was washed several times with water and dried to give the crude products **3a**–**g**, **4** and **7**. The crude products were purified by silica gel column chromatography using a gradient elution with CH_2_Cl_2_-CH_3_OH (80:1, *v*/*v*). Fractions containing the desired product were combined, and the solvent was removed by rotary evaporation to afford the pure target compounds **3a**–**g**, **4** and **7**.

#### 2.1.3. Synthesis of 2-Oxo-*N*-(2-(piperazin-1-yl)ethyl)-2H-chromene-3-carboxamide Compounds (**5**) and 3-(Piperazine-1-carbonyl)-2H-chromen-2-one (**8**)

CF_3_COOH (5 mL) was added dropwise to a solution of compound **4** (or **7**, 1.0 mmol) in CH_2_Cl_2_ (20 mL). The mixture was stirred at room temperature for 1 h. The reaction progress was monitored by TLC until the starting material was consumed. After completion, the reaction mixture was transferred to a water bath and the solvent was removed under reduced pressure using a vacuum pump to afford a solid residue. Saturated sodium bicarbonate solution (5 mL) was added to the residue, followed by extraction with ethyl acetate (15 mL × 3). The combined organic layers were dried over anhydrous Na_2_SO_4_. The mixture was filtered, and the filtrate was concentrated under reduced pressure using a vacuum pump to give crude compounds **5** or **8**. The crude products were used directly in the next step without further purification.

#### 2.1.4. Synthesis of *N*-(2-(4-Benzylpiperazin-1-yl)ethyl)-2-oxo-2H-chromene-3-carboxamide (**6**) and 3-(4-Benzylpiperazine-1-carbonyl)-2H-chromen-2-one (**9**)

Benzyl bromide (1.2 mmol) and triethylamine (3.0 mmol) were added to a solution of compound **5** (or **8**, 1.0 mmol) in CH_3_CN (20 mL). The mixture was stirred at room temperature for 3 h. The reaction progress was monitored by TLC until the starting material was consumed. After completion, the reaction mixture was transferred to a water bath and the solvent was removed under reduced pressure using a rotary evaporator to afford a solid residue. Water (10 mL) was added to the residue, followed by extraction with ethyl acetate (10 mL × 3). The combined organic layers were dried over anhydrous Na_2_SO_4_. The mixture was filtered, and the filtrate was concentrated under reduced pressure using a vacuum pump to give crude compounds **6** or **9**. The crude products were purified by silica gel column chromatography using a gradient elution with CH_2_Cl_2_-CH_3_OH (60:1, *v*/*v*). Fractions containing the desired product were combined, and the solvent was removed by rotary evaporation to afford the pure target compounds **6** and **9**.

#### 2.1.5. Synthesis of Ethyl 7-Substituted-2-oxo-2H-chromene-3-carboxylate (**11a**–**j**)

The corresponding substituted bromide (R^2^Br, 1.2 mmol), K_2_CO_3_ (1.2 mmol), and a catalytic amount of KI (ca. 10 mol%) were added to a solution of compound **10** (1.0 mmol) in acetone (20 mL). The mixture was stirred at 55 °C in an oil bath for 4 h. The reaction progress was monitored by TLC until the starting material was consumed. After completion, the reaction mixture was transferred to a water bath and the solvent was removed under reduced pressure using a vacuum pump to afford a solid residue. Water (15 mL) was added to the residue, and a solid precipitated. The solid was collected by suction filtration, and the filter cake was washed several times with water and dried to give the crude products **11a**–**j**. The crude products were purified by silica gel column chromatography using a gradient elution with CH_2_Cl_2_-CH_3_OH (150:1, *v*/*v*). Fractions containing the desired product were combined, and the solvent was removed by rotary evaporation to afford the pure target compounds **11a**–**j**.

#### 2.1.6. Synthesis of Compound 7-Substituted-2-oxo-2H-chromene-3-carboxylic Acid (**12a**–**j**)

The corresponding compound **11a**–**j** (1.0 mmol) was added to a solution of NaOH (5.0 mmol) in a mixed solvent (H_2_O-CH_3_OH, 1:5, *v*/*v*, total volume 15 mL). The mixture was stirred at 55 °C in an oil bath for 2 h. The reaction progress was monitored by TLC until the starting material was consumed. The reaction mixture was transferred to a water bath and the solvent was removed under reduced pressure using a rotary evaporator to afford a solid residue. Water (15 mL) was added to the residue, and the pH was adjusted to 2–4 with 1 mol/L dilute hydrochloric acid under stirring, whereupon a solid precipitated. The solid was collected by suction filtration, and the filter cake was washed several times with water and dried to give the crude products **12a**–**j**. The crude products were used directly in the next step without further purification.

#### 2.1.7. Synthesis of 7-Substituted-2-oxo-2H-chromene-3-carbonyl Chloride (**13a**–**j**)

A catalytic amount of DMF (0.1 mL) was added to a solution of compound **12a**–**j** (1.0 mmol) in CH_2_Cl_2_ (30 mL), followed by the dropwise addition of SOCl_2_ (1 mL). The reaction mixture was heated and stirred in a constant-temperature oil bath at 50 °C for 3 h. The reaction progress was monitored by TLC. After complete consumption of the starting material, all solvents were removed under reduced pressure using a rotary evaporator. The resulting crude products **13a**–**j** were used directly in the next step without further purification.

#### 2.1.8. Synthesis of 7-Substituted-*N*-(2-morpholinoethyl)-2-oxo-2H-chromene-3-carboxamide (**14a**–**j**)

2-morpholinoethan-1-amine (1.2 mmol) was added to a solution of compound **13a**–**j** (1.0 mmol) in CH_2_Cl_2_ (30 mL). The mixture was cooled to 0 °C in an ice-water bath. Triethylamine (3.0 mmol) was added slowly dropwise with magnetic stirring. After the addition, the ice bath was removed, and the reaction mixture was allowed to warm naturally to room temperature and stirred for an additional 2 h. The reaction progress was monitored by TLC. After the disappearance of the starting material spot, the reaction mixture was concentrated under reduced pressure using a rotary evaporator to afford a solid product. Water (15 mL) was then added, and the solid was collected by suction filtration and washed with water to give the crude product. The crude product was purified by silica gel column chromatography using a gradient elution with CH_2_Cl_2_-CH_3_OH (100:1, *v*/*v*). Fractions containing the desired product were combined, and the solvent was removed by rotary evaporation to afford the pure target compounds **14a**–**j**.

The analytical results of the ^1^H-NMR, ^13^C-NMR, and HRMS data for all target compounds are as follows (See the Supplementary Information for all spectra, Figures S5–S67).

*N*-butyl-2-oxo-2H-chromene-3-carboxamide (**3a**)

White solid, 55 mg. Yield: 39%. Mp: 90–92 °C. ^1^H-NMR (CDCl_3_, 500 MHz, ppm) *δ*: 8.92 (s, 1H, Coumarin-4-H), 8.80 (s, 1H, CONH), 7.35–7.71 (m, 4H, Ar-H), 3.47 (q, 2H, *J* = 6.7 Hz, NHCH_2_), 1.41–1.66 (m, 4H, 2CH_2_), 0.97 (t, 3H, *J* = 7.5 Hz, CH_3_). ^13^C-NMR (126 MHz, CDCl_3_) *δ*: 161.50, 161.40, 154.42, 148.17, 133.92, 129.78, 125.25, 118.72, 118.65, 116.61, 39.65, 31.46, 20.21, 13.76. ESI-HRMS calcd for C_14_H_15_NNaO_3_^+^ ([M+Na]^+^): 268.0944; found: 268.0954. The ^1^H-NMR, ^13^C-NMR, and HRMS spectra are presented in Supplementary Figures S5–S7.

*N*-(4-fluorophenyl)-2-oxo-2H-chromene-3-carboxamide (**3b**)

White solid, 63 mg. Yield: 46%. Mp: 245–246 °C. ^1^H-NMR (CDCl_3_, 500 MHz, ppm) *δ*: 10.82 (s, 1H, CONH), 9.02 (s, 1H, Coumarin-4-H), 7.06–7.75 (m, 8H, Ar-H). ^13^C-NMR (126 MHz, CDCl_3_) *δ*: 161.85, 159.68 (d, *J* = 244.3 Hz), 159.31, 158.71, 154.52, 149.01, 134.45, 133.74 (d, *J* = 2.9 Hz), 129.97, 125.54, 122.27 (d, *J* = 7.9 Hz), 118.70, 118.51, 116.77, 115.74 (d, *J* = 22.5 Hz). ESI-HRMS calcd for C_16_H_10_FNNaO_3_^+^ ([M+Na]^+^): 306.0537; found: 306.0542. The ^1^H-NMR, ^13^C-NMR, and HRMS spectra are presented in Supplementary Figures S8–S10.

3-(Morpholine-4-carbonyl)-2H-chromen-2-one (**3c**)

White solid, 80 mg. Yield: 37%. Mp: 55–56 °C. ^1^H-NMR (CDCl_3_, 500 MHz, ppm) *δ*: 7.96 (s, 1H, Coumarin-4-H), 7.32–7.62 (m, 4H, Ar-H), 3.40–3.79 (m, 8H, Morpholine-H). ^13^C NMR (126 MHz, CDCl_3_) *δ*: 163.56, 157.94, 154.19, 143.70, 133.01, 128.62, 124.98, 124.82, 118.27, 116.84, 66.70, 66.62, 47.64, 42.63. ESI-HRMS calcd for C_14_H_13_NNaO_4_^+^ ([M+Na]^+^): 282.0737; found: 282.0741. The ^1^H-NMR, ^13^C-NMR, and HRMS spectra are presented in Supplementary Figures S11–S13.

*N*-cyclopentyl-2-oxo-2H-chromene-3-carboxamide (**3d**)

White solid, 71 mg. Yield: 45%. Mp: 126–127 °C. ^1^H-NMR (CDCl_3_, 500 MHz, ppm) *δ*: 8.91 (s, 1H, Coumarin-4-H), 8.80–8.81 (d, 1H, *J* = 5.0 Hz, CONH), 7.36–7.70 (m, 4H, Ar-H), 1.55–4.41 (m, 9H, Cyclopentylamine-H). ^13^C-NMR (126 MHz, CDCl_3_) *δ*: 161.47, 160.87, 154.40, 148.03, 133.85, 129.74, 125.22, 118.73, 118.71, 116.59, 51.57, 33.09, 23.83. ESI-HRMS calcd for C_15_H_15_NNaO_3_^+^ ([M+Na]^+^): 280.0944; found: 280.0951. The ^1^H-NMR, ^13^C-NMR, and HRMS spectra are presented in Supplementary Figures S14–S16.

3-(Pyrrolidine-1-carbonyl)-2H-chromen-2-one (**3e**)

White solid, 60 mg. Yield: 47%. Mp: 129–130 °C. ^1^H-NMR (CDCl_3_, 500 MHz, ppm) *δ*: 7.97 (s, 1H, Coumarin-4-H), 7.31–7.61 (m, 4H, Ar-H), 3.45–3.66 (2t, 4H, *J* = 7.5 Hz, *J* = 7.5 Hz, CH_2_NCH_2_), 1.92–1.99 (m, 4H, 2NCH_2_CH_2_). ^13^C-NMR (126 MHz, CDCl_3_) *δ*: 162.25, 156.73, 153.16, 142.11, 131.77, 127.63, 125.29, 123.85, 117.35, 115.75, 46.55, 45.24, 25.00, 23.30. ESI-HRMS calcd for C_14_H_13_NNaO_3_^+^ ([M+Na]^+^): 266.0788; found: 266.0798. The ^1^H-NMR, ^13^C-NMR, and HRMS spectra are presented in Supplementary Figures S17–S19.

2-Oxo-*N*-(2-(pyrrolidin-1-yl)ethyl)-2H-chromene-3-carboxamide (**3f**)

White solid, 55 mg. Yield: 42%. Mp: 192–193 °C. ^1^H-NMR (CDCl_3_, 500 MHz, ppm) *δ*: 9.16 (t, 1H, *J* = 5.0 Hz, CONH), 8.85 (s, 1H, Coumarin-4-H), 7.39–7.71 (m, 4H, Ar-H), 4.02 (q, 2H, *J* = 6.7 Hz, NHCH_2_), 3.38 (q, 2H, *J* = 6.7 Hz, NHCH_2_CH_2_), 2.08–3.93 (m, 8H, pyrrolidine-H). ^13^C-NMR (126 MHz, CDCl_3_) *δ*: 162.73, 161.11, 154.56, 148.65, 134.46, 129.94, 125.41, 118.45, 117.96, 116.77, 54.11, 53.76, 36.17, 23.46. ESI-HRMS calcd for C_16_H_19_N_2_O_3_^+^ ([M+H]^+^): 287.1390; found: 287.1397. The ^1^H-NMR, ^13^C-NMR, and HRMS spectra are presented in Supplementary Figures S20–S22.

*N*-(2-morpholinoethyl)-2-oxo-2H-chromene-3-carboxamide (**3g**)

White solid, 68 mg. Yield: 52%. Mp: 128–129 °C. ^1^H-NMR (CDCl_3_, 500 MHz, ppm) *δ*: 9.16 (s, 1H, CONH), 8.90 (s, 1H, Coumarin-4-H), 7.37–7.71 (m, 4H, Ar-H), 3.60 (q, 2H, *J* = 6.7 Hz, NHCH_2_), 2.63 (t, 2H, *J* = 5.0 Hz, NHCH_2_CH_2_), 2.55–3.78 (m, 8H, Morpholine-H). ^13^C-NMR (126 MHz, CDCl_3_) *δ*: 161.28, 154.46, 148.14, 133.99, 129.78, 125.25, 118.65, 118.62, 116.63, 66.98, 56.77, 53.39, 36.59. ESI-HRMS calcd for C_16_H_19_N_2_O_4_^+^ ([M+H]^+^): 303.1339; found: 303.1347. The ^1^H-NMR, ^13^C-NMR, and HRMS spectra are presented in Supplementary Figures S23–S25.

2-Oxo-*N*-(2-(piperazin-1-yl)ethyl)-2H-chromene-3-carboxamide (**5**)

White solid, 63 mg. Yield: 36%. Mp: 166–167 °C. ^1^H-NMR (DMSO-*d*_6_, 500 MHz, ppm) *δ*: 9.33 (s, 1H, NH), 8.96 (t, 1H, *J* = 7.5 Hz, CONH), 8.91 (s, 1H, Coumarin-4-H), 7.45–8.02 (m, 4H, Ar-H), 3.69 (q, 2H, *J* = 6.7 Hz, NHCH_2_), 3.27–3.39 (m, 10H, Piperazine-H, NHCH_2_CH_2_). ^13^C-NMR (126 MHz, DMSO-*d*_6_) *δ*: 162.35, 160.62, 159.22, 154.41, 148.34, 134.76, 130.82, 125.66, 118.84, 116.60, 55.61, 48.93, 41.02, 34.91. ESI-HRMS calcd for C_16_H_20_N_3_O_3_^+^ ([M+H]^+^): 302.1499; found: 302.1508. The ^1^H-NMR, ^13^C-NMR, and HRMS spectra are presented in Supplementary Figures S26–S28.

*N*-(2-(4-benzylpiperazin-1-yl)ethyl)-2-oxo-2H-chromene-3-carboxamide (**6**)

White solid, 79 mg. Yield: 48%. Mp: 126–127 °C. ^1^H-NMR (CDCl_3_, 500 MHz, ppm) *δ*: 9.13 (s, 1H, CONH), 8.90 (s, 1H, Coumarin-4-H), 7.31–7.71 (m, 9H, Ar-H), 3.74 (s, 2H, CH_2_), 3.59 (q, 2H, *J* = 6.7 Hz, NHCH_2_), 2.70–2.76 (m, 10H, Piperazine-H, NHCH_2_CH_2_). ^13^C-NMR (126 MHz, CDCl_3_) *δ*: 161.50, 161.33, 154.44, 148.24, 134.05, 129.96, 129.82, 128.62, 125.30, 118.63, 118.54, 116.62, 62.26, 55.91, 52.49, 51.67, 36.66, 29.70. ESI-HRMS calcd for C_23_H_26_N_3_O_3_^+^ ([M+H]^+^): 392.1969; found: 392.1976. The ^1^H-NMR, ^13^C-NMR, and HRMS spectra are presented in Supplementary Figures S29–S31.

3-(Piperazine-1-carbonyl)-2H-chromen-2-one (**8**)

White solid, 76 mg. Yield: 60%. Mp: 205–206 °C. ^1^H-NMR (DMSO-*d*_6_, 500 MHz, ppm) *δ*: 9.19 (s, 1H, NH), 8.92 (s, 1H, Coumarin-4-H), 7.45–8.03 (m, 4H, Ar-H), 3.23–3.67 (m, 8H, Piperazine-H). ^13^C-NMR (126 MHz, DMSO-*d*_6_) *δ*: 163.55, 158.19, 154.03, 143.70, 133.43, 129.61, 125.38, 124.22, 118.76, 116.78, 46.14, 43.72, 43.07, 38.73. ESI-HRMS calcd for C_14_H_15_N_2_O_3_^+^ ([M+H]^+^): 259.1077; found: 259.1088. The ^1^H-NMR, ^13^C-NMR, and HRMS spectra are presented in Supplementary Figures S32–S34.

3-(4-Benzylpiperazine-1-carbonyl)-2H-chromen-2-one (**9**)

White solid, 88 mg. Yield: 56%. Mp: 120–121 °C. ^1^H-NMR (CDCl_3_, 500 MHz, ppm) *δ*: 7.92 (s, 1H, Coumarin-4-H), 7.35–7.61 (m, 9H, Ar-H), 3.84 (s, 2H, CH_2_), 2.57–3.61 (m, 8H, Piperazine-H), 2.35 (s, 3H, CH_3_). ^13^C-NMR (126 MHz, CDCl_3_) *δ*: 163.34, 158.06, 154.08, 143.23, 132.91, 129.35, 128.60, 128.46, 124.97, 118.28, 116.81, 62.64, 52.83, 52.31, 46.02. ESI-HRMS calcd for C_21_H_21_N_2_O_3_^+^ ([M+H]^+^): 349.1547; found: 349.1557. The ^1^H-NMR, ^13^C-NMR, and HRMS spectra are presented in Supplementary Figures S35–S37.

7-Butoxy-*N*-(2-morpholinoethyl)-2-oxo-2H-chromene-3-carboxamide (**14a**)

White solid, 112 mg. Yield: 40%. Mp: 96–97 °C. ^1^H-NMR (CDCl_3_, 500 MHz, ppm) *δ*: 9.09 (s, 1H, CONH), 8.82 (s, 1H, Coumarin-4-H), 6.84–7.57 (m, 3H, Ar-H), 4.06 (t, 2H, *J* = 5.0 Hz, OCH_2_), 2.55, 3.77 (s, 8H, Morpholine-H), 3.59 (s, 2H, NHCH_2_), 2.63 (s, 2H, NHCH_2_CH_2_), 1.50–1.83 (m, 4H, 2CH_2_), 1.00 (t, 3H, *J* = 7.5 Hz, CH_3_). ^13^C-NMR (126 MHz, CDCl_3_) *δ*: 164.44, 162.12, 161.76, 156.71, 148.14, 130.85, 114.71, 114.31, 112.25, 100.75, 68.68, 66.98, 56.93, 53.42, 36.55, 30.90, 19.13, 13.75. ESI-HRMS calcd for C_20_H_27_N_2_O_5_^+^ ([M+H]^+^): 375.1914; found: 375.1924. The ^1^H-NMR, ^13^C-NMR, and HRMS spectra are presented in Supplementary Figures S38–S40.

*N*-(2-morpholinoethyl)-2-oxo-7-(pentyloxy)-2H-chromene-3-carboxamide (**14b**)

White solid, 105 mg. Yield: 32%. Mp: 75–76 °C. ^1^H-NMR (CDCl_3_, 500 MHz, ppm) *δ*: 9.09 (s, 1H, CONH), 8.82 (s, 1H, Coumarin-4-H), 6.84–7.58 (m, 3H, Ar-H), 4.05 (t, 2H, *J* = 5.0 Hz, OCH_2_), 2.58, 3.78 (s, 8H, Morpholine-H), 3.61 (s, 2H, NHCH_2_), 2.65 (s, 2H, NHCH_2_CH_2_), 1.39–1.85 (m, 6H, 3CH_2_), 0.95 (t, 3H, *J* = 7.5 Hz, CH_3_). ^13^C-NMR (126 MHz, CDCl_3_) *δ*: 164.47, 161.78, 156.73, 148.20, 130.88, 114.60, 114.34, 112.21, 100.73, 70.07, 68.99, 66.79, 56.86, 53.34, 36.35, 28.59, 28.06, 22.40, 22.35, 14.00. ESI-HRMS calcd for C_21_H_29_N_2_O_5_^+^ ([M+H]^+^): 389.2071; found: 389.2078. The ^1^H-NMR, ^13^C-NMR, and HRMS spectra are presented in Supplementary Figures S41–S43.

7-(Hexyloxy)-*N*-(2-morpholinoethyl)-2-oxo-2H-chromene-3-carboxamide (**14c**)

White solid, 112 mg. Yield: 31%. Mp: 96–97 °C. ^1^H-NMR (CDCl_3_, 500 MHz, ppm) *δ*: 9.09 (s, 1H, CONH), 8.82 (s, 1H, Coumarin-4-H), 6.84–7.58 (m, 3H, Ar-H), 4.05 (t, 2H, *J* = 5.0 Hz, OCH_2_), 2.57, 3.78 (s, 8H, Morpholine-H), 3.60 (s, 2H, NHCH_2_), 2.64 (s, 2H, NHCH_2_CH_2_), 1.35–1.85 (m, 8H, 4CH_2_), 0.92 (t, 3H, *J* = 7.5 Hz, CH_3_). ^13^C-NMR (126 MHz, CDCl_3_) *δ*: 164.43, 162.14, 161.75, 156.69, 148.15, 130.85, 114.62, 114.33, 112.20, 100.71, 70.07, 68.99, 66.85, 56.86, 53.36, 36.43, 31.48, 28.85, 25.59, 22.56, 14.02. ESI-HRMS calcd for C_22_H_31_N_2_O_5_^+^ ([M+H]^+^): 403.2227; found: 403.2232. The ^1^H-NMR, ^13^C-NMR, and HRMS spectra are presented in Supplementary Figures S44–S46.

7-(Heptyloxy)-*N*-(2-morpholinoethyl)-2-oxo-2H-chromene-3-carboxamide (**14d**)

White solid, 107 mg. Yield: 51%. Mp: 83–84 °C. ^1^H-NMR (CDCl_3_, 500 MHz, ppm) *δ*: 9.08 (t, 1H, *J* = 5.0 Hz, CONH), 8.82 (s, 1H, Coumarin-4-H), 6.83–7.57 (m, 3H, Ar-H), 4.05 (t, 2H, *J* = 5.0 Hz, OCH_2_), 2.54–3.77 (s, 8H, Morpholine-H), 3.58 (q, 2H, *J* = 6.7 Hz, NHCH_2_), 2.62 (t, 2H, *J* = 5.0 Hz, NHCH_2_CH_2_), 1.25–1.86 (m, 10H, 5CH_2_), 0.90 (t, 3H, *J* = 7.5 Hz, CH_3_). ^13^C-NMR (126 MHz, CDCl_3_) *δ*: 164.44, 162.12, 161.77, 156.72, 148.14, 130.85, 114.71, 114.32, 112.24, 100.75, 69.01, 66.97, 56.92, 53.41, 36.53, 31.71, 29.69, 28.96, 28.89, 25.88, 22.57, 14.05. ESI-HRMS calcd for C_23_H_33_N_2_O_5_^+^ ([M+H]^+^): 417.2384; found: 417.2393. The ^1^H-NMR, ^13^C-NMR, and HRMS spectra are presented in Supplementary Figures S47–S49.

*N*-(2-Morpholinoethyl)-7-(octyloxy)-2-oxo-2H-chromene-3-carboxamide (**14e**)

White solid, 103 mg. Yield: 52%. Mp: 86–87 °C. ^1^H-NMR (CDCl_3_, 500 MHz, ppm) *δ*: 9.08 (t, 1H, *J* = 5.0 Hz, CONH), 8.82 (s, 1H, Coumarin-4-H), 6.83–7.57 (m, 3H, Ar-H), 4.05 (t, 2H, *J* = 5.0 Hz, OCH_2_), 2.54–3.77 (s, 8H, Morpholine-H), 3.58 (q, 2H, *J* = 6.7 Hz, NHCH_2_), 2.61 (t, 2H, *J* = 5.0 Hz, NHCH_2_CH_2_), 1.26–1.86 (m, 12H, 6CH_2_), 0.89 (t, 3H, *J* = 7.5 Hz, CH_3_). ^13^C-NMR (126 MHz, CDCl_3_) *δ*: 164.42, 162.08, 161.75, 156.70, 148.11, 130.83, 114.71, 114.29, 112.23, 100.74, 69.00, 66.99, 56.92, 53.42, 36.54, 31.76, 29.26, 29.17, 28.89, 25.92, 22.63, 14.07. ESI-HRMS calcd for C_24_H_35_N_2_O_5_^+^ ([M+H]^+^): 431.2540; found: 431.2549. The ^1^H-NMR, ^13^C-NMR, and HRMS spectra are presented in Supplementary Figures S50–S52.

7-(Benzyloxy)-*N*-(2-morpholinoethyl)-2-oxo-2H-chromene-3-carboxamide (**14f**)

White solid, 92 mg. Yield: 50%. Mp: 165–166 °C. ^1^H-NMR (CDCl_3_, 500 MHz, ppm) *δ*: 8.95 (t, 1H, *J* = 5.0 Hz, CONH), 8.83 (s, 1H, Coumarin-4-H), 7.04–8.06 (m, 8H, Ar-H), 5.23 (s, 2H, OCH_2_), 2.55, 3.66 (m, 8H, Morpholine-H), 3.49 (q, 2H, *J* = 6.7 Hz, NHCH_2_), 2.48 (s, 2H, NHCH_2_CH_2_). ^13^C-NMR (126 MHz, CDCl_3_) *δ*: 161.58, 161.25, 154.42, 148.13, 133.95, 129.77, 125.22, 118.65, 116.62, 54.60, 54.13, 38.84, 23.56. ESI-HRMS calcd for C_23_H_25_N_2_O_5_^+^ ([M+H]^+^): 409.1758; found: 409.1763. The ^1^H-NMR, ^13^C-NMR, and HRMS spectra are presented in Supplementary Figures S53–S55.

7-((4-Fluorobenzyl)oxy)-*N*-(2-morpholinoethyl)-2-oxo-2H-chromene-3-carboxamide (**14g**)

White solid, 86 mg. Yield: 49%. Mp: 147–148 °C. ^1^H-NMR (CDCl_3_, 500 MHz, ppm) *δ*: 9.08 (t, 1H, *J* = 5.0 Hz, CONH), 8.82 (s, 1H, Coumarin-4-H), 6.91–7.60 (m, 7H, Ar-H), 5.12 (s, 2H, OCH_2_), 2.53, 3.76 (m, 8H, Morpholine-H), 3.57 (q, 2H, *J* = 6.7 Hz, NHCH_2_), 2.61 (t, 2H, *J* = 5.0 Hz, NHCH_2_CH_2_). ^13^C-NMR (126 MHz, CDCl_3_) *δ*: 163.56, 162.79 (d, *J* = 247.6 Hz), 161.93, 161.62, 156.54, 148.02, 131.11 (d, *J* = 2.9 Hz), 131.01, 129.56 (d, *J* = 8.3 Hz), 115.84 (d, *J* = 21.3 Hz), 115.19, 114.47, 112.68, 101.29, 70.10, 67.03, 56.86, 53.41, 36.54. ESI-HRMS calcd for C_23_H_24_FN_2_O_5_^+^ ([M+H]^+^): 427.1664; found: 427.1672. The ^1^H-NMR, ^13^C-NMR, and HRMS spectra are presented in Supplementary Figures S56–S58.

7-((4-Chlorobenzyl)oxy)-*N*-(2-morpholinoethyl)-2-oxo-2H-chromene-3-carboxamide (**14h**)

White solid, 112 mg. Yield: 61%. Mp: 112–113 °C. ^1^H-NMR (CDCl_3_, 500 MHz, ppm) *δ*: 9.08 (t, 1H, *J* = 5.0 Hz, CONH), 8.82 (s, 1H, Coumarin-4-H), 6.90–7.60 (m, 7H, Ar-H), 5.13 (s, 2H, OCH_2_), 2.55, 3.77 (m, 8H, Morpholine-H), 3.59 (s, 2H, NHCH_2_), 2.62 (s, 2H, NHCH_2_CH_2_). ^13^C-NMR (126 MHz, CDCl_3_) *δ*: 161.60, 161.26, 154.43, 148.13, 133.95, 129.77, 125.22, 118.65, 116.62, 54.60, 54.13, 38.85, 23.57. ESI-HRMS calcd for C_23_H_24_ClN_2_O_5_^+^ ([M+H]^+^): 443.1368; found: 443.1379. The ^1^H-NMR, ^13^C-NMR, and HRMS spectra are presented in Supplementary Figures S59–S61.

*N*-(2-morpholinoethyl)-2-oxo-7-((4-(trifluoromethyl)benzyl)oxy)-2H-chromene-3-carboxamide (**14i**)

White solid, 98 mg. Yield: 53%. Mp: 129–130 °C. ^1^H-NMR (CDCl_3_, 500 MHz, ppm) *δ*: 9.06 (t, 1H, *J* = 5.0 Hz, CONH), 8.83 (s, 1H, Coumarin-4-H), 6.90–7.69 (m, 7H, Ar-H), 5.23 (s, 2H, OCH_2_), 2.54, 3.76 (m, 8H, Morpholine-H), 3.59 (s, 2H, NHCH_2_), 2.62 (s, 2H, NHCH_2_CH_2_). ^13^C-NMR (126 MHz, CDCl_3_) *δ*: 163.28, 161.94, 161.51, 156.50, 147.97, 139.35, 131.09, 130.76 (q, *J* = 32.6 Hz), 127.49, 125.81 (q, *J* = 3.7 Hz), 123.91 (d, *J* = 272.0 Hz), 115.36, 114.36, 112.86, 101.38, 69.81, 66.81, 56.83, 53.35, 36.44, 29.69. ESI-HRMS calcd for C_24_H_24_F_3_N_2_O_5_^+^ ([M+H]^+^): 477.1632; found: 477.1640. The ^1^H-NMR, ^13^C-NMR, and HRMS spectra are presented in Supplementary Figures S62–S64.

7-((4-Methylbenzyl)oxy)-*N*-(2-morpholinoethyl)-2-oxo-2H-chromene-3-carboxamide (**14j**)

White solid, 106 mg. Yield: 42%. Mp: 186–187 °C. ^1^H-NMR (CDCl_3_, 500 MHz, ppm) *δ*: 9.08 (t, 1H, *J* = 5.0 Hz, CONH), 8.81 (s, 1H, Coumarin-4-H), 6.91–7.58 (m, 7H, Ar-H), 5.12 (s, 2H, OCH_2_), 2.57–3.78 (m, 8H, Morpholine-H), 3.59 (q, 2H, *J* = 6.7 Hz, NHCH_2_), 2.64 (t, 2H, *J* = 5.0 Hz, NHCH_2_CH_2_), 2.37 (s, 3H, CH_3_). ^13^C-NMR (126 MHz, CDCl_3_) *δ*: 163.93, 162.11, 161.69, 156.57, 148.11, 138.50, 132.27, 130.92, 129.51, 127.70, 114.90, 114.61, 112.51, 101.36, 70.79, 66.83, 56.88, 53.37, 36.45, 29.70, 21.23. ESI-HRMS calcd for C_24_H_27_N_2_O_5_^+^ ([M+H]^+^): 423.1914; found: 423.1924. The ^1^H-NMR, ^13^C-NMR, and HRMS spectra are presented in Supplementary Figures S65–S67.

### 2.2. Pharmacology

All animal experiments were approved by the Laboratory Animal Ethics Committee of Liaocheng University (No. AP2025022942; Approval Date: 17 March 2025). Prior to experiments, animals had free access to food and water. Compounds were administered via intravenous (i.v) or intraperitoneal (i.p) injection. Detailed pharmacological experimental methods are provided in the Supplementary Information. As positive controls, two widely recognized inhibitors were selected: the highly selective IDO1 inhibitor PF-0684003 and the selective TDO inhibitor LM10. The broad acceptance of these inhibitors within the field makes them a rigorous and reliable reference standard for assessing the efficacy of newly synthesized compounds.

#### 2.2.1. In Vitro IDO1 Activity

L-Tryptophan (400 μM) was added to a reaction mixture containing IDO1 protein (50 nM) and the test compound, followed by incubation at 37 °C for 60 min. After termination of the reaction, the supernatant was mixed with p-DMAB for color development, and the absorbance was measured at 490 nm [29]. Each experiment was performed in triplicate, and IC_50_ values were calculated using GraphPad Prism 8.4.3.

#### 2.2.2. In Vitro TDO Activity

L-Tryptophan (200 μM) was added to a reaction mixture containing TDO protein (50 nM) and the test compound, followed by incubation at 37 °C for 75 min. The change in absorbance at 321 nm was measured [30]. Each experiment was performed in triplicate, and IC_50_ values were calculated using GraphPad Prism 8.4.3.

#### 2.2.3. Cell Culture and Treatment

BV2 cells (Baidi Biotechnology Co., Ltd., Shanghai, China) were cultured in Dulbecco’s modified Eagle medium (DMEM) supplemented with 10% fetal bovine serum and 1% penicillin–streptomycin. Cells were seeded in six-well plates. When they reached 60–70% confluence, cells were pretreated with different concentrations of the test compound for 1 h, followed by induction with lipopolysaccharide (LPS) for 24 h.

#### 2.2.4. Detection of Cytokine mRNA Expression

Total RNA was extracted using Trizol and reverse transcribed into cDNA. The mRNA expression levels of target genes were detected by qRT-PCR. Primer sequences are listed in Supplementary Table S1.

#### 2.2.5. Western Blot Analysis

Cellular or tissue proteins were extracted, separated by SDS-PAGE, and transferred onto polyvinylidene fluoride (PVDF) membranes. After blocking, the membranes were incubated with corresponding primary antibodies (IDO1, TDO, PKA, BDNF, etc.) at 4 °C overnight, followed by incubation with horseradish peroxidase (HRP)-conjugated secondary antibodies at room temperature. Blots were visualized using chemiluminescence detection, and densitometric analysis was performed using Image J software Fiji (ImageJ 1.54p).

#### 2.2.6. Safety Evaluation

Mice were intraperitoneally injected with different doses (10, 20, and 30 mg/kg) of the compound for 14 consecutive days. Body weight changes were observed daily. After the final administration, the heart, liver, spleen, lungs, and kidneys were collected for hematoxylin and eosin (H&E) staining to assess histopathological changes.

#### 2.2.7. Pharmacokinetic Study

Mice were randomly divided into intravenous and intraperitoneal injection groups (total *n* = 9 per group) and administered the compound at a dose of 20 mg/kg. Blood samples were collected at different time points, and plasma was separated. After extraction with ethyl acetate, drug concentrations were determined by LC-MS/MS, and pharmacokinetic parameters were calculated using WinNonlin software 6.4.

#### 2.2.8. In Vivo Behavioral Experiments

Based on previous reports, an acute depression model was established in mice by intraperitoneal injection of LPS at a dose of 2 mg/kg [17,31,32]. Compound **14d** (20 mg/kg) was administered before and after LPS injection. The in vivo antidepressant activity of the target compound was evaluated using the forced swim test (FST) and the tail suspension test (TST) [33,34]. The open field test (OFT) was used to assess the effect of compound **14d** on spontaneous locomotor activity in mice [35].

#### 2.2.9. ELISA

Mouse brain tissue homogenates and serum were collected. Levels of 5-HT and KYN were detected using enzyme-linked immunosorbent assay (ELISA) kits according to the manufacturer’s instructions.

#### 2.2.10. Histopathology and Immunofluorescence Staining

Tissues were fixed, embedded, and sectioned. Histopathological changes were observed by H&E staining. For immunofluorescence staining, sections were blocked, incubated with corresponding primary antibodies, and then reacted with fluorescence-conjugated secondary antibodies. After counterstaining with DAPI, images were captured and quantitatively analyzed.

#### 2.2.11. Molecular Docking Study

Compound **14d** was selected for molecular docking using the CHARMM-based docking (CDOCKER) module in BIOVIA Discovery Studio (DS) 2021 software. The crystal structures of IDO1 (PDB ID: 5WHR) and TDO (PDB ID: 6PYZ) were used as receptors, and docking analysis was performed by defining the active site regions.

#### 2.2.12. Statistical Analysis

All data are presented as mean ± standard error of the mean (SEM). Each experiment was performed at least three times independently. Normality was assessed using the Shapiro–Wilk test, and homogeneity of variances was verified using the Brown-Forsythe test. Statistical significance was determined by one-way analysis of variance (ANOVA), followed by Dunnett’s post hoc test for multiple comparisons against the control group. All analyses were performed using GraphPad Prism software. A value of *p* < 0.05 was considered statistically significant. “#” indicated comparison with the control group, “*” indicated comparison with the model group.

## 3. Results and Discussion

### 3.1. Chemistry

**Scheme 1:**
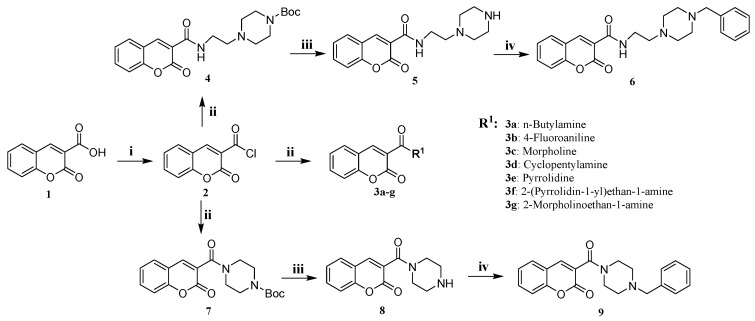
Reagents and conditions: (i) SOCl_2_, DMF, CH_2_Cl_2_, 50 °C, 3 h; (ii) NH_2_-R^1^, Triethylamine, CH_2_Cl_2_, 0 °C, 2 h; (iii) CF_3_COOH, CH_2_Cl_2_, Room temperature, 1 h; (iv) PhCH_2_Br, Triethylamine, CH_3_CN, Room temperature, 3 h. Synthesis of the target compounds **3a**–**g**, **5**–**6**, and **8**–**9**.

The synthetic routes for target compounds **3a**–**g**, **5**–**6**, and **8**–**9** are outlined in Scheme 1. Starting from 3-carboxycoumarin, an acyl chlorination reaction with thionyl chloride afforded intermediate **2**, which was used directly in the subsequent step without further purification. Intermediate **2** was then reacted with amine in the presence of an organic weak base to yield target compounds **3a**–**g** as well as intermediates **4** and **7**. Intermediates **4** and **7** were subjected to removal of the *N*-Boc protecting group under acidic conditions to give compounds **5** and **8**, respectively. Finally, compounds **5** and **8** were treated with benzyl bromide under basic conditions to afford target compounds **6** and **9**, respectively.

The structures of target compounds **3a**–**g** were confirmed by ^1^H-NMR, ^13^C-NMR, and HRMS. Taking *N*-butyl-2-oxo-2*H*-chromene-3-carboxamide (**3a**) as a representative example, its ^1^H-NMR spectrum exhibited the following characteristic signals: a singlet at δ 8.92 ppm (s, 1H) corresponding to the H-4 proton of the coumarin moiety; a broad singlet at δ 8.80 ppm (s, 1H) assigned to the amide NH proton; a multiplet at δ 7.35–7.71 ppm (m, 4H) for the aromatic protons on the coumarin benzene ring; a quartet at δ 3.45–3.49 ppm (q, 2H) for the methylene (N-CH_2_) protons adjacent to the NH group; a multiplet at δ 1.41–1.66 ppm (m, 4H) for the two central methylene (-CH_2_-) protons of the butyl chain; and a triplet at δ 0.95–0.98 ppm (t, 3H) for the terminal methyl (-CH_3_) protons. In the ^13^C-NMR spectrum, the signals at δ 161.50 and 161.40 ppm were assigned to the carbonyl carbons; the signals in the range of δ 116.61–154.42 ppm were assigned to the carbons of the coumarin skeleton; and the signals in the range of δ 13.76–39.65 ppm were assigned to the carbons of the butyl chain. HRMS analysis showed that the measured value (268.0954) matched well with the calculated molecular weight (268.0944), with a deviation of less than 0.001. These spectral data were consistent with the proposed structures.

**Scheme 2:**
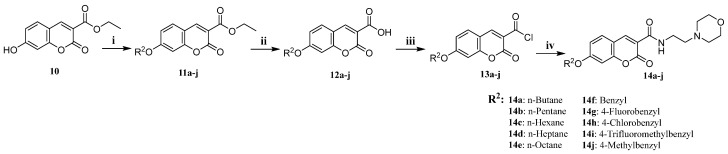
Reagents and conditions: (i) R^2^Br, K_2_CO_3_, KI, Acetone, 55 °C, 4 h; (ii) NaOH, H_2_O/CH_3_OH, 55 °C, 2 h; (iii) SOCl_2_, DMF, CH_2_Cl_2_, 50 °C, 3 h; (iv) 2-Morpholinoethan-1-amine, Triethylamine, CH_2_Cl_2_, 0 °C, 2 h. Synthesis of the target compounds **14a**–**j**.

The synthetic routes for target compounds **14a**–**j** are shown in Scheme 2. Ethyl 7-hydroxy-2-oxo-2H-chromene-3-carboxylate (**10**) was used as the starting material and reacted with various bromides under basic conditions to furnish intermediates **11a**–**j**. Subsequently, intermediates **11a**–**j** underwent ester hydrolysis under basic conditions to generate intermediates **12a**–**j**. These were then converted to intermediates **13a**–**j** via acyl chlorination with thionyl chloride, and the resulting products were used directly in the next step without purification. Finally, intermediates **13a**–**j** were reacted with 2-morpholinoethan-1-amine in the presence of an organic weak base to yield the target compounds **14a**–**j**.

The structures of target compounds **14a**–**j** were confirmed by ^1^H-NMR, ^13^C-NMR, and HRMS. Taking 7-butoxy-*N*-(2-morpholinoethyl)-2-oxo-2H-chromene-3-carboxamide (**14a**) as a representative example, its ^1^H-NMR spectrum exhibited characteristic signals consistent with the structural features. Specifically, a singlet at δ 9.09 ppm (s, 1H) was assigned to the H-4 proton of the coumarin moiety; a singlet at δ 8.82 ppm (s, 1H) corresponded to the amide NH proton; and a multiplet at δ 6.84–7.57 ppm (m, 3H) was attributed to the aromatic protons on the coumarin benzene ring. These proton signals were similar to those observed for compound 3a in terms of splitting patterns and integration, albeit with slight chemical shift variations due to the different substituent effects. In the ^13^C-NMR spectrum, the signals in the range of δ 100.75–164.44 ppm were assigned to the carbons of the coumarin skeleton and the carbonyl carbons, while the signals at δ 13.75–68.68 ppm were attributed to the carbons of the butyl chain and the 2-morpholinoethan-1-amine moiety. HRMS analysis showed that the found value (375.1924) matched well with the calculated molecular weight (375.1914), with a deviation of less than 0.001. These spectral data were fully consistent with the proposed structures.

### 3.2. Pharmacology

#### 3.2.1. IDO1/TDO Enzyme Inhibitory Activity

Based on literature reports and our preliminary experiments [17], a concentration of 50 μM was selected for the initial screening, as it reliably yielded stable and comparable inhibition rates under the current experimental conditions; the inhibitory activities of all target compounds against IDO1 and TDO at this concentration are summarized in Table 1. Compounds with an IDO1 inhibitory rate exceeding 80% included **3g** (90%), **14b** (94%), **14d** (92%), and **14f** (91%), among which **14b**, **14d** and **14f** exhibited inhibitory rates close to that of the positive control IDO1 inhibitor PF-0684003 (98%). Compounds with a TDO inhibitory rate exceeding 80% were **3g** (86%), **14b** (87%), and **14d** (98%), with compound **14d** showing an inhibitory rate comparable to that of the positive control TDO inhibitor LM10 (98%). The remaining compounds (e.g., **3a**–**f**, **5**–**6**, **8**–**9**, **14a**, **14c**, **14e** and **14g**–**j**) exhibited weak inhibitory activity, indicating that different substituents significantly affect the inhibitory activity. Based on the results in Table 1, compounds **3g**, **14b**, **14d** and **14f**, which showed relatively good inhibitory activity, were selected for further determination of their IC_50_ values. The experimental results are shown in Table 2.

As seen in Table 2, compound **14d** exhibited IC_50_ values of 0.34 μM against IDO1 and 0.75 μM against TDO. Although these values are higher than those of the positive controls PF-0684003 (IDO1, IC_50_ = 0.30 μM) and LM10 (TDO, IC_50_ = 0.51 μM), compound **14d** still demonstrated favorable dual-target inhibitory activity. Although compound **3g** showed a high inhibitory rate in the primary screening, its IC_50_ values were in the range of 4–7 μM, indicating moderate activity. Compound **14b** also exhibited good inhibitory activity against both IDO1 and TDO, with IC_50_ values of 0.94 μM and 1.12 μM, respectively. Compound **14f** showed potent inhibition against IDO1 (IC_50_ = 2.40 μM) and weak activity against TDO, displaying a selective IDO1 inhibitory profile.

A preliminary structure-activity relationship analysis was performed based on the experimental results in Table 1. In this study, using the coumarin scaffold of lead compound **I** as the core, a series of dual IDO1/TDO inhibitors **3a**–**g**, **5**–**6**, and **8**–**9** were designed and synthesized via a molecular hybridization strategy. Pharmacological results indicated that, except for compound **3g**, which exhibited potent dual IDO1/TDO inhibitory activity, most compounds in this series showed weak inhibitory activity. Structural analysis suggested that the 2-morpholinoethan-1-amine fragment in compound **3g** corresponds to that in moclobemide, and it is presumed that this fragment is a key moiety responsible for the activity. Furthermore, in this series, variations in the R^1^ group did not show a clear trend in influencing activity.

Based on compound **3g** as the most active hit compound, the 2-morpholinoethan-1-amine and coumarin moieties were retained, and molecular docking was performed to predict their binding modes in the active pockets of IDO1 and TDO, followed by further structural modifications to yield the series of compounds **14a**–**j**. Pharmacological results revealed that among the alkyl chain (C_4_-C_8_) substituted compounds, upon increasing the chain length from C_4_ to C_8_, C_5_ and C_7_ displayed the most potent IDO1 inhibition; however, their overall inhibitory activity was superior to that of the benzyl-substituted derivatives. In the benzyl-substituted series, compound **14f**, bearing an unsubstituted benzyl phenyl ring, exhibited better activity than those containing electron-withdrawing groups (F, Cl, CF_3_) or electron-donating groups (CH_3_) on the phenyl ring. It is hypothesized that either increasing or decreasing the electron density of the phenyl ring is detrimental to activity, suggesting that this position may not be involved in charge-related key interactions.

Overall, the inhibitory activity of the modified **14a**–**j** series was superior to that of the **3a**–**g**, **5**–**6**, and **8**–**9** series. It is presumed that the structural modifications enhanced the interaction between the compounds and the enzyme active centers, enabling them to spatially and electronically adapt to the active pockets of both IDO1 and TDO simultaneously, thereby achieving dual-target inhibition. This series of compounds holds potential for further development as antidepressant agents and provides a clear direction for subsequent compound design and optimization.

#### 3.2.2. Evaluation of Anti-Inflammatory Effects in an LPS-Induced in Vitro Inflammation Model

**Figure 3:**
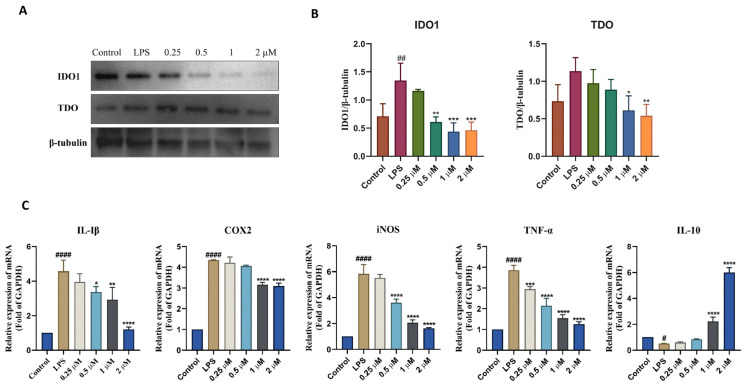
Anti-inflammatory effects of compound **14d** in an LPS-induced microglial inflammation model: (**A**,**B**) Western blotting was used to analyze the expression of related protein levels in cells after treatment with compound **14d**; (**C**) Inhibition of pro-inflammatory cytokine (COX-2, iNOS, TNF-α, and IL-1β) production and upregulation of the anti-inflammatory cytokine IL-10 level. Data are expressed as mean ± SEM; ^#^ *p* < 0.05, ^##^ *p* < 0.01, ^####^ *p* < 0.0001 vs. control group; * *p* < 0.05, ** *p* < 0.01, *** *p* < 0.001, **** *p* < 0.0001 vs. LPS group.

Based on the in vitro screening results shown in Table 1 and Table 2, compound **14d**, which exhibited the best activity, was selected for further investigation of its modulatory effects on inflammatory responses and its target regulatory mechanisms in an LPS-induced microglial activation model. First, the cytotoxicity of compound **14d** was evaluated using six concentrations (0.25, 0.5, 1, 2, 4, and 8 μM). The results showed that mild cytotoxicity was observed only at the high concentration of 8 μM (Supplementary Figure S1). Therefore, four concentrations (0.25, 0.5, 1, and 2 μM) were selected for subsequent cell-based experiments. An inflammatory model was established by stimulating BV2 cells with LPS (100 ng/mL). The results showed that LPS treatment significantly upregulated the expression of IDO1 and TDO at both the mRNA and protein levels, whereas compound **14d** markedly reversed this trend (Figure 3A,B and Supplementary Figure S2), indicating its effective regulation of key targets in the TRY metabolic pathway.

Furthermore, compound **14d** significantly suppressed the expression of the pro-inflammatory cytokines interleukin-1β (IL-1β), cyclooxygenase-2 (COX-2), inducible nitric oxide synthase (iNOS), and tumor necrosis factor-alpha (TNF-α) induced by LPS, while upregulating the level of the anti-inflammatory cytokine IL-10 (Figure 3C). Collectively, these results demonstrate that compound **14d** effectively alleviates LPS-induced excessive microglial activation by inhibiting the production of pro-inflammatory factors and interfering with key steps in TRY metabolism, suggesting its potential application value in the intervention of neuroinflammation associated with depression.

#### 3.2.3. In Vivo Safety Evaluation

**Figure 4:**
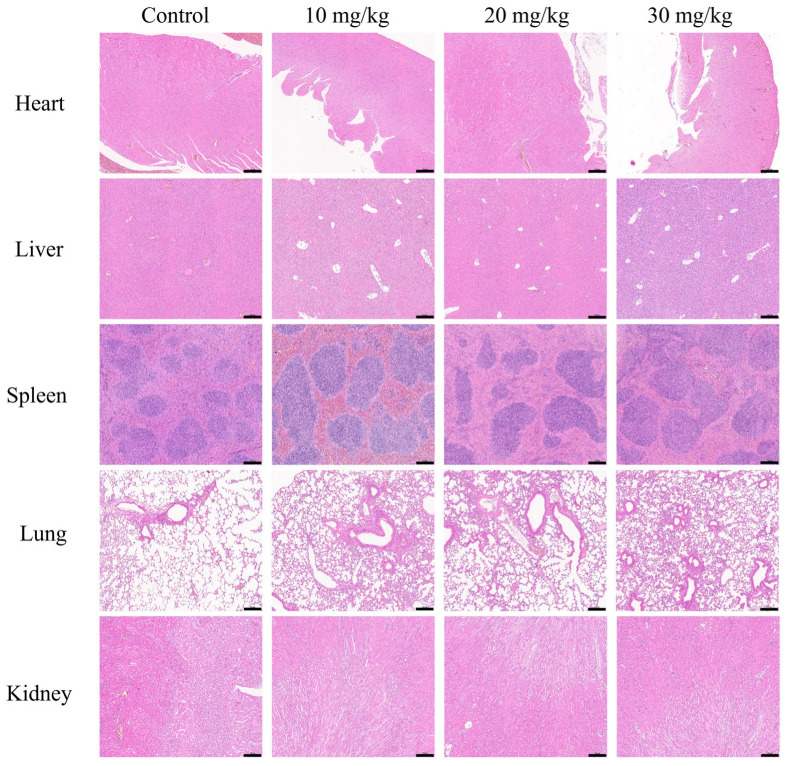
Histopathological evaluation of major organs (heart, liver, spleen, lung, and kidney) from mice by H&E staining to observe acute toxicity symptoms. Bar = 200 μm.

To clarify the in vivo safety profile of compound **14d** and determine an appropriate dose range for subsequent in-depth studies, three dose levels (10, 20, and 30 mg/kg) were administered to male C57BL/6J mice via intraperitoneal (i.p.) injection once daily for 14 consecutive days. Body weight changes were monitored daily during the experiment. On day 14, the animals were sacrificed, and heart, liver, spleen, lung, and kidney tissues were collected for histopathological evaluation by H&E staining. The results (Figure 4) showed that in the 10 and 20 mg/kg dose groups, body weight did not fluctuate significantly, and the major organ structures remained normal without obvious pathological changes. In contrast, the 30 mg/kg group exhibited a gradual decrease in body weight over the dosing period and showed mild liver injury, indicating toxicity at this dose (Supplementary Figure S3, daily body weight dynamics after administration). Based on these results, 20 mg/kg was determined as the safe and effective dose of compound **14d** for subsequent antidepressant studies.

#### 3.2.4. Pharmacokinetic Study

The pharmacokinetic profile of compound **14d** was evaluated in male mice following i.p. and i.v. administration at a dose of 20 mg/kg, with three animals per group (Table 3). In terms of absorption, after i.p. injection, compound **14d** reached peak concentration rapidly, with a time to maximum concentration (T_max_) of 0.5 h and a peak concentration (C_max_) of 482 ng/mL. After i.v. injection, the C_max_ was 628 ng/mL, consistent with the immediate distribution characteristics of i.v. administration. The absolute bioavailability (F) after i.p. administration was 73%. Regarding elimination and distribution, the elimination half-life (T_1/2_) was 22.33 h after i.p. injection and 39.10 h after i.v. injection. The relatively large standard deviations indicate inter-individual variability in elimination, but the half-lives differed between the two routes. The clearance (Cl) was 688 mL/h/kg for the i.p. group and 329 mL/h/kg for the i.v. group. The volume of distribution (V_z_) was 21,514 mL/kg for the i.p. group and 16,563 mL/kg for the i.v. group. Overall, compound **14d** exhibited favorable pharmacokinetic properties in mice, including rapid absorption after i.p. injection, high bioavailability, and a long half-life, indicating good pharmacokinetic characteristics and potential for further development.

#### 3.2.5. In Vivo Antidepressant Activity

**Figure 5:**
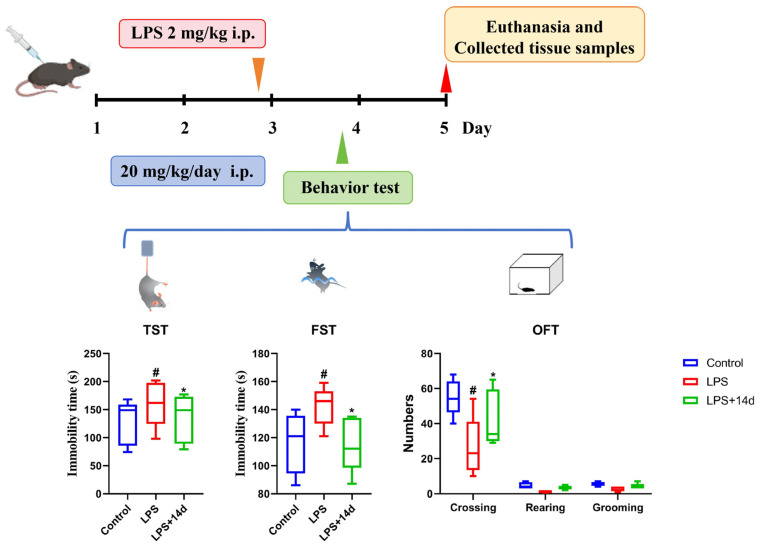
Experimental design for assessing antidepressant-like behavior of compound **14d** and results of the FST, TST, and OFT. Data are expressed as mean ± SEM; ^#^ *p* < 0.05 vs. control group; * *p* < 0.05 vs. LPS group.

To systematically evaluate the in vivo antidepressant effect of compound **14d**, an acute depression-like behavior model was established in mice by i.p. injection of LPS (2 mg/kg) (Figure 5). Compound **14d** was administered once daily at a dose of 20 mg/kg. Antidepressant activity was assessed using two classic behavioral paradigms, the TST and FST, while the OFT was employed to evaluate the effect on spontaneous locomotor activity. The results showed that LPS induction led to typical depression-like behaviors in mice, characterized by a significant increase in immobility time in both the FST and TST. Intervention with compound **14d** significantly reduced the immobility time. Furthermore, in the OFT, LPS caused a decrease in spontaneous locomotor activity, reflected by reduced frequencies of crossing, rearing, and grooming. Compound **14d** effectively improved these parameters. Collectively, compound **14d** exhibited significant antidepressant activity in vivo.

#### 3.2.6. Effects of Compound **14d** on Microglia In Vivo

**Figure 6:**
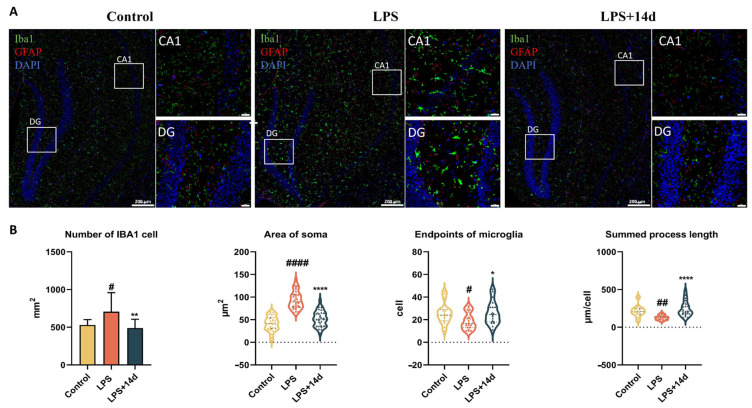
(**A**) In an LPS-induced mouse model, compound **14d** inhibits excessive microglial activation in the hippocampal dentate gyrus. (**B**) In an LPS-induced mouse model, compound **14d** reverses the increase in microglial cell number and soma hypertrophy in the mouse hippocampal DG region following LPS stimulation and increases process length and the number of endpoints. Data are expressed as mean ± SEM; ^#^ *p* < 0.05, ^##^ *p* < 0.01, ^####^ *p* < 0.0001 vs. control group; * *p* < 0.05, ** *p* < 0.01, **** *p* < 0.0001 vs. LPS group. Bar = 25 μm.

Accumulating evidence indicates that inflammatory responses (both central and peripheral) and dysregulation of the TRY-KYN metabolic pathway together constitute important pathological bases for the onset of depression [36]. To explore the relationship between the antidepressant effect of compound **14d** and central inflammation, this study analyzed the morphological and quantitative changes in microglia within the mouse hippocampal dentate gyrus (DG). The experimental results (Figure 6) showed that LPS stimulation induced marked microglial activation in the DG region, manifested by an increased number of microglia, enlarged cell bodies, shortened processes, and a reduced number of endpoints. Following compound **14d** intervention, all these parameters were significantly improved, and both the number and morphology of microglia tended toward normalization. In summary, compound **14d** significantly alleviated LPS-induced depression-like behavior, and its mechanism of action is closely associated with the inhibition of excessive microglial activation in the hippocampus.

#### 3.2.7. Regulation of Inflammatory Cytokines and Targets

**Figure 7:**
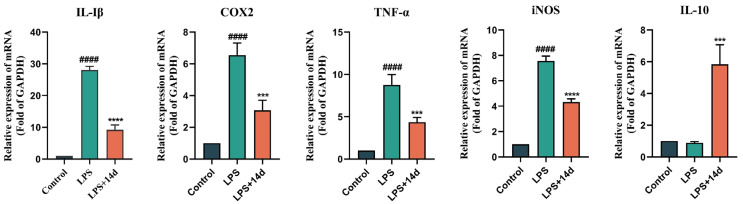
Compound **14d** exerts a dual modulatory effect on inflammatory cytokines in mouse brain: it suppresses the mRNA expression of pro-inflammatory cytokines COX-2, iNOS, TNF-α, and IL-1β, while upregulating the level of the anti-inflammatory cytokine IL-10. Data are expressed as mean ± SEM; ^####^ *p* < 0.0001 vs. control group; *** *p* < 0.001, **** *p* < 0.0001 vs. LPS group.

To clarify the in vivo anti-inflammatory effect of compound **14d**, the mRNA expression levels of inflammatory factors including COX-2, iNOS, TNF-α, and IL-1β were examined in brain tissues using the LPS-induced mouse model. The results demonstrated that compound **14d** significantly reduced the transcriptional levels of these pro-inflammatory cytokines (Figure 7) while elevating the level of the anti-inflammatory cytokine IL-10. These findings are consistent with the in vitro results obtained in BV2 cells, indicating that compound **14d** exerts a stable anti-inflammatory effect both in vitro and in vivo.

**Figure 8:**
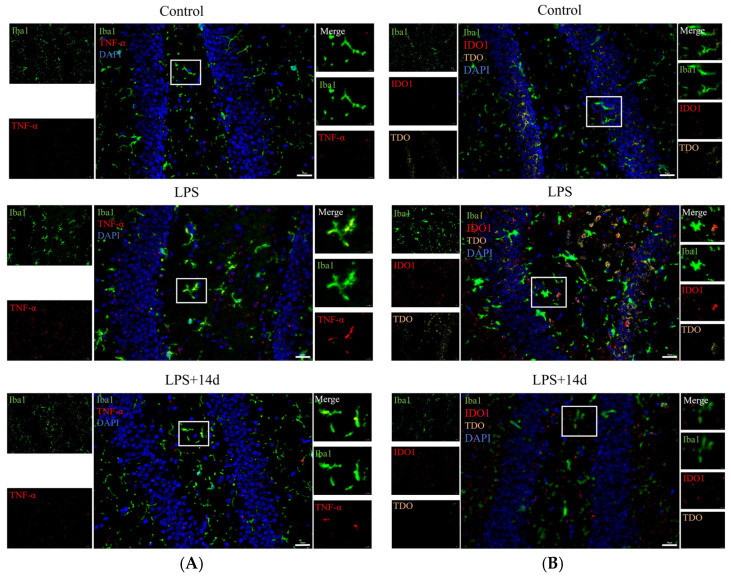
In an LPS-induced mouse model, compound **14d** inhibits neuroinflammatory responses, as evidenced by: (**A**) A significant reduction in the co-localization of microglia with the pro-inflammatory cytokine TNF-α in the hippocampus; (**B**) Immunofluorescence detection showing that compound **14d** effectively reverses LPS-induced co-expression of IDO1 and TDO with activated microglia. Bar = 25 μm.

Based on immunofluorescence co-localization analysis, compound **14d** markedly decreased the co-localization of microglia with the pro-inflammatory cytokine TNF-α in the mouse hippocampus (Figure 8A), suggesting its ability to inhibit microglia-mediated release of inflammatory cytokines. Concurrently, compound **14d** effectively reversed the LPS-induced co-localization of IDO1 and TDO with microglia (Figure 8B) and significantly reduced the mRNA levels of IDO1 and TDO in hippocampal tissues (Supplementary Figure S4). These results are consistent with the trend observed in the in vitro target modulation experiments. Taken together, compound **14d** attenuates the production of inflammatory cytokines and alleviates excessive microglial activation by inhibiting the activity of IDO1 and TDO, thereby demonstrating promising potential for suppressing neuroinflammation.

#### 3.2.8. Effects on KYN and 5-HT Levels in Mouse Brain

**Figure 9:**
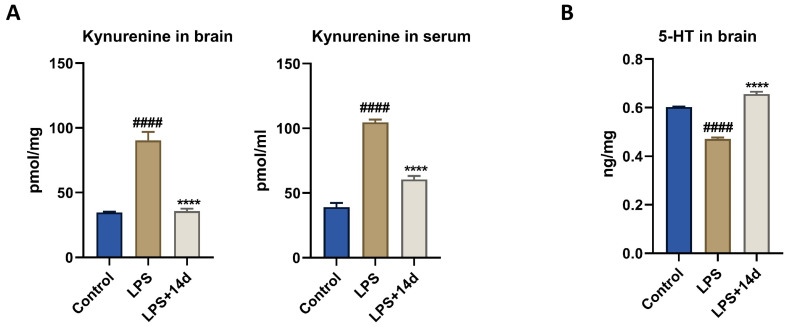
(**A**) In an LPS-induced mouse model, compound **14d** significantly reduces KYN levels in both brain and serum; (**B**) Compound **14d** significantly elevates 5-HT levels in mouse brain. Data are expressed as mean ± SEM; ^####^ *p* < 0.0001 vs. control group; **** *p* < 0.0001 vs. LPS group.

In the TRY metabolic pathway, abnormally elevated KYN levels accompanied by decreased 5-HT levels represent a typical metabolic feature of inflammation-associated depression. The core mechanism involves the activation of IDO1/TDO enzymes by inflammatory cytokines, which redirects TRY metabolism from the 5-HT synthesis pathway toward KYN production, while simultaneously shifting downstream metabolites toward neurotoxicity. This mechanism not only explains how immune activation affects monoaminergic neurotransmission but also provides a theoretical basis for developing novel antidepressant strategies targeting the KYN pathway [37,38]. The results of this study showed that LPS stimulation significantly increased KYN levels in both brain tissue and serum of mice, whereas intervention with compound **14d** markedly reduced KYN levels in both compartments (Figure 9A). Concurrently, LPS treatment caused a significant decrease in cerebral 5-HT levels, which was effectively reversed by compound **14d**, leading to a substantial recovery of 5-HT content (Figure 9B). Given that 5-HT is a key neurotransmitter associated with depression in the central nervous system, restoration of its levels helps correct the neurotransmitter imbalance observed in depressive states. Thus, the antidepressant effect of compound **14d** is closely linked to its modulation of the balance between cerebral KYN and 5-HT levels.

#### 3.2.9. Regulation of LPS-Induced BDNF and PKA Protein Expression in Mouse Hippocampus

**Figure 10:**
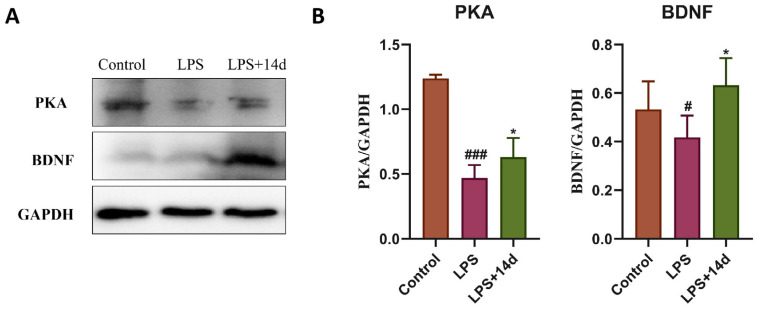
(**A**,**B**) Western blotting was used to analyze the expression of BDNF and PKA levels in mouse brain after treatment with compound **14d**. Data are expressed as mean ± SEM; ^#^ *p* < 0.05, ^###^ *p* < 0.001 vs. control group; * *p* < 0.05 vs. LPS group.

Brain-derived neurotrophic factor (BDNF) and protein kinase A (PKA)-mediated signaling pathways are two critically important regulatory pathways in the nervous system. These pathways do not function independently; instead, they jointly regulate the expression, phosphorylation status, and subcellular localization of proteins involved in neural function, thereby influencing neuronal survival, synaptic plasticity, dendritic development, and processes such as learning and memory. In neuropsychiatric disorders (e.g., depression and Alzheimer’s disease), decreased levels of both PKA and BDNF are often observed concurrently. Therefore, the development of drugs that target both pathways is considered a promising strategy [39,40]. Using Western blot analysis (Figure 10), this study found that LPS stimulation significantly downregulated the protein expression levels of BDNF and PKA in mouse hippocampal tissues. Intervention with compound **14d** significantly reversed this downregulation, indicating that this compound restores the expression of key molecules that regulate central nervous system function.

In summary, compound **14d** simultaneously inhibits IDO1/TDO expression, alleviates neuroinflammation, reduces KYN levels, restores 5-HT content, and upregulates BDNF/PKA protein levels. These changes occur in parallel with the amelioration of depression-like behaviors, suggesting that its antidepressant effect is likely achieved through the synergistic action of multiple mechanisms, among which dual-target inhibition of IDO1/TDO may be one of the key upstream mechanisms.

#### 3.2.10. Molecular Docking Studies

**Figure 11:**
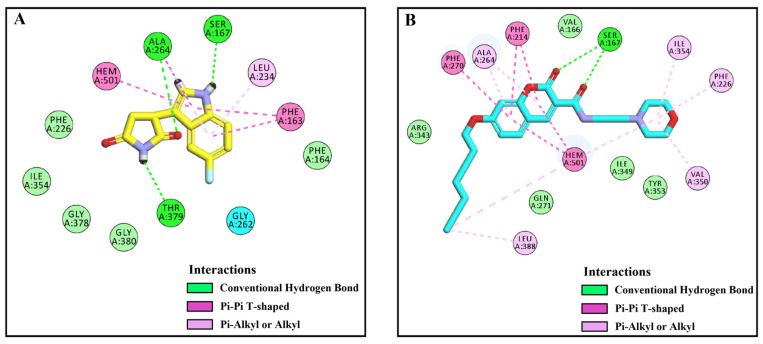
(**A**,**B**) Predicted binding modes of PF-0684003 and compound **14d** to IDO1.

**Figure 12:**
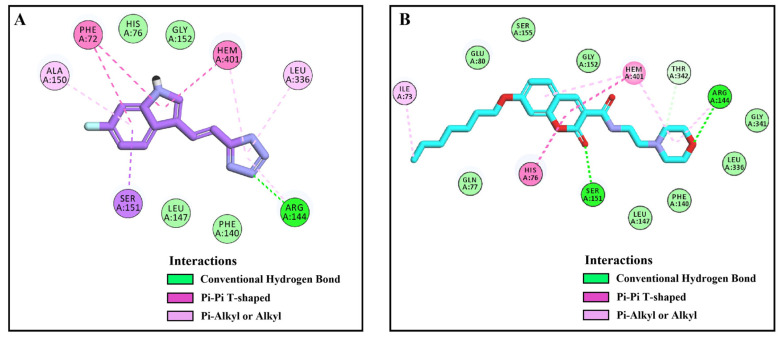
(**A**,**B**) Predicted binding modes of LM10 and compound **14d** to TDO.

Compound **14d** was subjected to molecular docking analysis using the CDOCKER module of DS software to explore its possible binding modes with IDO1 and TDO. The IDO1 inhibitor PF-0684003 and the TDO inhibitor LM10 were also included as controls. For detailed results, see Figure 11 and Figure 12.

The docking results showed that compound **14d** and PF-0684003 form interactions with the active sites of IDO1 (Figure 11). The docking results revealed that PF-0684003 forms hydrogen bonds with SER167, ALA264, and THR379, as well as pi-pi interactions with HEM501 and PHE163 (Figure 11A, 2D image). For compound **14d**, the docking results showed that it forms a hydrogen bond with SER167 and pi-pi interactions with residues such as HEM501, PHE270, and PHE214 (Figure 11B, 2D image). Although compound **14d** forms fewer hydrogen bonds than PF-0684003, it has more pi-pi and pi-alkyl interactions than PF-0684003, which may enable it to form more total interactions with IDO1.

The docking results showed that compound **14d** and LM10 form interactions with the active sites of TDO (Figure 12). The docking results with TDO revealed that LM10 forms a hydrogen bond with ARG144, as well as pi-pi interactions with HEM401 and PHE72 (Figure 12A, 2D image). The docking results with TDO showed that compound **14d** forms two hydrogen bonds with ARG144 and SER151, as well as pi-pi interactions with HIS76 and HEM401 (Figure 12B, 2D image). Compared with LM10, compound **14d** forms more hydrogen bonds. These docking findings imply that the ability of compound **14d** to establish multiple interactions with TDO (and with IDO1, as shown previously) could be an important factor underlying its promising pharmacological activity.

## 4. Conclusions

In this study, using coumarin as the lead compound and incorporating the 2-morpholinoethan-1-amine fragment from the antidepressant moclobemide, a series of novel morpholine–coumarin derivatives were designed and synthesized via molecular hybridization and structure optimization strategies. In vitro IDO1/TDO enzyme activity screening revealed that compound **14d** exhibited favorable dual-target inhibitory activity against both IDO1 and TDO. In an LPS-induced BV2 cell inflammation model, compound **14d** significantly downregulated the protein and mRNA levels of IDO1 and TDO, suppressed the production of pro-inflammatory cytokines (IL-1β, COX-2, iNOS, TNF-α), and upregulated the level of the anti-inflammatory cytokine IL-10, demonstrating potent in vitro anti-neuroinflammatory effects. In vivo safety evaluation showed no obvious toxicity to major organs in mice at a dose of 20 mg/kg, and compound **14d** exhibited favorable pharmacokinetic properties following intraperitoneal administration. In an LPS-induced acute depression mouse model, compound **14d** significantly reduced immobility time in the FST and TST and improved spontaneous locomotor activity in the OFT, displaying significant antidepressant effects. Further mechanistic studies revealed that compound **14d** effectively inhibited excessive microglial activation in the hippocampal dentate gyrus, reduced cerebral levels of pro-inflammatory cytokines and KYN, restored 5-HT content, and upregulated the protein expression of BDNF and PKA. Molecular docking analysis confirmed that compound **14d** forms stable hydrogen bonds and hydrophobic interactions with the active sites of IDO1 and TDO.

In summary, this study has identified a novel morpholine–coumarin derivative, **14d**, with a favorable safety profile and good pharmacokinetic properties, which can serve as a dual-target antidepressant lead compound against IDO1/TDO, providing a foundation for subsequent structural optimization and in-depth mechanistic studies.

## Figures and Tables

**Table 1:** In vitro inhibition of IDO1 and TDO by the target compounds.

Compounds	R (R^1^/R^2^)	Inhibition at 50 µM (%) ^a^	
		IDO1	TDO
**3a**	-NHC_4_H_9_	11	15
**3b**	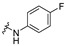	12	44
**3c**	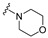	60	48
**3d**	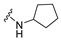	9	39
**3e**	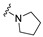	12	44
**3f**	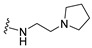	13	42
**3g**	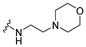	90	86
**5**	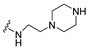	44	34
**6**	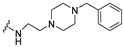	15	48
**8**	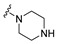	48	39
**9**	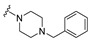	10	29
**14a**	-C_4_H_9_	55	29
**14b**	-C_5_H_11_	94	87
**14c**	-C_6_H_13_	66	28
**14d**	-C_7_H_15_	92	98
**14e**	-C_8_H_17_	32	68
**14f**	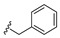	91	40
**14g**	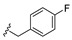	59	21
**14h**	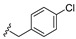	23	52
**14i**	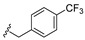	62	38
**14j**	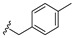	53	27
**PF-0684003**		98	/
**LM10**		/	98

^a^ The percentage inhibition values at 50 μM are the means of three independent experiments; “/” indicates not tested.

**Table 2:** IC_50_ values of selected compounds against IDO1 and TDO.

Compounds	IC_50_ (µM) ^a^	
	IDO1	TDO
**3g**	4.09 ± 0.56	6.20 ± 0.13
**14b**	0.94 ± 0.14	1.12 ± 0.15
**14d**	0.34 ± 0.06	0.75 ± 0.10
**14f**	2.40 ± 0.11	/
**PF-0684003**	0.30 ± 0.04	/
**LM10**	/	0.51 ± 0.07

^a^ IC_50_ values are expressed as mean ± SD from three independent experiments; “/” not determined.

**Table 3:** Pharmacokinetic parameters of compound **14d**.

Pharmacokinetic Property (Units) ^a^	i.p. (20 mg/kg)	i.v. (20 mg/kg)
T_1/2_ (h)	22.33 ± 5.11	39.10 ± 18.41
T_max_ (h)	0.50	0.25
C_max_ (ng/mL)	482 ± 3	628 ± 9
Bioavailability F (%)	73	100
V_z_ (mL/kg)	21,514 ± 403	16,563 ± 582
Cl (mL/h/kg)	688 ± 130	329 ± 114
AUC_0–t_ (h.ng/mL)	2473 ± 15	3395 ± 7
AUC_0–∞_ (h.ng/mL)	14,935 ± 3122	33,725 ± 14,584
AUMC_0–t_ (h^2^.ng/mL)	7354 ± 83	9889 ± 55
AUMC_0–∞_ (h^2^.ng/mL)	498,842 ± 222,883	2,160,901 ± 1,938,746
MRT_0–t_ (h)	2.97 ± 0.02	2.91 ± 0.01
MRT_0–∞_ (h)	32.38 ± 7.34	56.44 ± 26.47

^a^ Experiments were conducted in mice (*n* = 9). Data are mean ± SD values.

## Data Availability

The original contributions presented in this study are included in the article and Supplementary Materials. Further inquiries can be directed to the corresponding author.

## Supplementary Materials

The following supporting information can be downloaded at https://www.mdpi.com/article/10.3390/biom16071002/s1. The original Western blot images can be found in the Supplementary Materials.

## Abbreviations

The following abbreviations are used in this manuscript:

ALAAD	Aromatic L-amino acid decarboxylase
BDNF	Brain-derived neurotrophic factor
Cmax	Peak concentration
COX-2	Cyclooxygenase-2
CDOCKER	CHARMM-based docking
DG	Dentate gyrus
DMEM	Dulbecco’s modified Eagle medium
ELISA	Enzyme-linked immunosorbent assay
FST	Forced swim test
H&E	Hematoxylin and eosin
HRP	Horseradish peroxidase
HRMS	High-resolution mass spectrometry
5-HT	5-Hydroxytryptamine or Serotonin
5-HTP	5-Hydroxytryptophan
iNOS	Inducible nitric oxide synthase
i.p	Intraperitoneal
i.v	Intravenous
IC_50_	Half maximal inhibitory concentration
IDO	Indoleamine 2,3-dioxygenase
IL-1β	Cytokines interleukin-1β
KAT	Kynurenine aminotransferase
KMO	Kynurenine 3-monooxygenase
KYN	Kynurenine
KYNU	Kynureninase
LPS	Lipopolysaccharide
OFT	Open field test
PKA	Protein kinase A
PVDF	Polyvinylidene fluoride
SSRIs	Selective Serotonin Reuptake Inhibitors
TDO	Tryptophan 2,3-dioxygenase
TLC	Thin-layer chromatography
TMS	Tetramethylsilane
TNF-α	Tumor necrosis factor-alpha
T_max_	Time to maximum concentration
TPH	Tryptophan hydroxylase
TRY	Tryptophan
TST	Tail suspension test
