# Supplementary material for: Design, Synthesis, and Biological Evaluation of Novel Morpholine–Coumarin Derivatives for Inflammation-Associated Depression
Source: Biomolecules. 2026 Jul 9;16(7):1002. doi: 10.3390/biom16071002 (PMC13406451; doi:10.3390/biom16071002)
Supplement: Supplementary file 1 [file biomolecules-16-01002-s001.zip › biomolecules-4393278-supplementary.pdf]

Design, Synthesis, and Biological Evaluation of Novel Morpholine–Coumarin Derivatives for Inflammation-Associated Depression

Hui Liu [1], Lina Hu [2], Yalan Wang [1], Zheshan Quan [1], Zheng Liu [3], Shiben Wang [2,*] and Qingkun Shen [1,*]

2.2. Pharmacology

2.2.1. In vitro IDO1 activity

IDO1 protein (50 nM) and the test compound were added to a reaction buffer (containing 50 mM potassium phosphate, pH 6.5, 20 mM ascorbic acid, 10 μM methylene blue, and 200 μg/mL catalase), followed by the addition of 400 μM L-tryptophan. The mixture was incubated at 37 °C for 60 min. The reaction was terminated by adding 40 μL of 30% (w/v) trichloroacetic acid to 200 μL of the total reaction system, followed by heating at 65 °C for 15 min. After cooling to 4 °C, the mixture was centrifuged at 1,125 × g for 10 min. An aliquot of 100 μL of the supernatant was transferred to a 96-well plate and mixed with 100 μL of 2% (w/v) p-dimethylaminobenzaldehyde (p-DMAB) prepared in acetic acid. The absorbance was measured at 490 nM using a microplate reader. All assays were performed in triplicate. IC_{50} values were calculated using GraphPad Prism software.

2.2.2. In vitro TDO activity

TDO protein (50 nM) and the test compound were added to a reaction buffer (containing 100 mM potassium phosphate, pH 6.5, 20 mM ascorbic acid, 20 μM methylene blue, and 200 μg/mL catalase), followed by the addition of 200 μM L-tryptophan. After mixing, the mixture was incubated at 37 °C for 75 min, and UV absorption was measured. The change in optical density at 321 nm was recorded. All assays were performed in triplicate. IC_{50} values were calculated using GraphPad Prism software.

2.2.3. Cell culture and treatment

BV2 cells were cultured in DMEM medium containing 10% fetal bovine serum and 1% penicillin/streptomycin in a humidified incubator with 5% CO_2 at 37 °C. Cells were seeded in six-well plates at a density of 1.0×10^6 cells per well. When cell confluence reached 60-70%, cells were pretreated with different concentrations of the compound for 1 h, followed by the addition of 100 ng/mL LPS and further incubation for 24 h.

2.2.4. Detection of cytokine mRNA expression

Quantitative reverse transcription polymerase chain reaction (qRT-PCR) was used to analyze the mRNA transcription levels of cytokines. Total RNA was extracted using TRIzol, and its concentration and purity were determined using a microspectrophotometer. Subsequently, RNA samples were reverse transcribed into cDNA using the Takara PrimeScript™ RT Reagent Kit. qRT-PCR analysis was performed using 2× Realab Green PCR Fast Mixture (Universal) and a real-time PCR system (QIAGEN, Malaysia). Primers were synthesized by Sangon Biotech, and details are provided in Supplementary **Table S1**.

2.2.5. Western blot analysis

Proteins were extracted and separated by 10-12.5% SDS-PAGE, then transferred to PVDF membranes (Millipore). After blocking with skim milk, the membranes were incubated overnight at 4 °C with primary antibodies (anti-IDO1, 1:1000; anti-TDO, 1:1000, Proteintech; anti-PKA, 1:500; anti-BDNF, 1:500; anti-β-tubulin, 1:2000; anti-GAPDH, 1:2000, Servicebio). Subsequently, the membranes were incubated with HRP-conjugated secondary antibodies (1:10000, Abways) for 2 h at room temperature. Protein bands were visualized using a chemiluminescence imaging system (Shanghai Jiabao) and quantified using Image J software.

2.2.6. Safety evaluation

C57BL/6 Mice (a total of forty male C57BL/6J mice, 6–8 weeks old, 20–22 g) were randomly divided into four groups (n = 10 per group) and treated intraperitoneally once daily for 14 days at three different doses: 10, 20, and 30 mg/kg. The control group received an equal volume of the vehicle. Body weights were recorded daily, and mice were sacrificed on day 14 (At the end of the experiment, humane euthanasia was performed by cervical dislocation to minimize animal suffering). Pathological changes in the heart, liver, spleen, lung, and kidney were assessed by H&E staining.

2.2.7. Pharmacokinetic study

Preparation of drug-containing plasma: Eighteen male C57BL/6 mice (6-8 weeks old, 20-22 g) were adaptively fed for 3 days and then randomly divided into an intravenous injection group and an intraperitoneal injection group (a total of 18 mice. n = 9 per group). The compound was administered at a dose of 20 mg/kg. Blood samples (0.1-0.2 mL) were collected from the tail vein at 0.25, 0.5, 1, 2, 4, 6, 12, 24, 48, and 72 h post-administration into heparin sodium tubes, centrifuged at 4000 rpm for 15 min at 4 °C, and the upper plasma layer was collected and stored at -80 °C.

Sample pretreatment: An aliquot of 100 μL of plasma was mixed with 5 μL of internal standard (containing 50 % formic acid) and vortexed. Then, 300 μL of ethyl acetate was added, vortexed for 2 min, and ultrasonicated for 10 min. After centrifugation at 12,000 rpm for 10 min at 4 °C, the supernatant was collected and evaporated to dryness under nitrogen. The residue was reconstituted with 50 μL of methanol:acetonitrile:water (4:4:2), vortexed for 2 min, centrifuged under the same conditions, and the supernatant was analyzed. Quality control (QC) samples were prepared by mixing 90 μL of blank plasma with 10 μL of working solution and processed using the same method.

Data analysis: LC-MS/MS data were acquired using Analyst software, and pharmacokinetic parameters, including $AUC_{0\text{-}t}$, $AUC_{0\text{-}\infty}$(trapezoidal method), $T_{1/2}$, C_{max}, and T_{max}, were calculated using Phoenix WinNonlin 8.2.

2.2.8. In vivo behavioral experiments

All C57BL/6 mice (a total of thirty male mice, n = 10 per group) were housed in an SPF-grade laboratory with free access to food and water. The housing environment was maintained at a constant temperature of 22 ± 2 °C with a 12 h dark/12 h light reversed light cycle. Acute depressive-like responses were induced using lipopolysaccharide (LPS, 2 mg/kg, Solarbio). The compound was administered intraperitoneally at a dose of 20 mg/kg/day.

Administration was initiated two days before or on the day of LPS injection and continued daily until the end of the experiment according to the same schedule.

Forced swim test: A cylindrical glass tank (20 cm in height, 15 cm in diameter) was filled with an appropriate volume of water to prevent the mice from escaping or supporting themselves against the tank walls, with the water temperature maintained at 22 ± 2 °C. The DigBehv animal behavior video analysis system was used for video tracking and behavioral data recording. When the animal behavior analysis software determined that the mice was making active movements, it was considered struggling; otherwise, it was considered immobile. The total duration of immobility was recorded during the last 4 min of the 6-minute test period.

Tail suspension test: Each mice was individually suspended in a three-walled rectangular compartment (55 cm × 35 cm × 35 cm). The mice was fixed with adhesive tape approximately 2 cm from the tip of the tail and suspended in the center of the compartment, preventing escape or support against the walls or floor. The test lasted for 6 min. During the observation period, an observer unaware of the treatment status recorded whether each mice was immobile or struggling. The duration of immobility was calculated during the last 4 min of the 6-minute period.

Open field test: Each mice was placed individually in a plastic box (50 cm × 40 cm × 40 cm). The test lasted for 6 min, and the number of rearing events, grooming behaviors, and crossings were recorded during the last 4 min of the experiment.

After behavioral assessments, all mice were anesthetized, and blood was collected from the abdominal aorta. Tissues were harvested for immunofluorescence analysis and H&E staining.

2.2.9. ELISA assay

Brain tissue was homogenized in PBS (0.01 M, pH = 7.4, containing protease inhibitors) at 4 °C, centrifuged at 5,000 rpm for 10 min at 4 °C, and the supernatant was collected. Levels of 5-HT and kynurenine in brain tissue and serum were measured by ELISA according to the manufacturer’s instructions (Boyan Biotechnology).

2.2.10. Histopathology and immunofluorescence staining

Histopathological analysis and immunofluorescence detection were performed by Wuhan Servicebio Biotechnology Co., Ltd. For histopathological analysis, mice tissues were immediately fixed in 4% paraformaldehyde (PFA), embedded in paraffin, sectioned, and stained with hematoxylin and eosin (H&E). For immunofluorescence detection, sections were blocked with 3% bovine serum albumin (BSA) in phosphate-buffered saline (PBS) for 30 min at room temperature, then incubated with primary antibodies diluted appropriately in PBS overnight at 4 °C. After washing three times with PBS, slides were incubated with fluorescently labeled secondary antibodies (matched to the primary antibody species) for 50 min at room temperature in the dark. Following three additional washes with PBS, slides were incubated with 4′,6-diamidino-2-phenylindole (DAPI) solution for 5 min at room temperature in the dark. Immunofluorescence images were scanned using a Servicebio fluorescence scanner. Data were analyzed using Image J and the Servicebio browsing analysis software Saiviewer.

2.2.11. Molecular docking study

In this experiment, compound **14d** was selected for molecular docking studies. X-ray structures of1 IDO1 (PDB ID: 5WHR) and TDO (PDB ID: 6PYZ) used for molecular docking were obtained from the RCSB PDB protein database (https://www.rcsb.org/). Molecular docking was performed and analyzed using DS 2021 software, specifically its molecular docking module (CDOKER). For IDO1, a spherical area defined by coordinates x = 11.214, y = 30.489, z = 32.070 with a radius of 11.0 was selected for molecular docking studies. For TDO, a spherical area defined by coordinates x = 39.349, y = -62.288, z = -31.217 with a radius of 10.0 was selected for molecular docking studies.

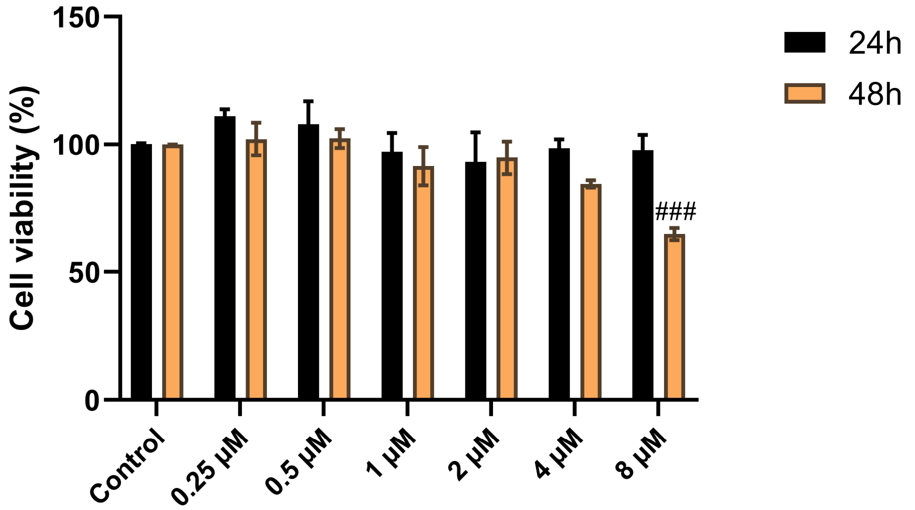

Figure S1. To evaluate the cytotoxicity of compound **14d**, six concentrations (0.25, 0.5, 1, 2, 4, and 8 μM) were tested. Data are expressed as mean ± SEM; $^{***}p < 0.001$ vs. control group.

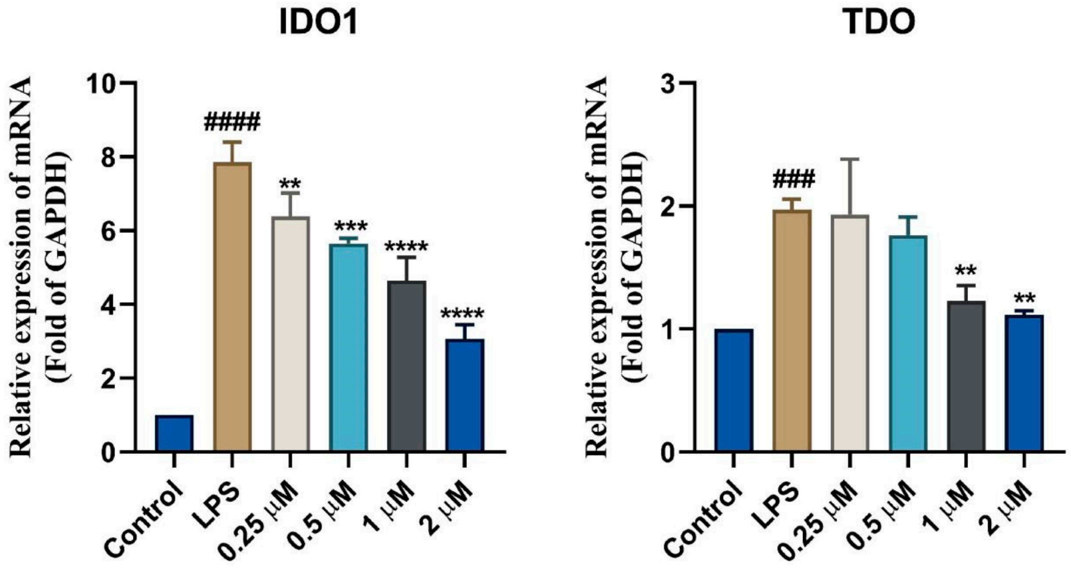

Figure S2. Compound **14d** exerts anti-inflammatory effects by downregulating the mRNA transcription levels of IDO1 and TDO *in vitro* LPS-induced microglial inflammation model. Data are expressed as mean ± SEM; $^{###}p < 0.001$, $^{####}p < 0.0001$ vs. control group; $^{**}p < 0.01$, $^{***}p < 0.001$, $^{****}p < 0.0001$ vs. LPS

group.

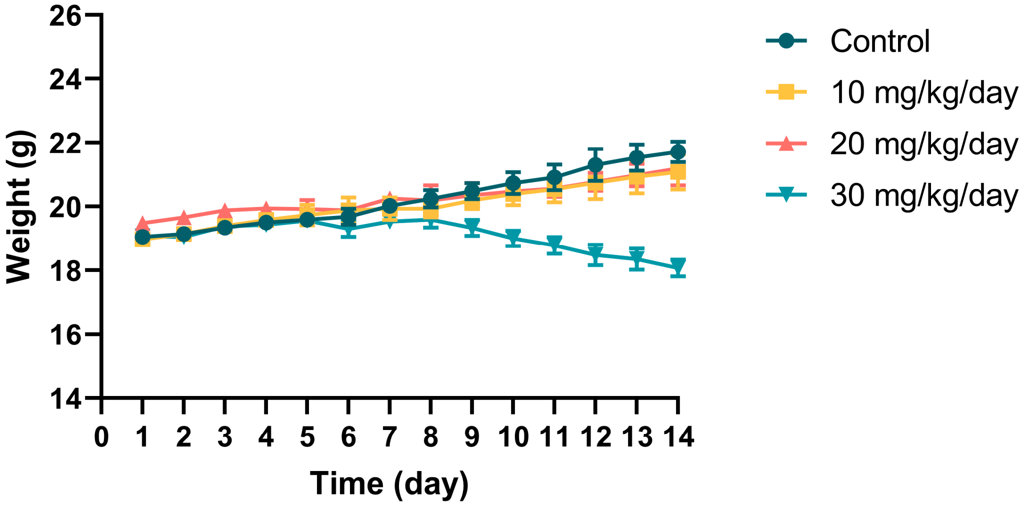

Figure S3. Daily body weight changes in mice after 14 days of compound **14d** administration.

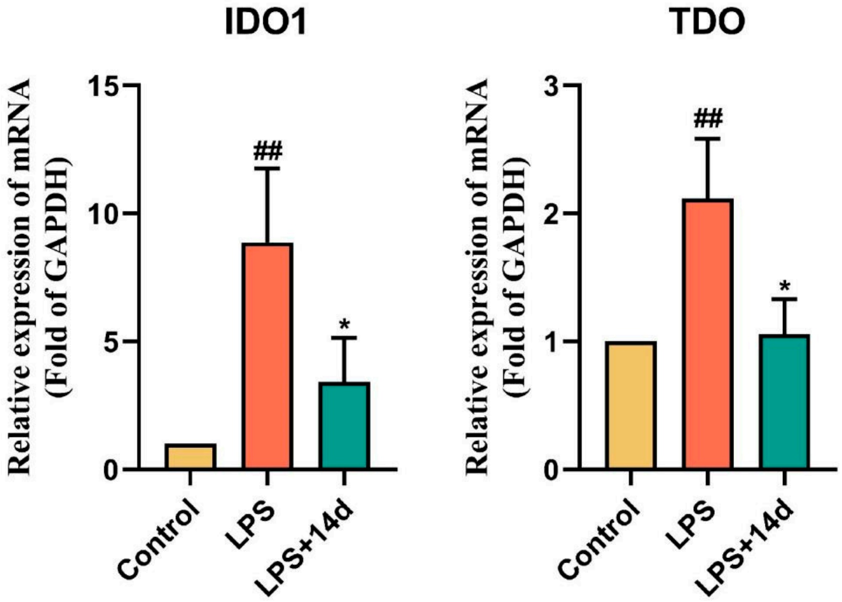

Figure S4. Compound **14d** downregulates the mRNA expression levels of IDO1 and TDO in mice brain tissue, with n = 8-10 mice per group. Data are expressed as mean ± SEM; ##$p < 0.01$ vs. control group; *$p < 0.05$ vs. LPS group.

Table S1. Primer sequences used in qRT-PCR.

Primer	Forward	Reverse
IL-1β	5′-CCC AAG CAA TAC CCA AAG AA-3′	5′-GCT TGT GCT CTG CTT GTG AG-3′
COX2	5′-TCA TTG GTG GAG AGG TGT AT-3′	5′-ACC CCA CTC AGG ATG CTC CT-3′
iNOS	5′-GTG TTC TTT GCT TCC ATG CT-3′	5′-AGT TGC TCC TCT TCC AAG GT-3′
TNF-α	5′-GAG TGA CAA GCC TGT AGC CCA-3′	5′-AGC TCC ACG CCA TTG GC-3′
IL-10	5′-CTA GAG CTG CGG ACT GCC TTC-3′	5′-TTG ATT TCT GGG CCA TGC-3′
IDO1	5′-CCAGTGCAGTAGAGCGTCAA-3′	5′-TCCCAGACCCCCTCATACAG-3′
TDO	5′-ACGACGACTGTCATACCGTG -3′	5′-ACCTTGTACCTGTCGCTCAC-3′
PKA	5′-TCCAGCTCCAACGATGTGAAA-3′	5′-TTCATGGCGTAGTGGTTCCC-3′
GAPDH	5′-TGA ATA CGG CTA CAG CAA CA-3′	5′-AGG CCC CTC CTG TTA TTA TG-3′

^{1}H-NMR, ^{13}C-NMR and HRMS spectra of the target compound:

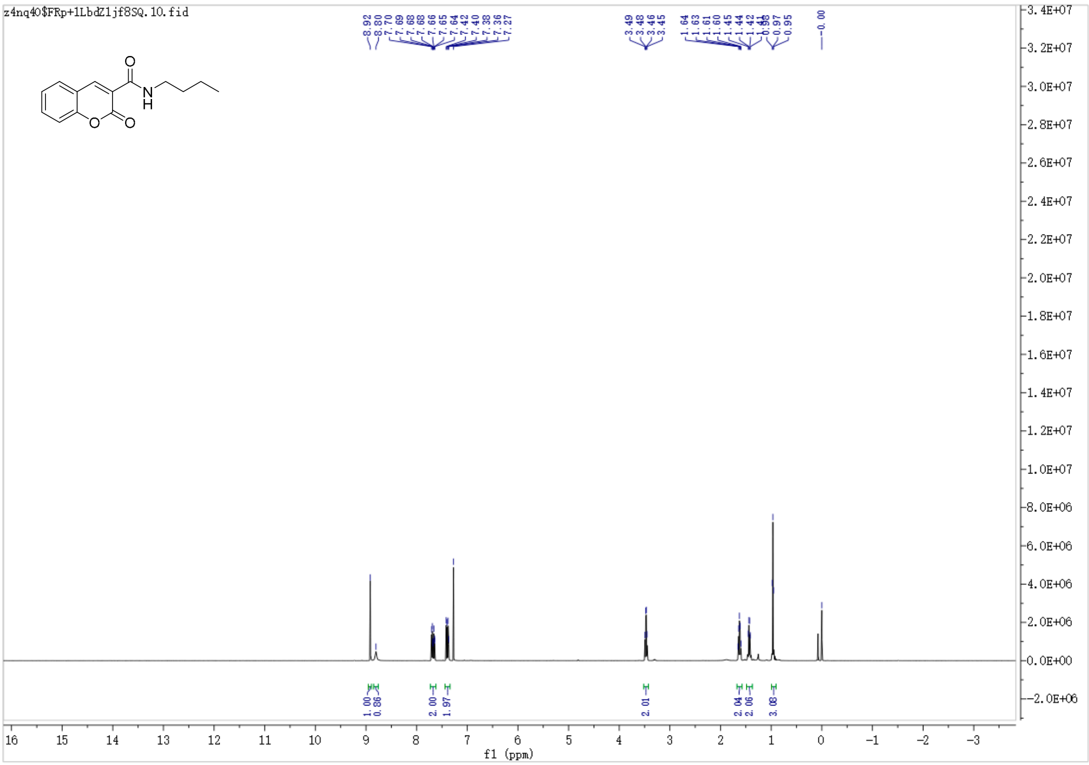

Figure S5. ^{1}H-NMR ($CDCl_3$, 500 MHz, ppm) spectrum of compound **3a**.

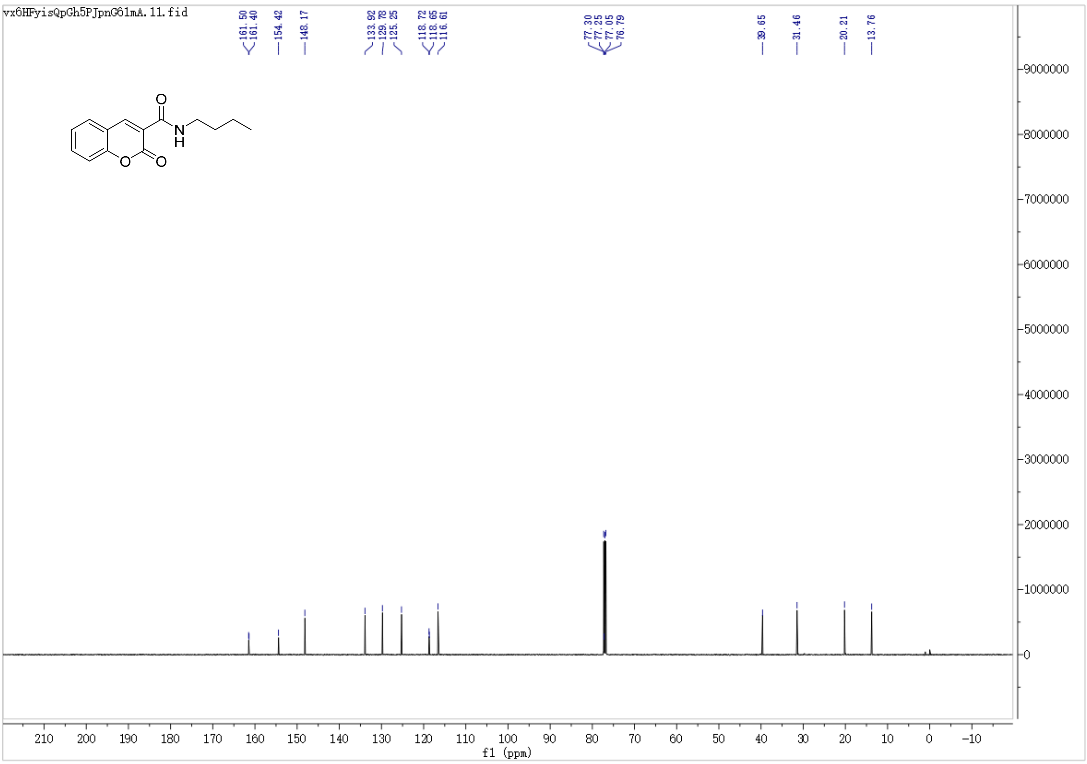

Figure S6. ^{13}C-NMR ($CDCl_3$, 126 MHz, ppm) spectrum of compound **3a**.

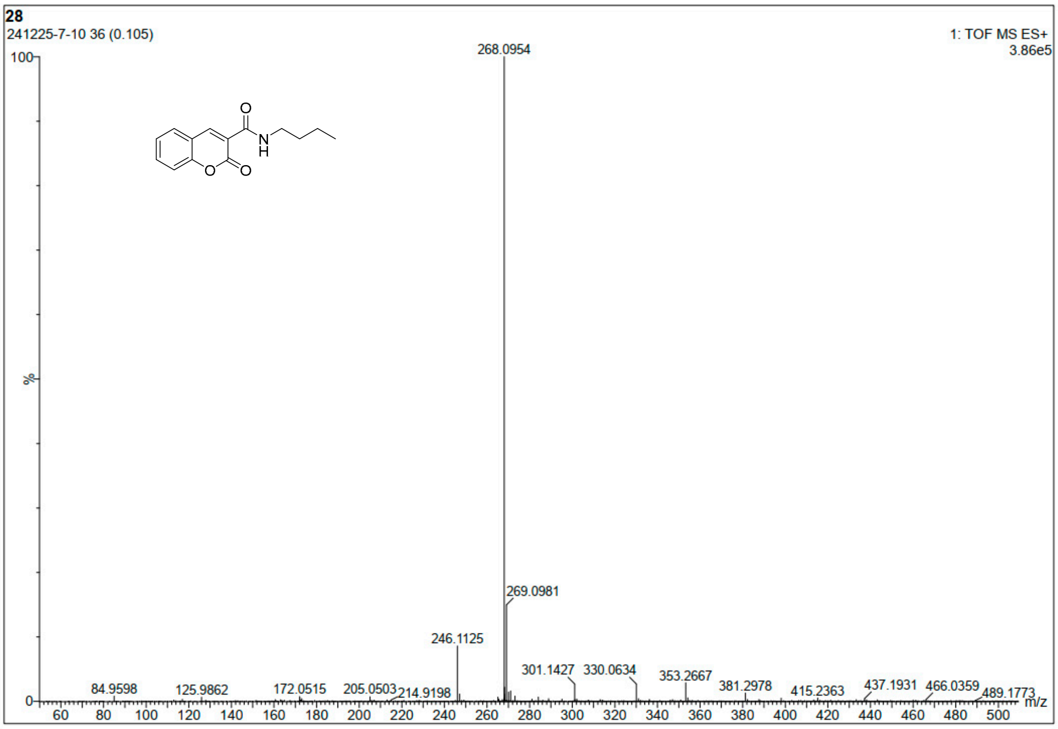

Figure S7. HRMS ($[M+Na]^+$) spectrum of compound **3a**.

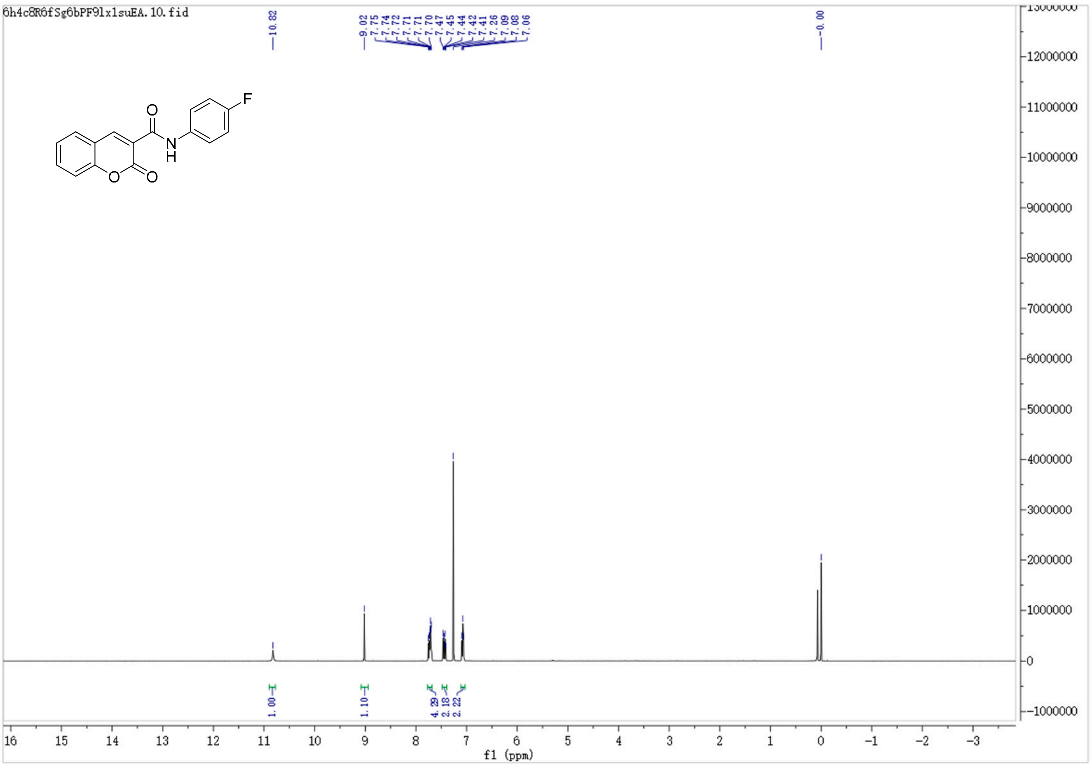

Figure S8. ^{1}H-NMR ($CDCl_3$, 500 MHz, ppm) spectrum of compound **3b**.

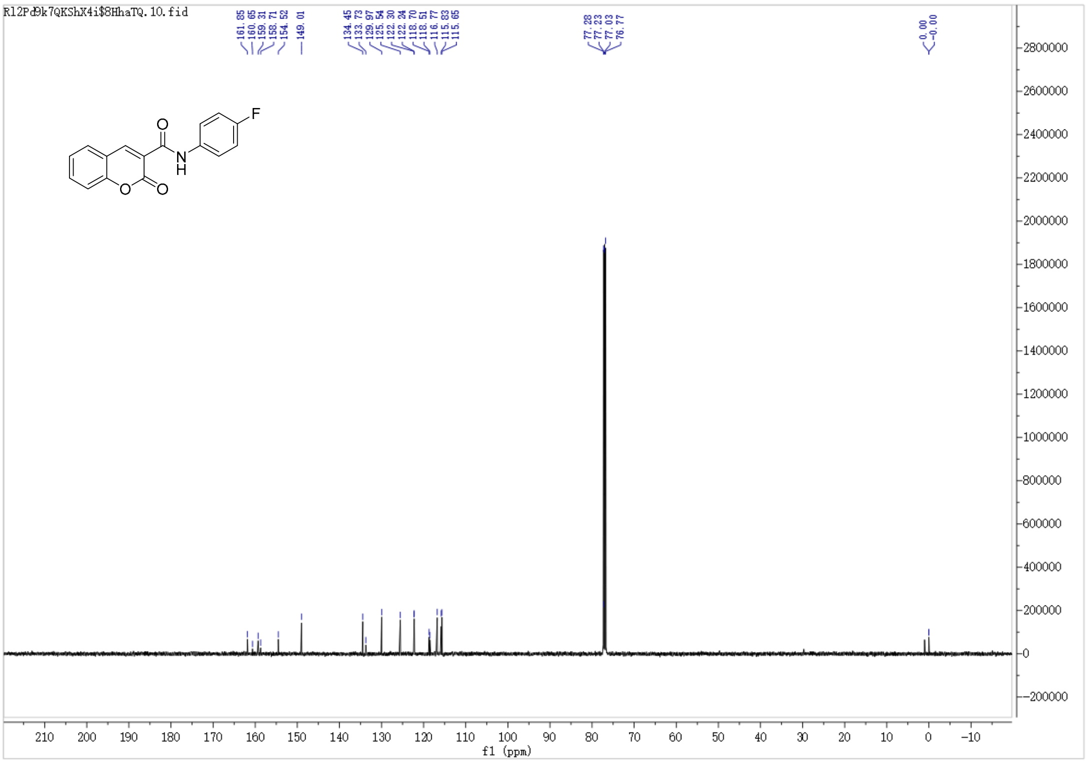

Figure S9. ^{13}C-NMR ($CDCl_3$, 126 MHz, ppm) spectrum of compound **3b**.

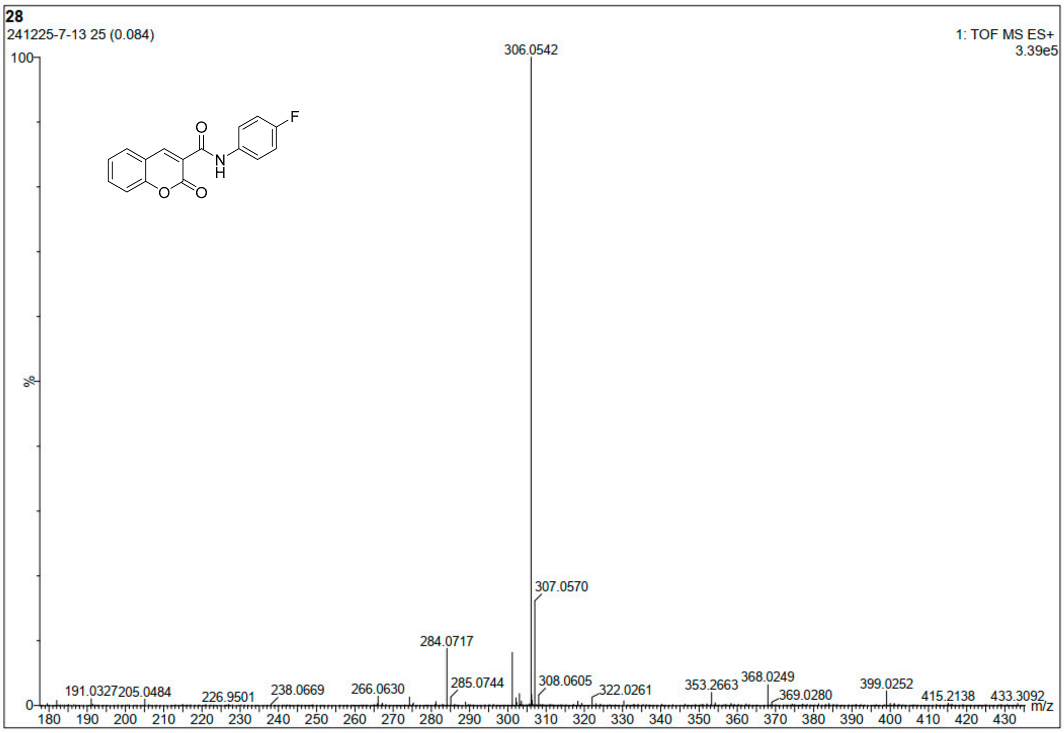

Figure S10. HRMS ([M+Na]$^{+}$) spectrum of compound **3b**.

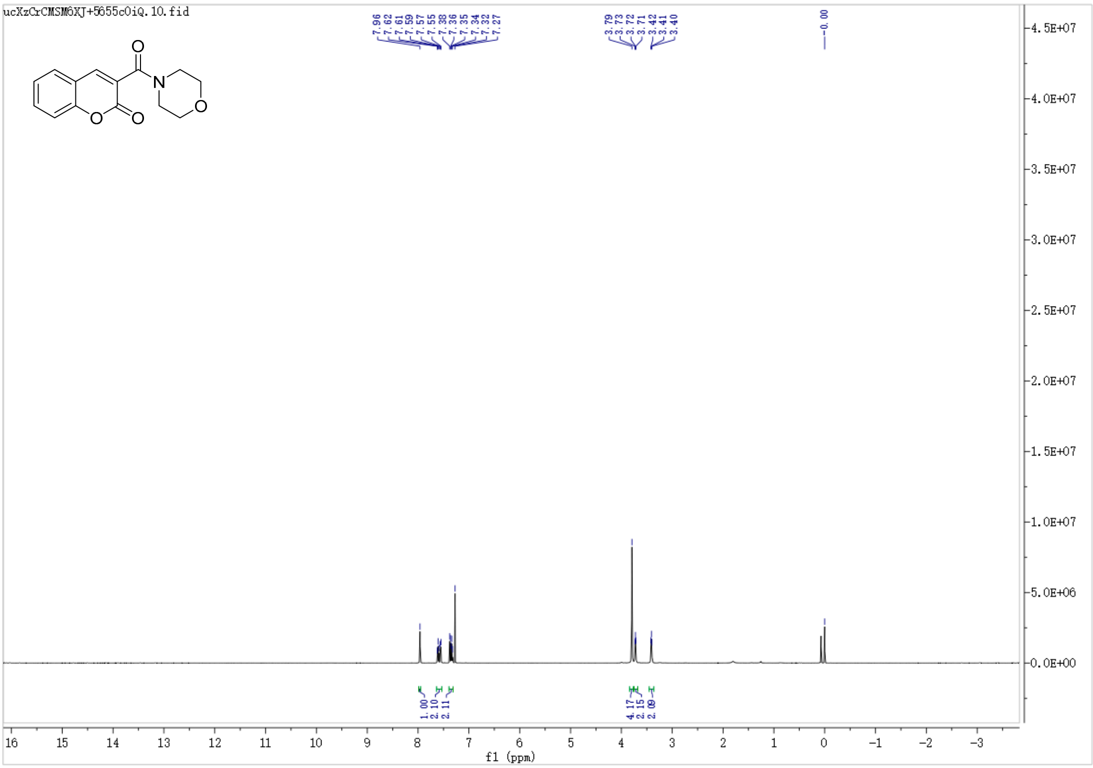

Figure S11. ^{1}H-NMR (CDCl3, 500 MHz, ppm) spectrum of compound **3c**.

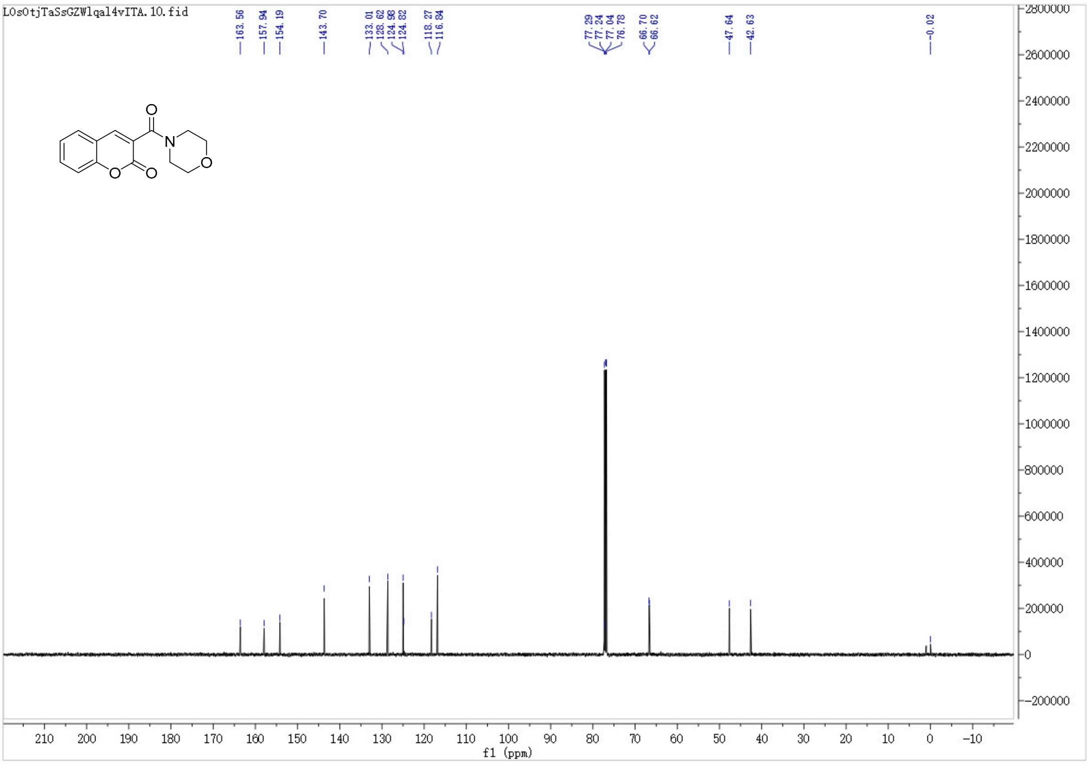

Figure S12. ^{13}C-NMR ($CDCl_3$, 126 MHz, ppm) spectrum of compound **3c**.

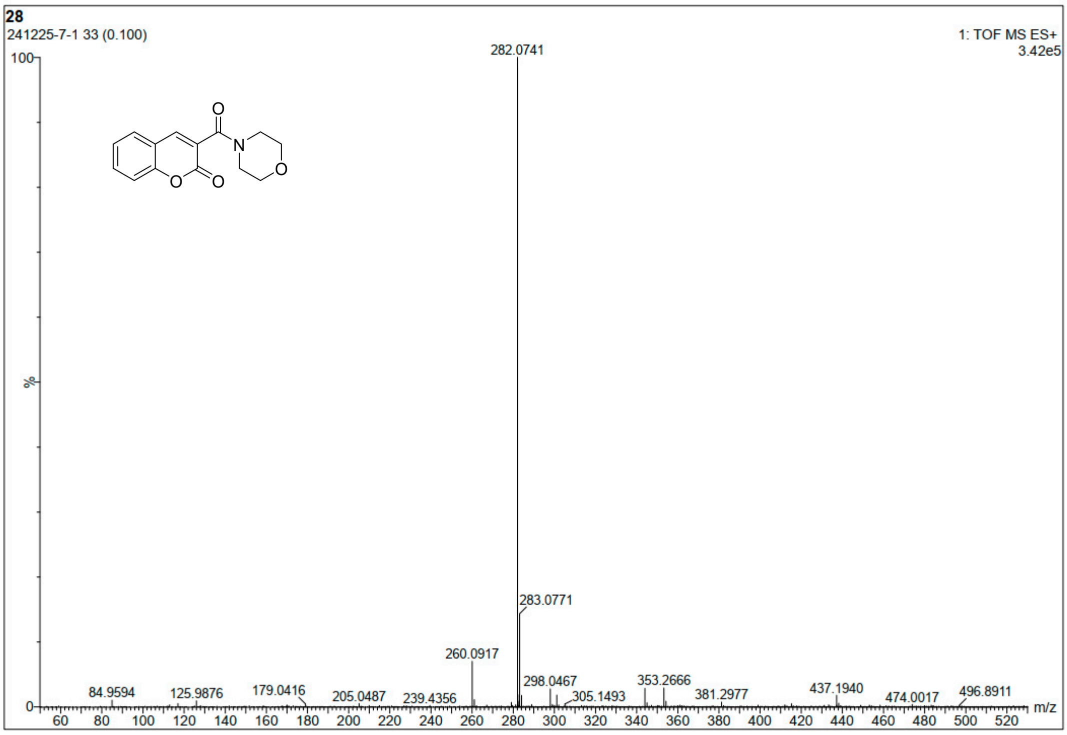

Figure S13. HRMS ([M+Na]$^+$) spectrum of compound **3c**.

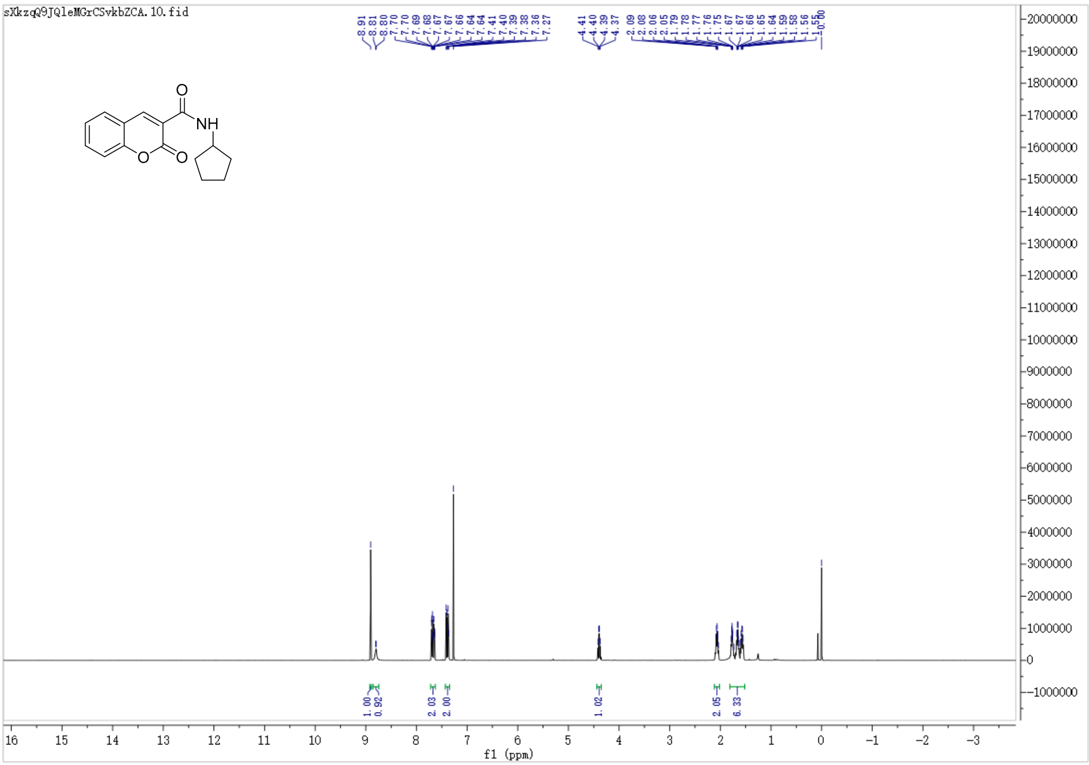

Figure S14. ^{1}H-NMR ($CDCl_3$, 500 MHz, ppm) spectrum of compound **3d**.

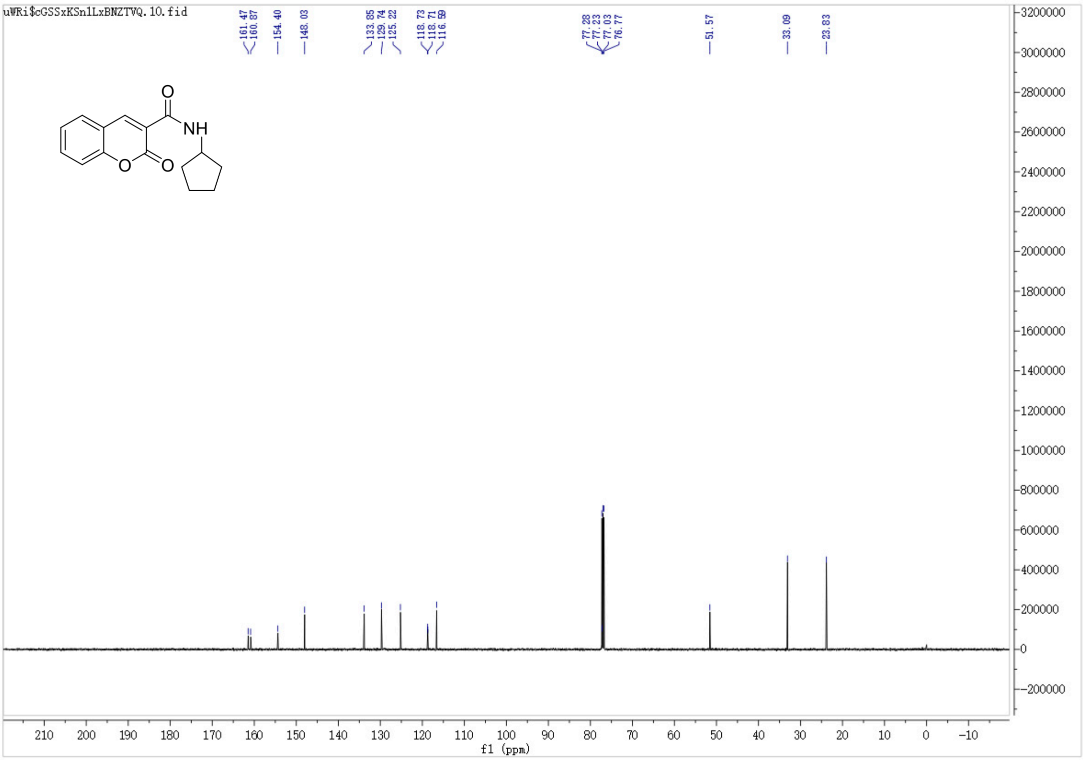

Figure S15. ^{13}C-NMR ($CDCl_3$, 126 MHz, ppm) spectrum of compound **3d**.

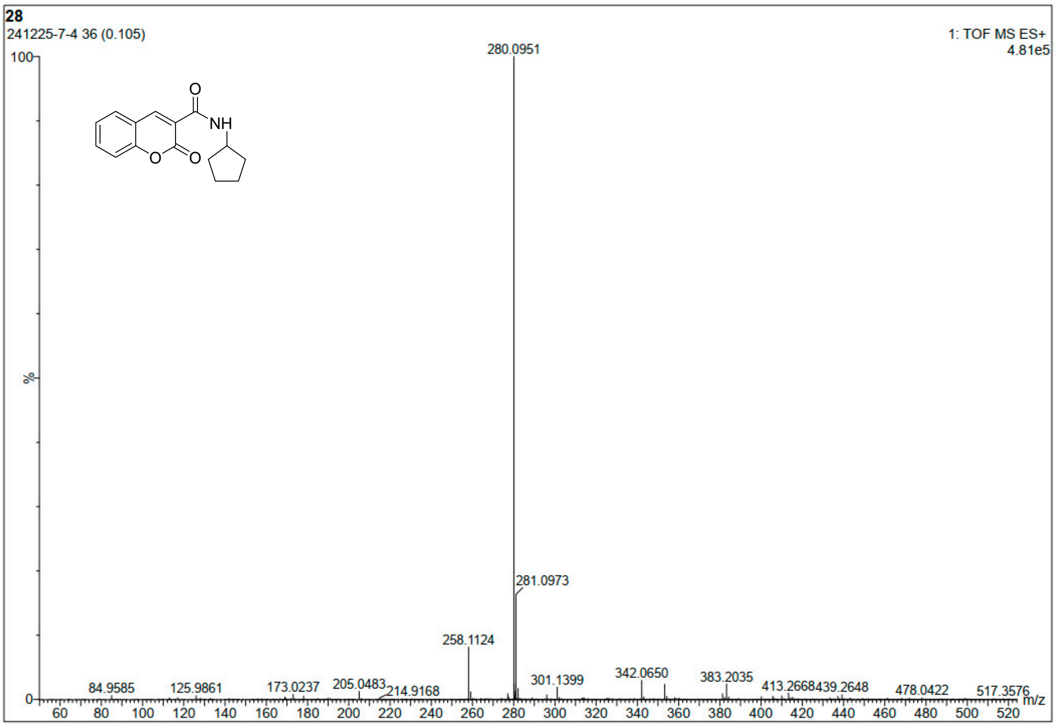

Figure S16. HRMS ([M+Na]$^+$) spectrum of compound **3d**.

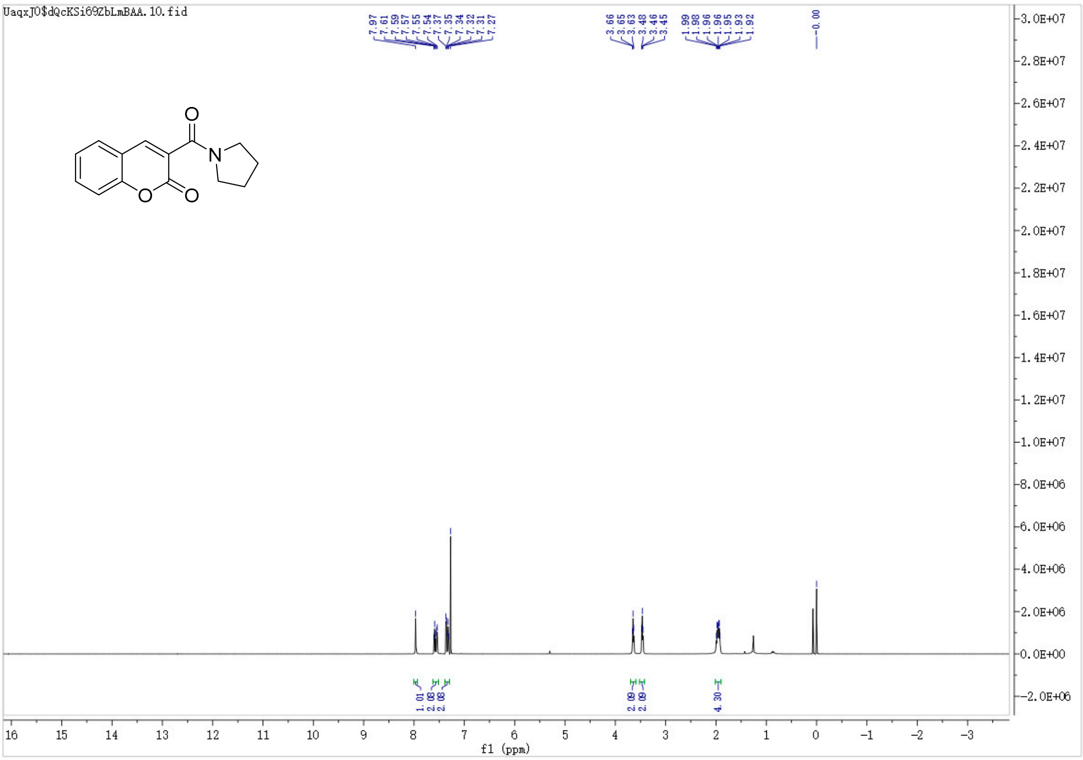

Figure S17. ^{1}H-NMR ($CDCl_3$, 500 MHz, ppm) spectrum of compound **3e**.

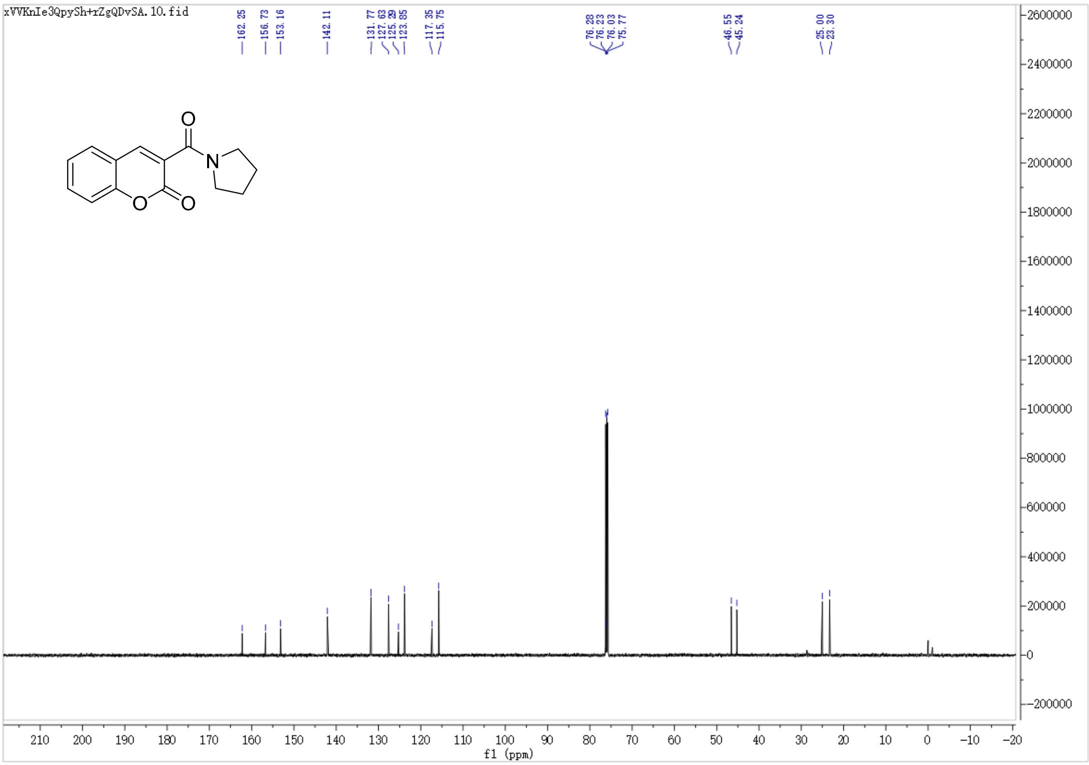

Figure S18. ^{13}C-NMR ($CDCl_3$, 126 MHz, ppm) spectrum of compound **3e**.

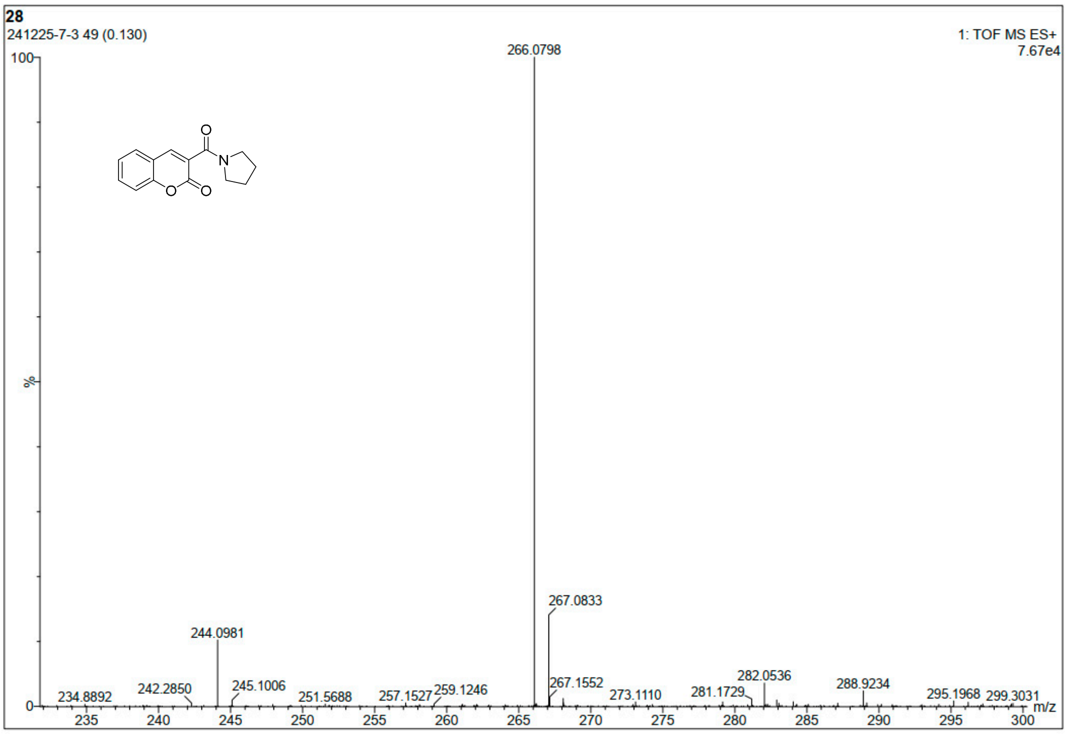

Figure S19. HRMS ([M+Na]$^{+}$) spectrum of compound **3e**.

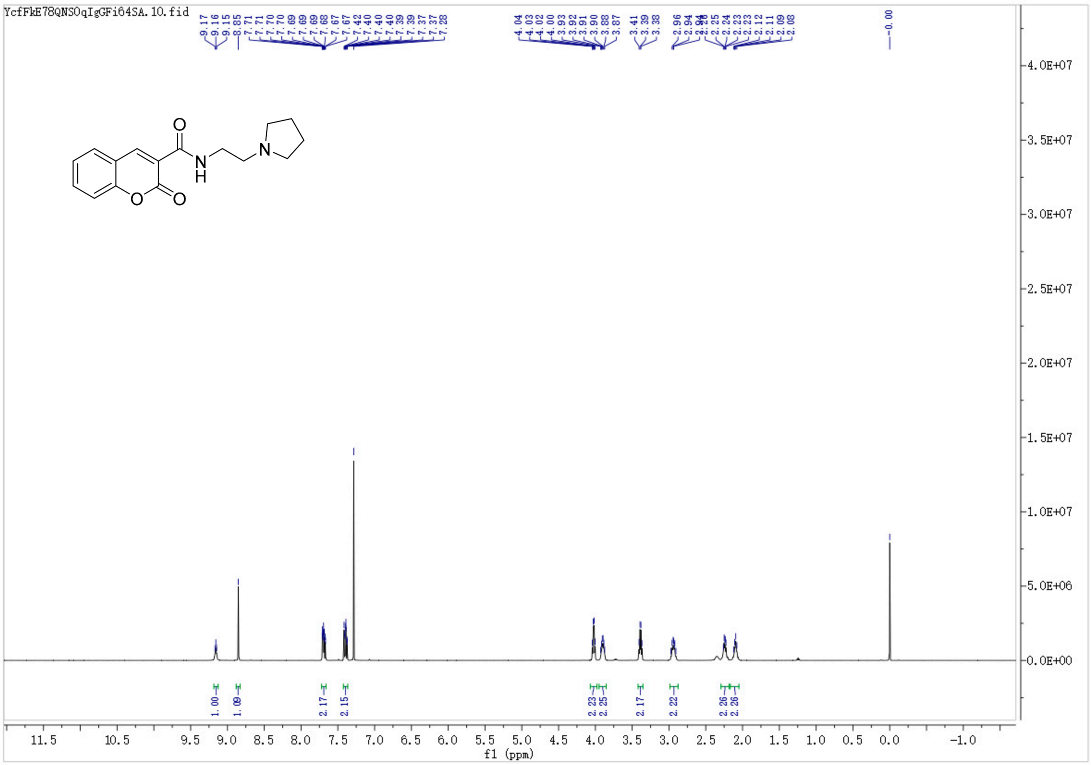

Figure S20. ^{1}H-NMR ($CDCl_3$, 500 MHz, ppm) spectrum of compound **3f**.

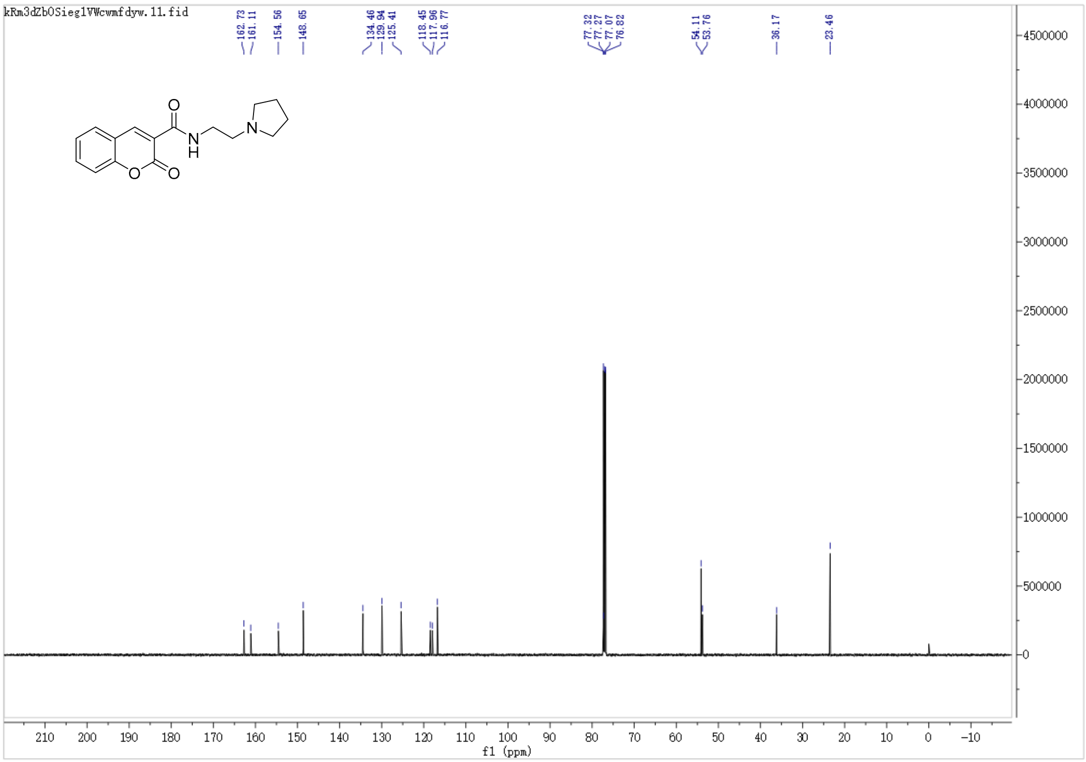

Figure S21. ^{13}C-NMR ($CDCl_3$, 126 MHz, ppm) spectrum of compound **3f**.

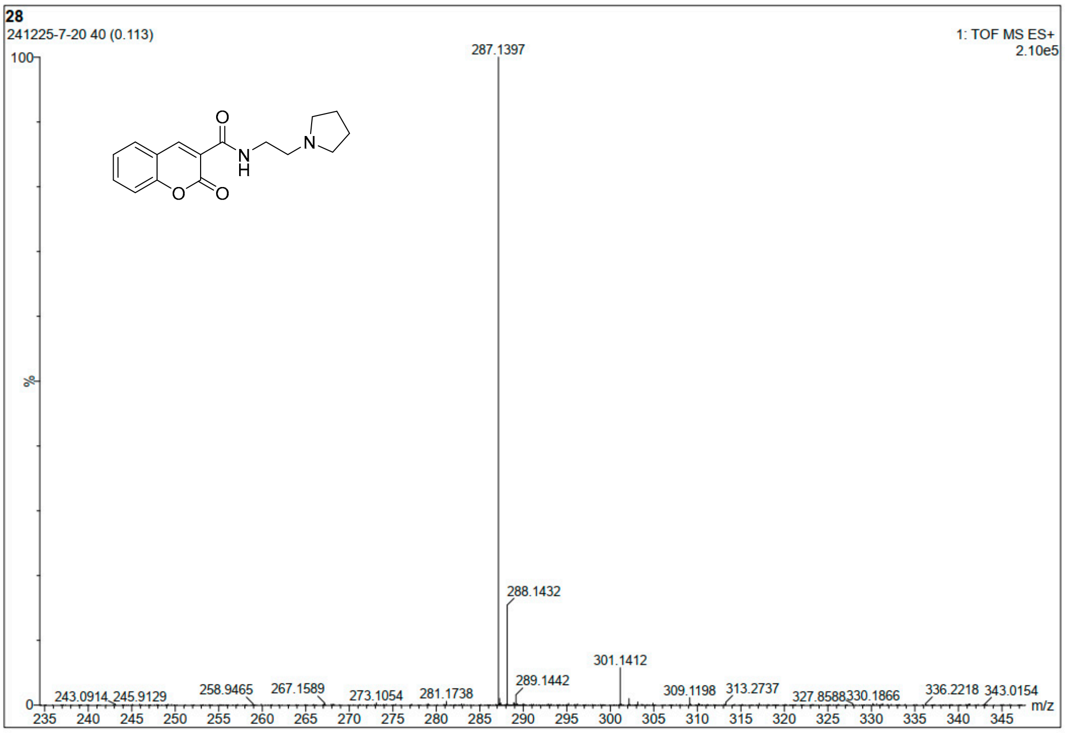

Figure S22. HRMS ([M+H]$^{+}$) spectrum of compound **3f**.

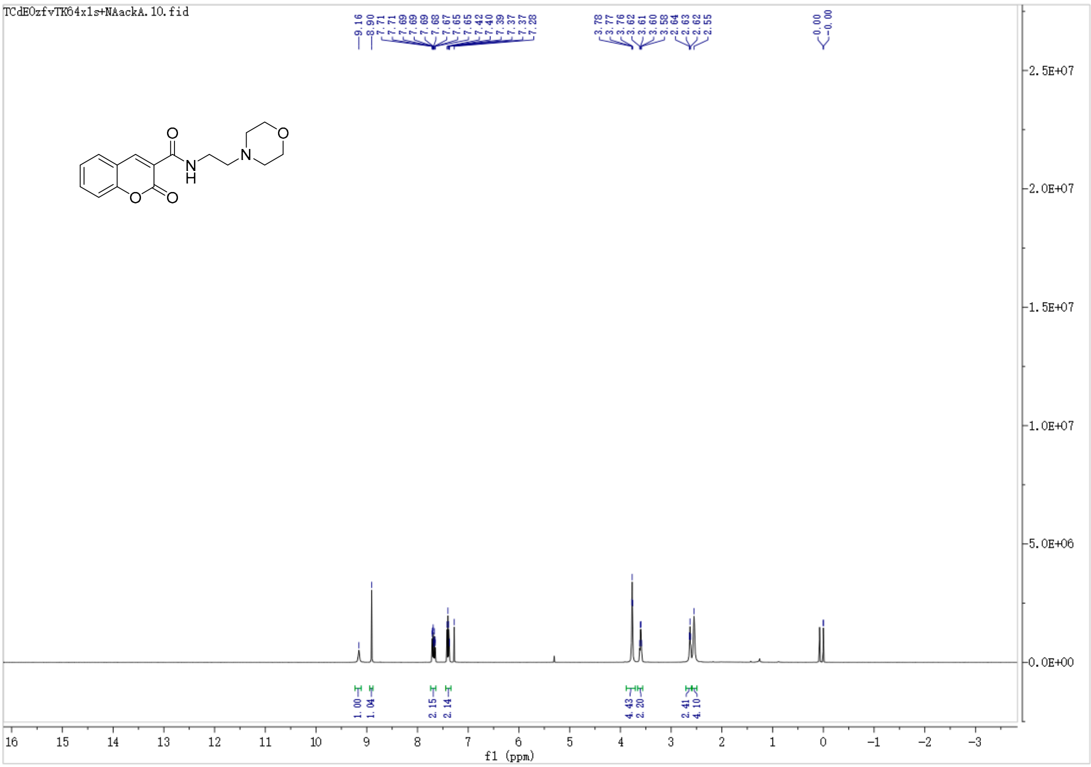

Figure S23. ^{1}H-NMR ($CDCl_3$, 500 MHz, ppm) spectrum of compound **3g**.

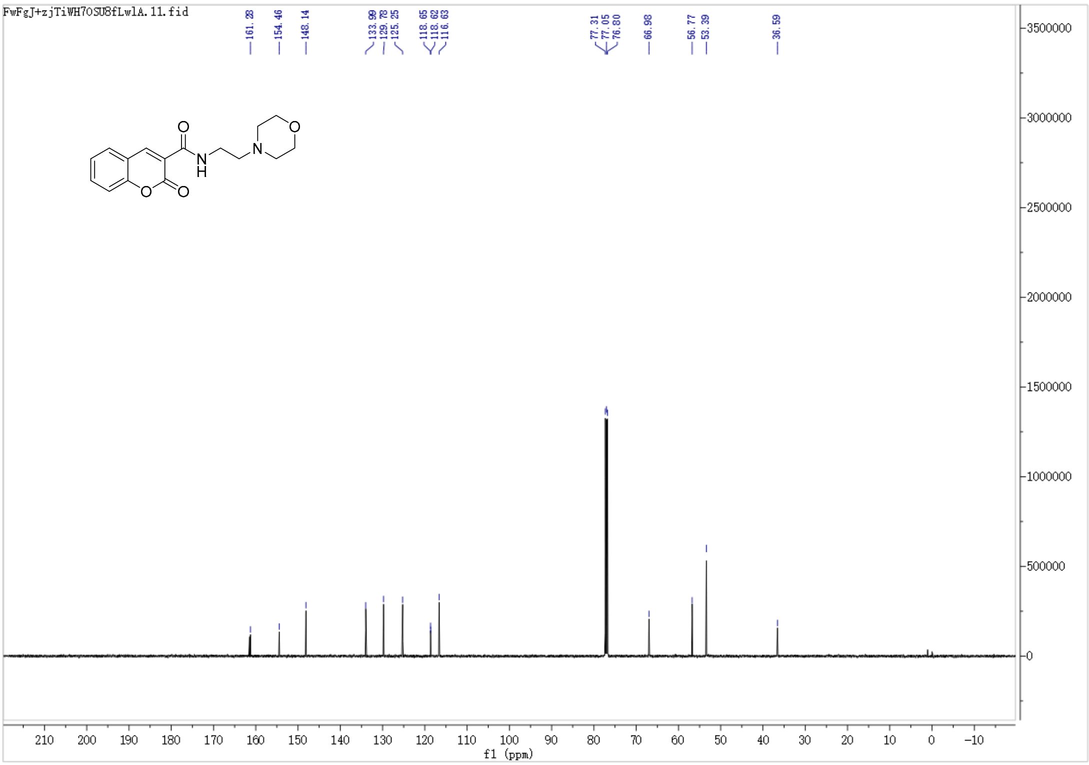

Figure S24. ^{13}C-NMR ($CDCl_3$, 126 MHz, ppm) spectrum of compound **3g**.

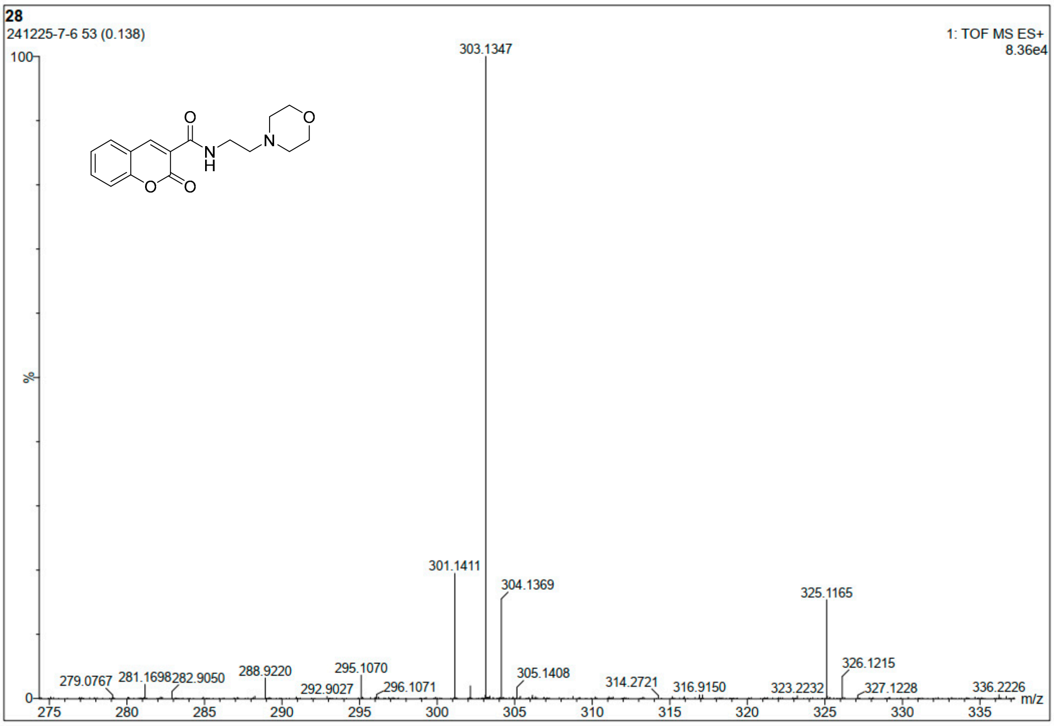

Figure S25. HRMS ($[M+H]^+$) spectrum of compound **3g**.

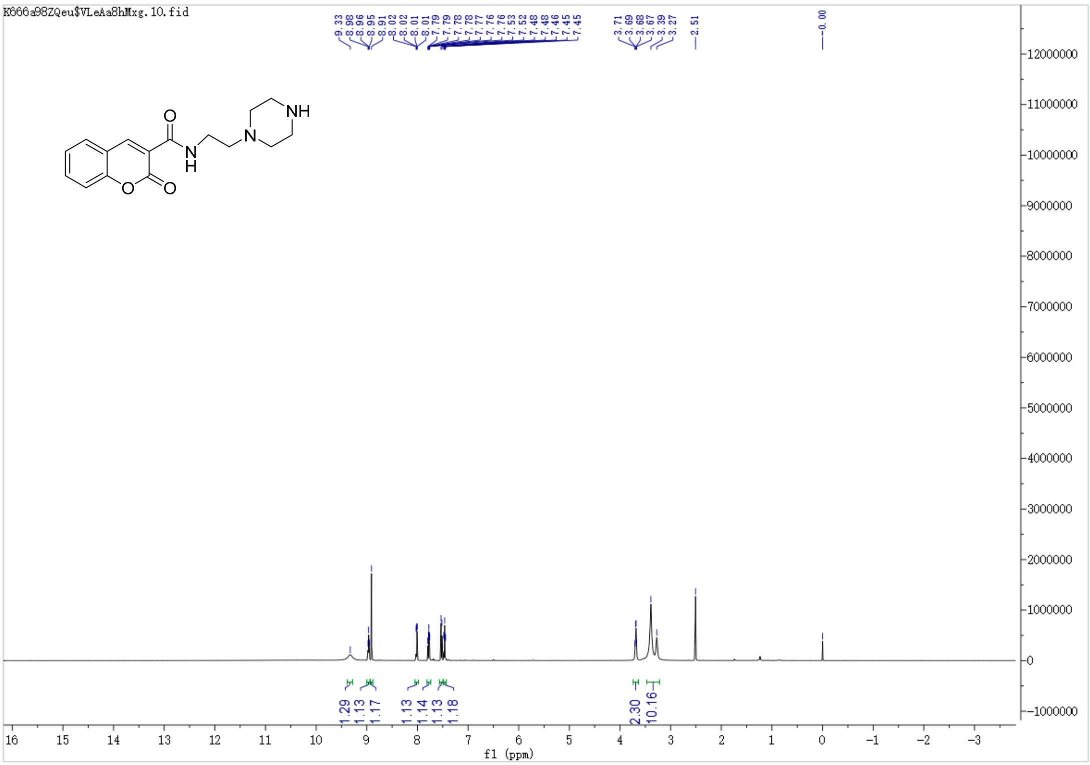

Figure S26. ^{1}H-NMR (DMSO$_{d6}$, 500 MHz, ppm) spectrum of compound **5**.

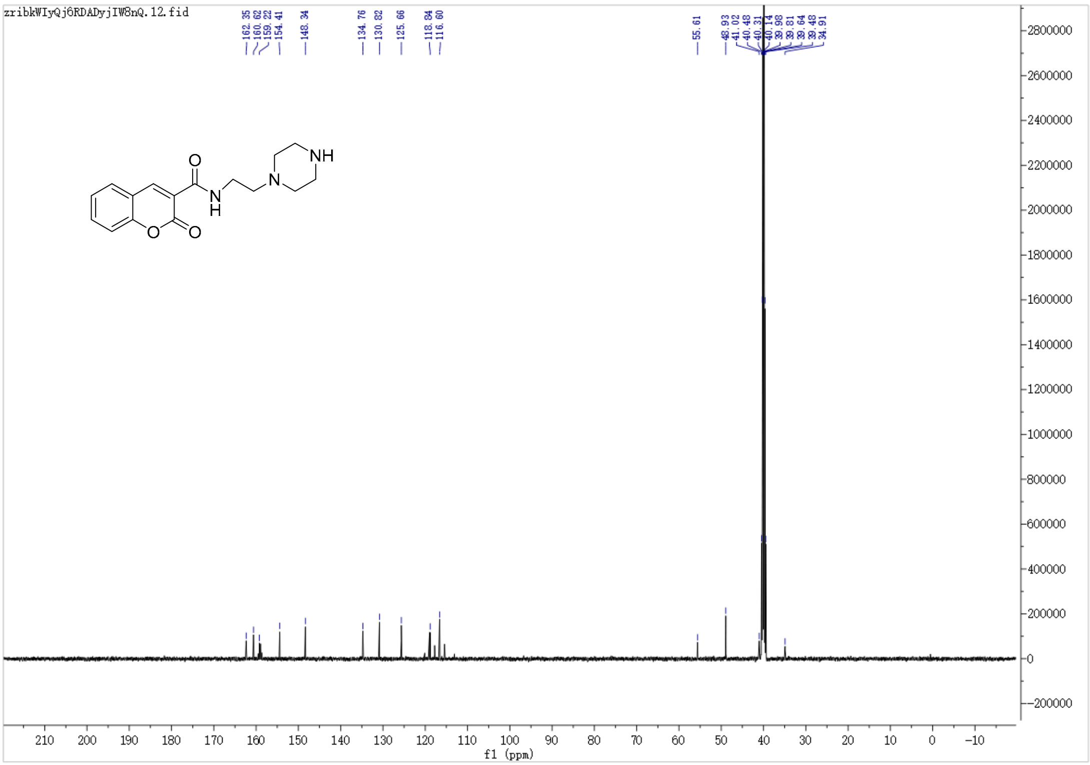

Figure S27. ^{13}C-NMR (DMSO$_{d6}$, 126 MHz, ppm) spectrum of compound **5**.

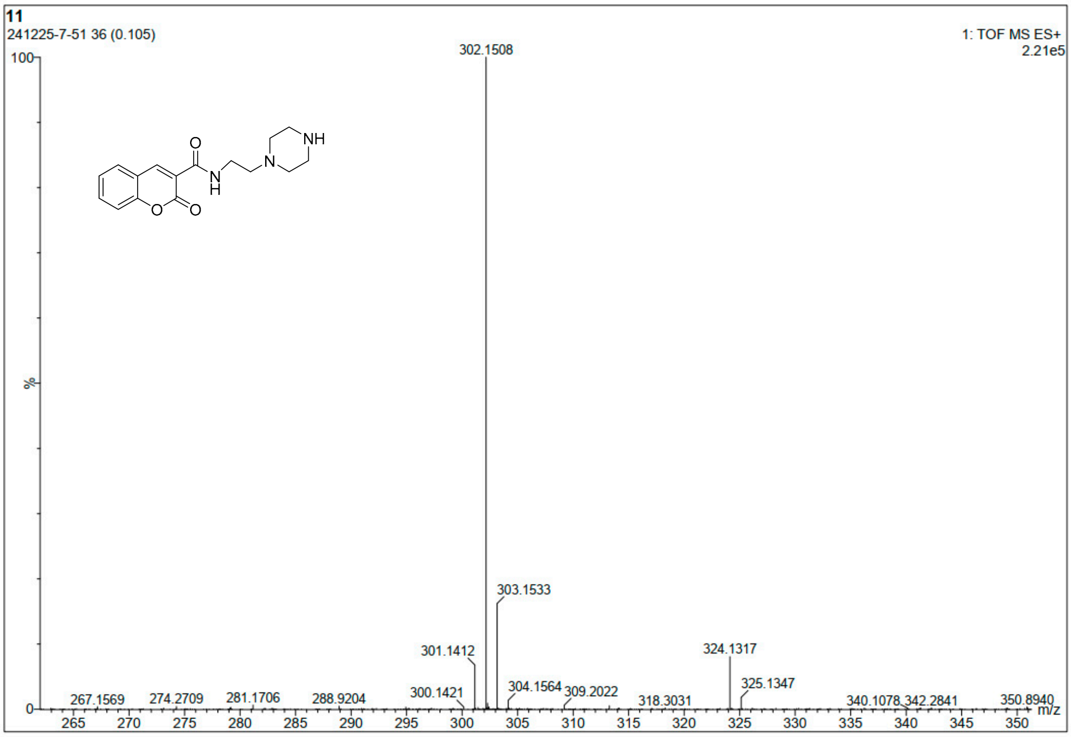

Figure S28. HRMS ([M+H]$^+$) spectrum of compound **5**.

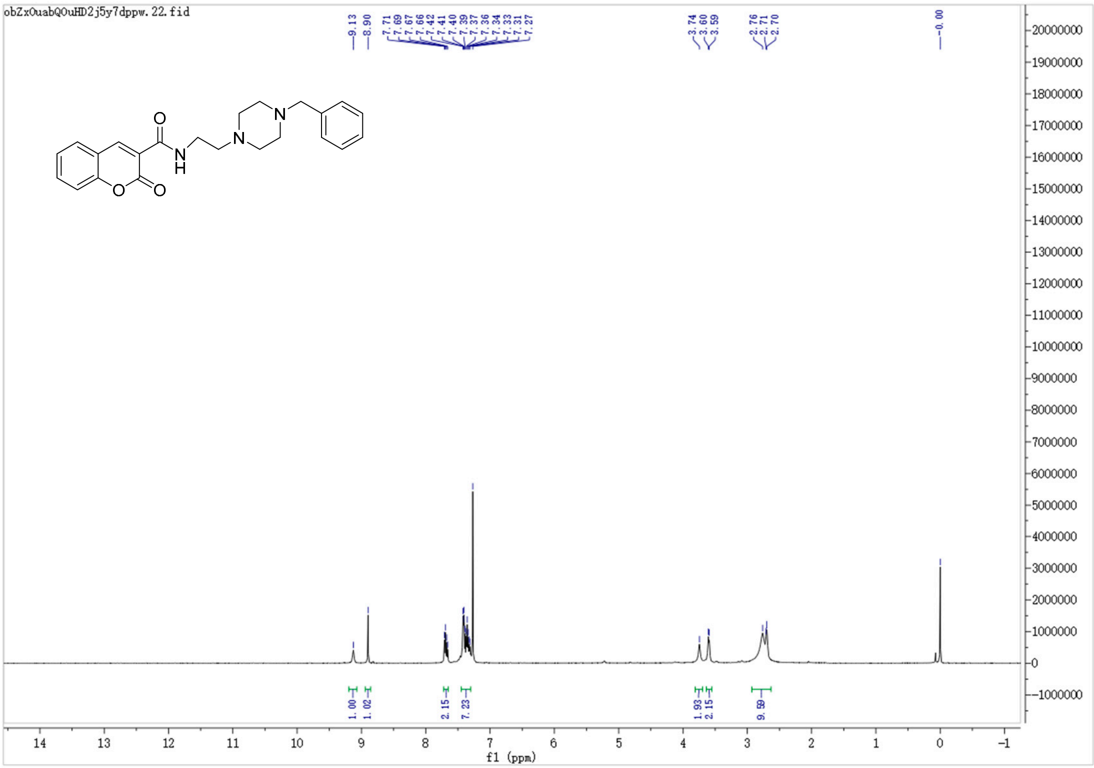

Figure S29. ^{1}H-NMR ($CDCl_3$, 500 MHz, ppm) spectrum of compound **6**.

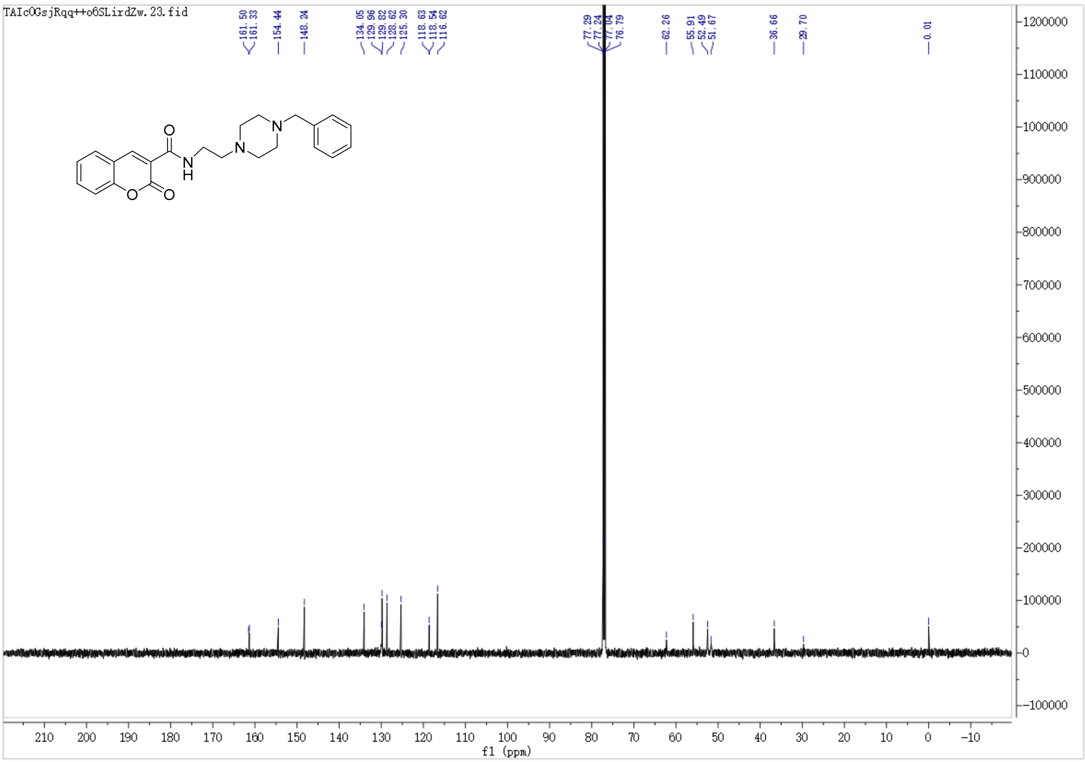

Figure S30. ^{13}C-NMR ($CDCl_3$, 126 MHz, ppm) spectrum of compound **6**.

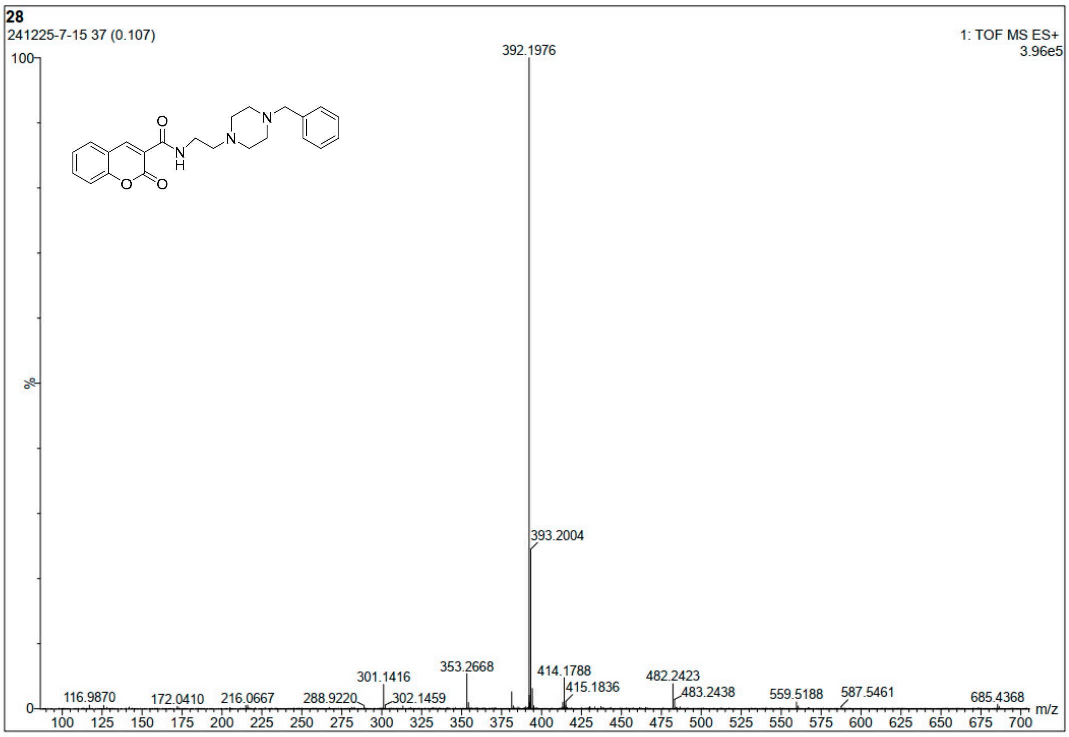

Figure S31. HRMS ([M+H]$^+$) spectrum of compound **6**.

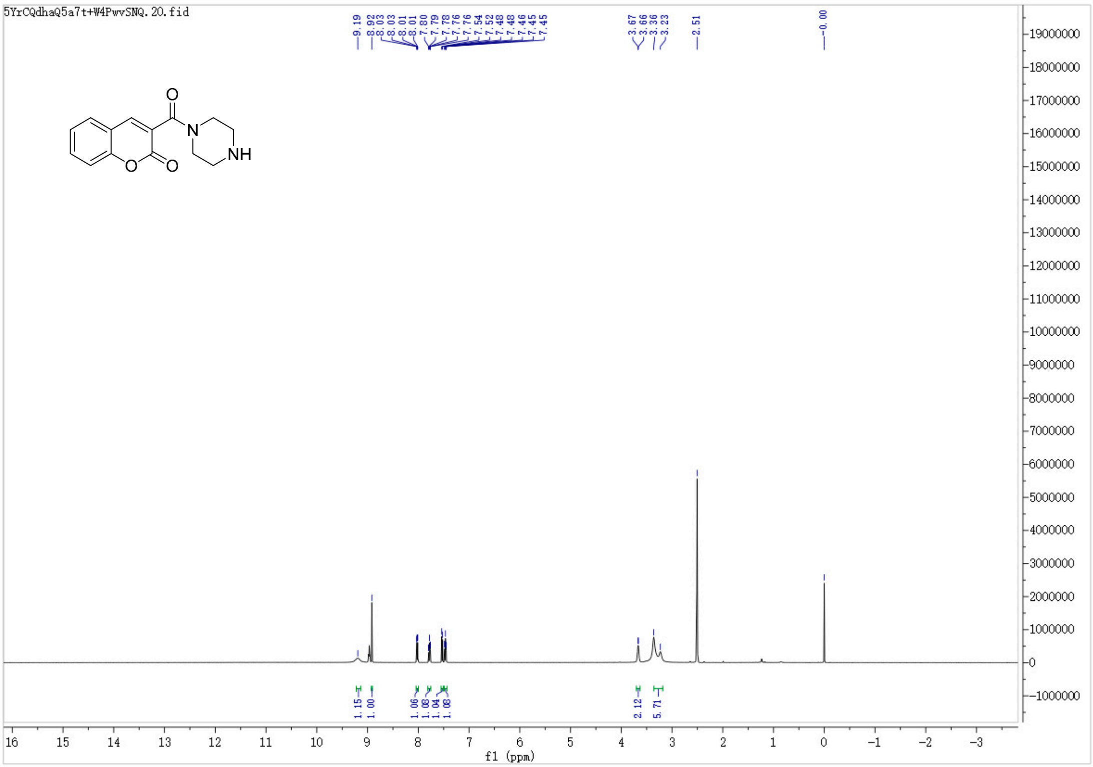

Figure S32. ^{1}H-NMR (DMSO$_{d6}$, 500 MHz, ppm) spectrum of compound **8**.

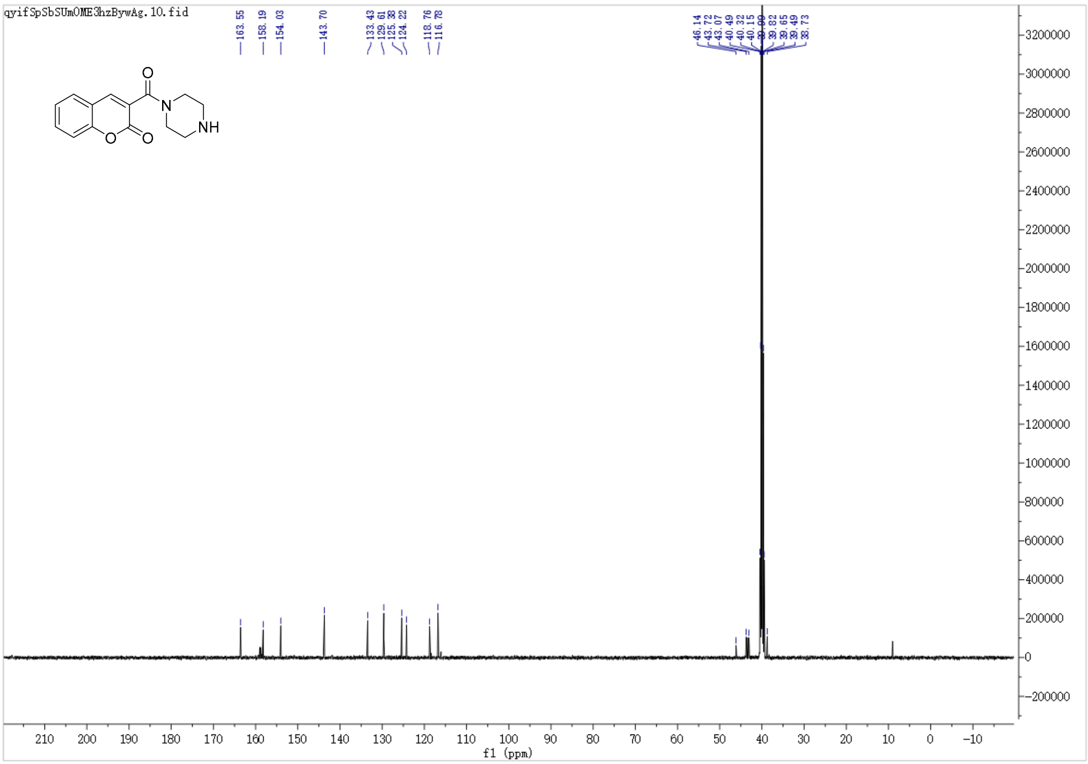

Figure S33. ^{13}C-NMR (DMSO$_{d6}$, 126 MHz, ppm) spectrum of compound **8**.

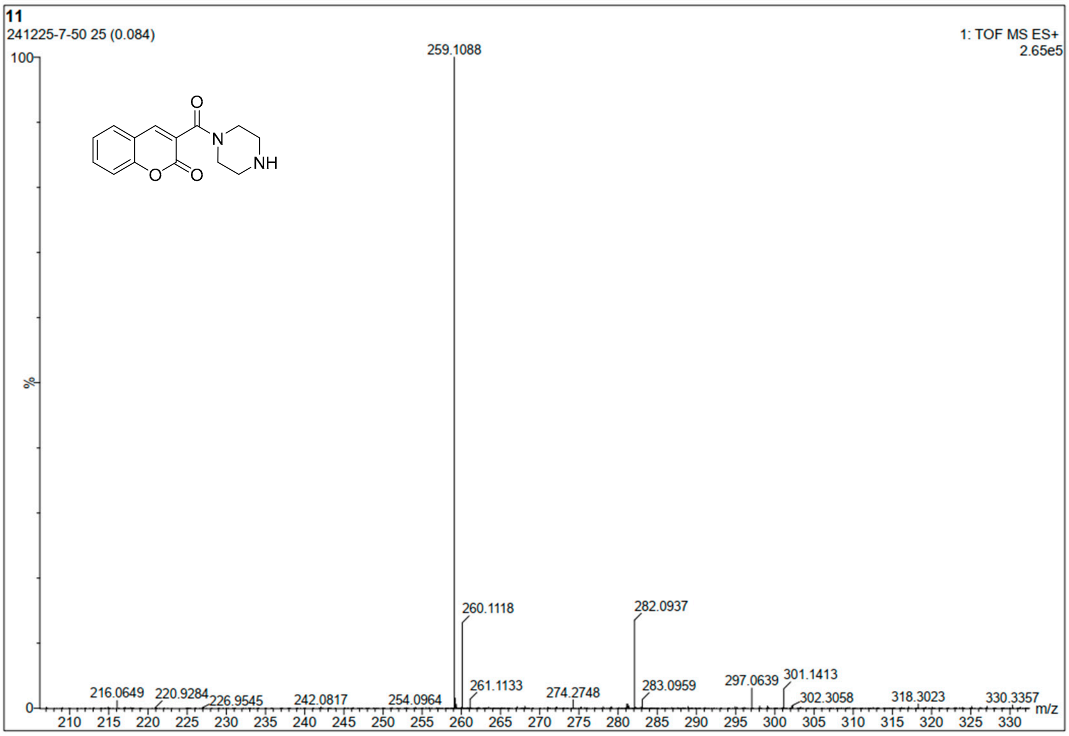

Figure S34. HRMS ([M+H]$^{+}$) spectrum of compound **8**.

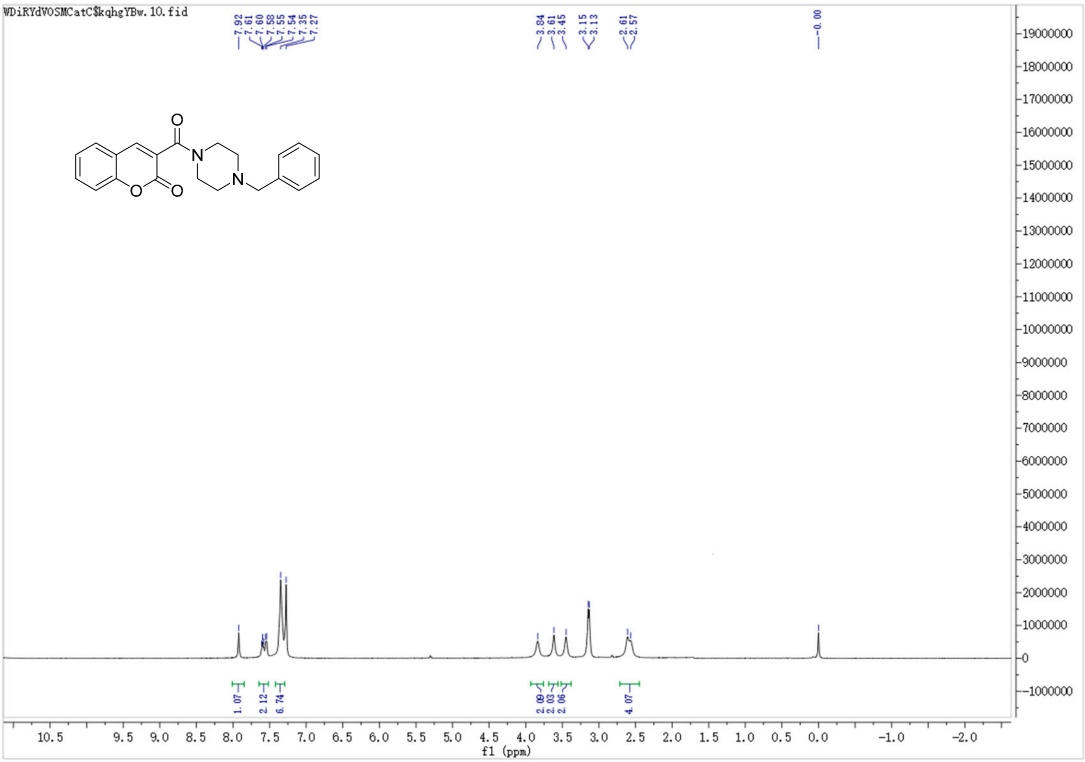

Figure S35. ^{1}H-NMR ($CDCl_3$, 500 MHz, ppm) spectrum of compound **9**.

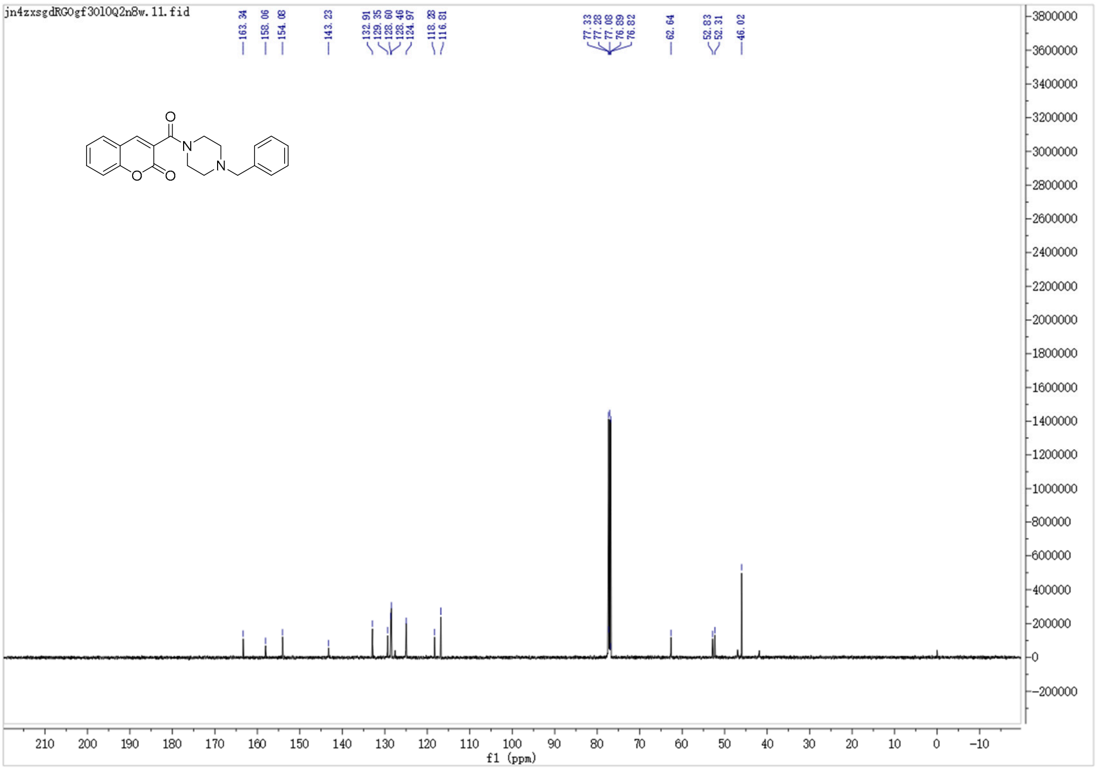

Figure S36. ^{13}C-NMR ($CDCl_3$, 126 MHz, ppm) spectrum of compound **9**.

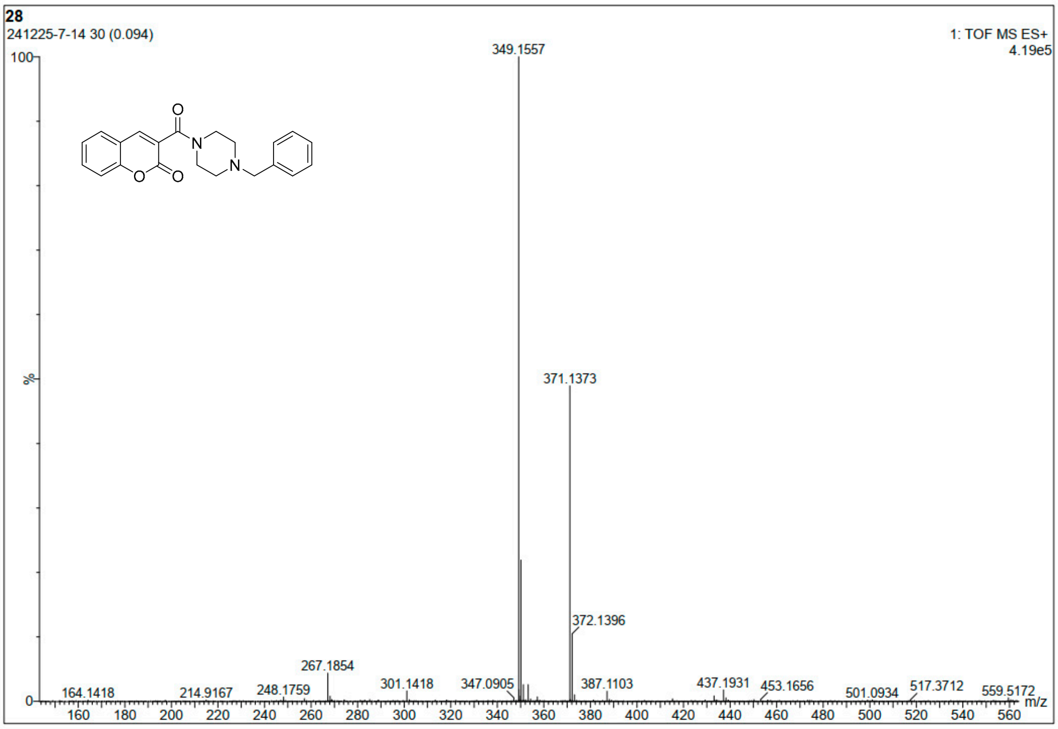

Figure S37. HRMS ($[M+H]^+$) spectrum of compound **9**.

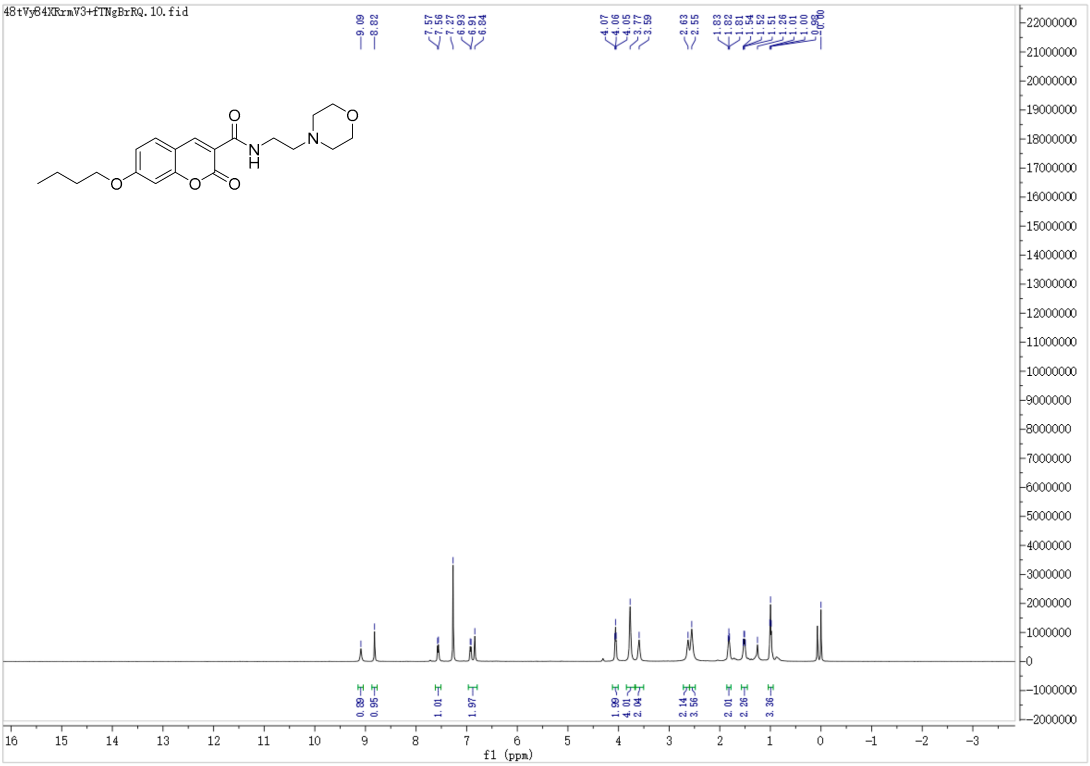

Figure S38. ^{1}H-NMR ($CDCl_3$, 500 MHz, ppm) spectrum of compound **14a**.

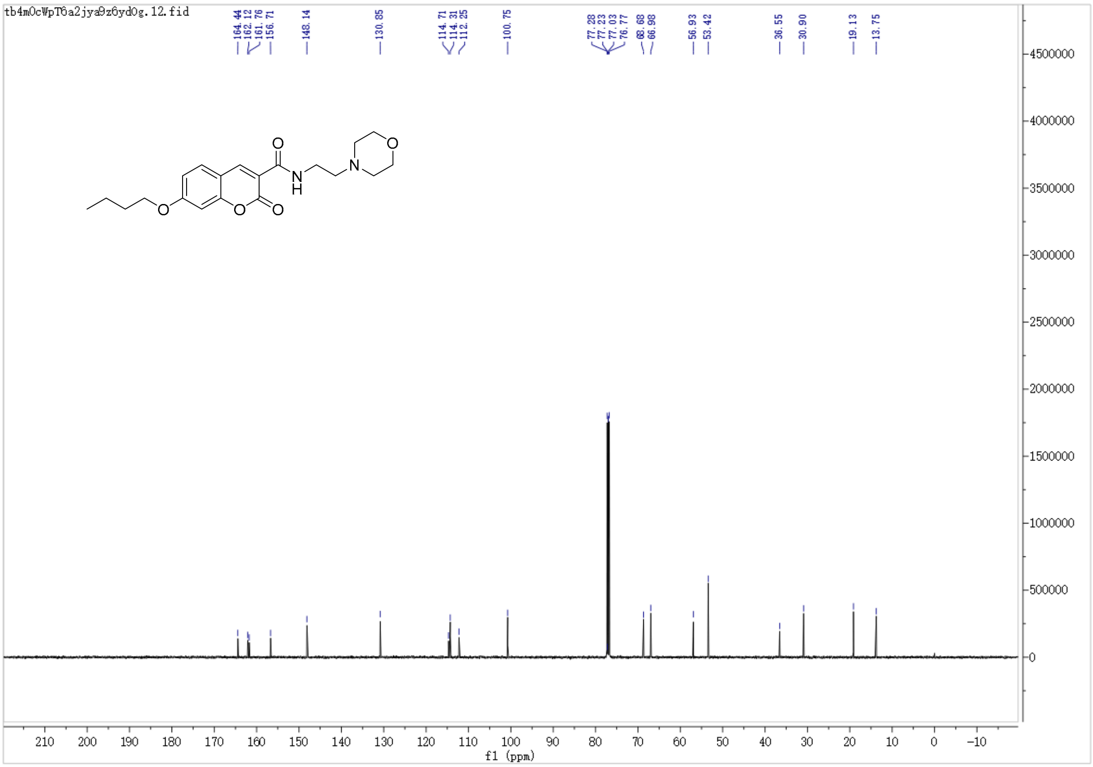

Figure S39. ^{13}C-NMR ($CDCl_3$, 126 MHz, ppm) spectrum of compound **14a**.

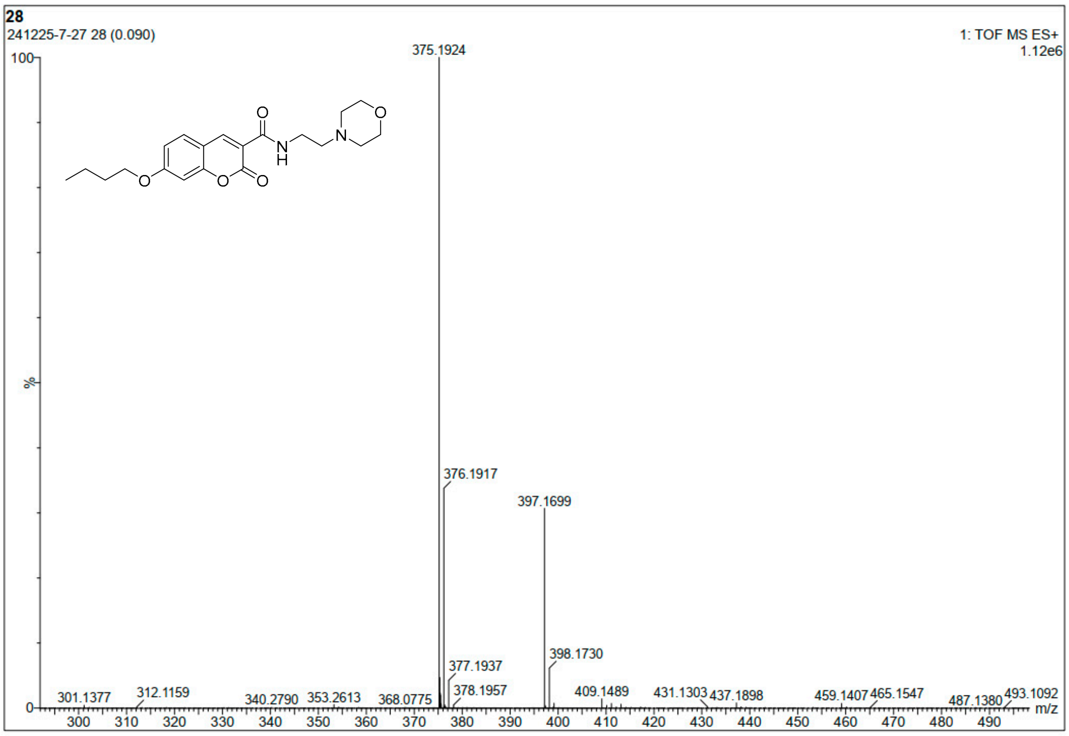

Figure S40. HRMS ([M+H]$^+$) spectrum of compound **14a**.

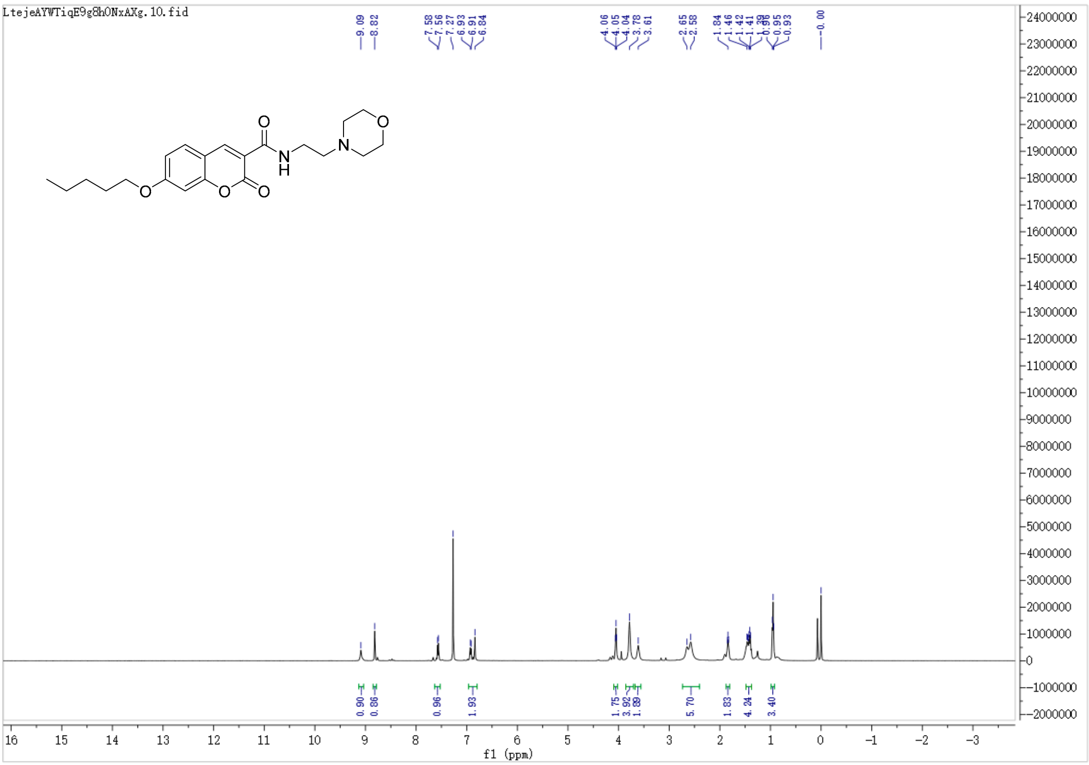

Figure S41. ^{1}H-NMR ($CDCl_3$, 500 MHz, ppm) spectrum of comound **14b**.

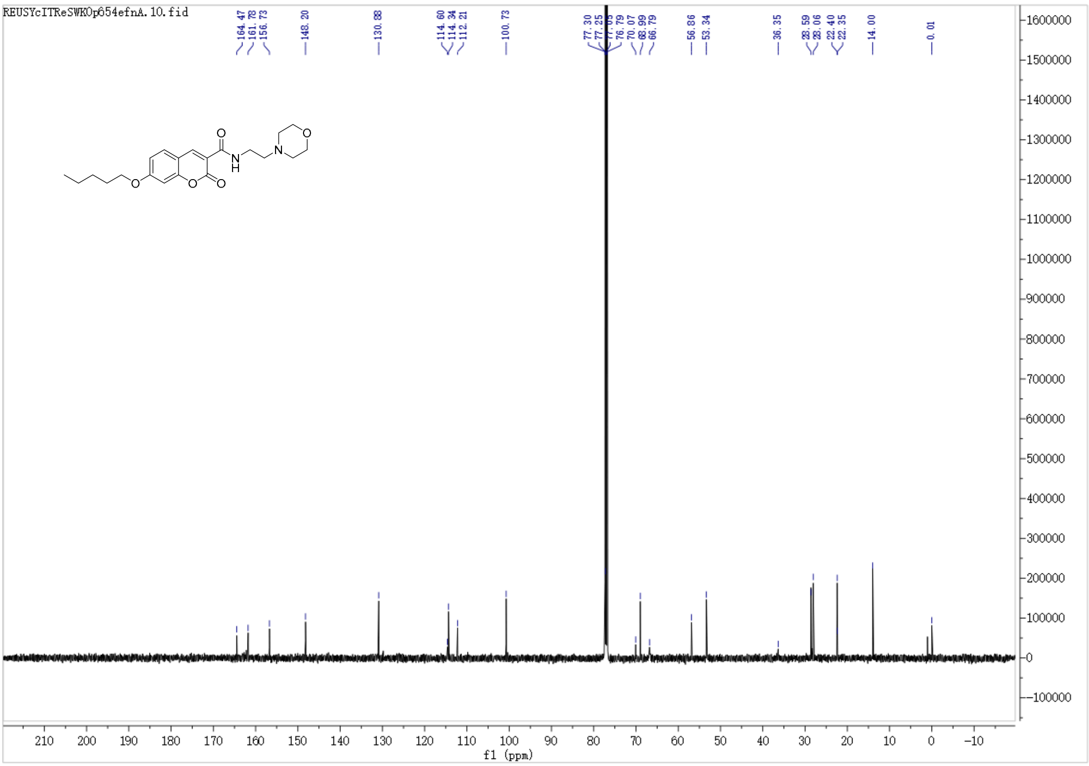

Figure S42. ^{13}C-NMR ($CDCl_3$, 126 MHz, ppm)spectrum of compound **14b**.

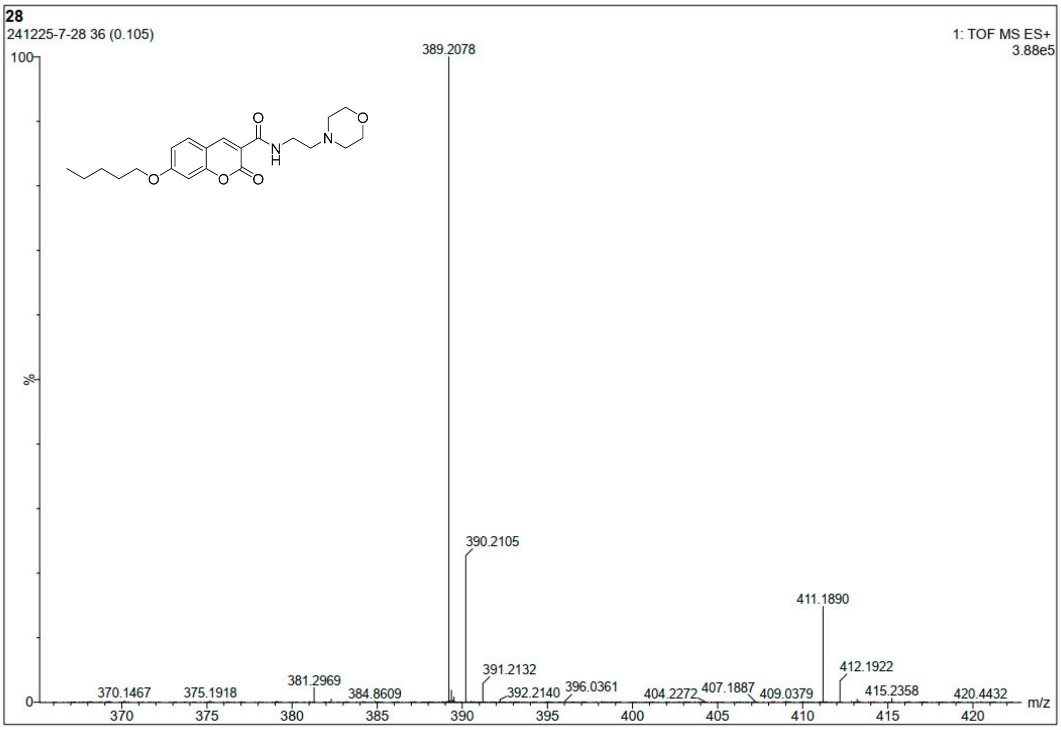

Figure S43. HRMS ([M+H]$^+$) spectrum of compound **14b**.

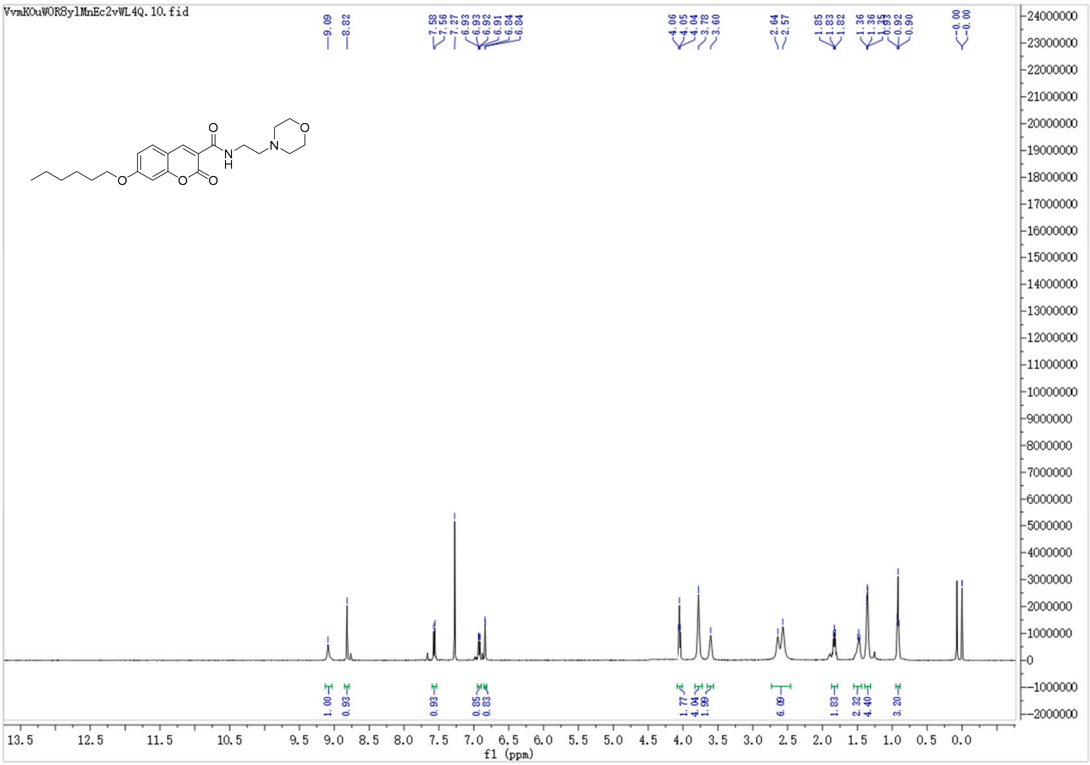

Figure S44. ^{1}H-NMR ($CDCl_3$, 500 MHz, ppm) spectrum of compound **14c**.

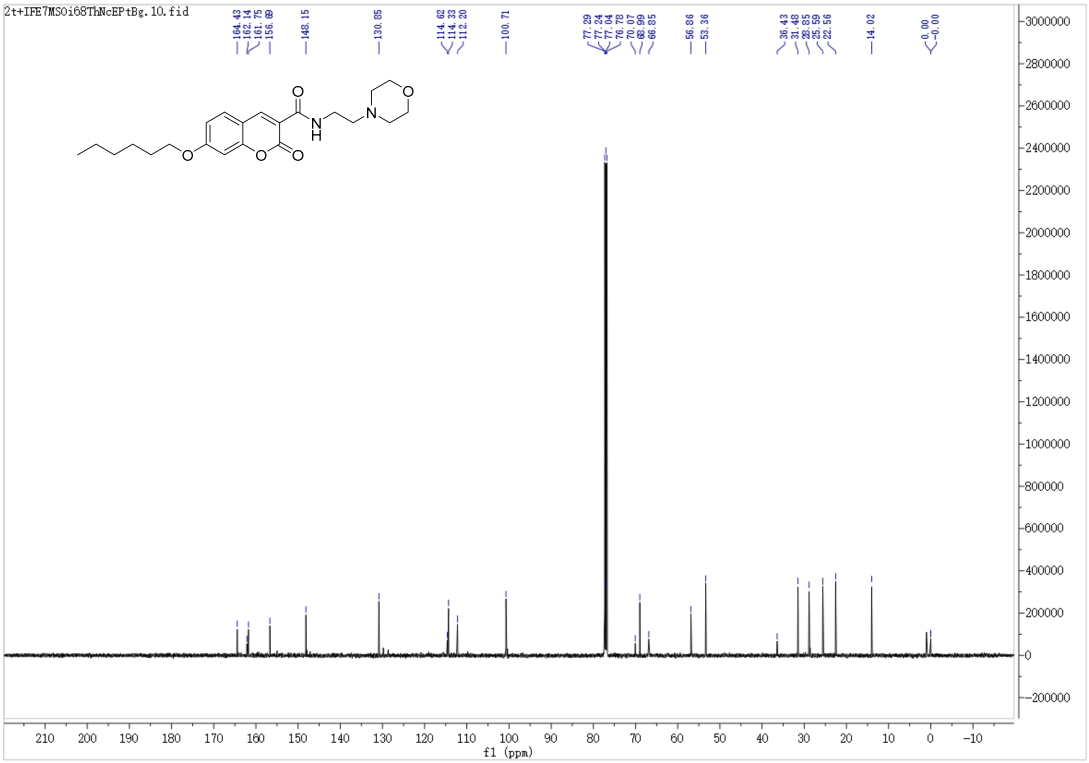

Figure S45. ^{13}C-NMR ($CDCl_3$, 126 MHz, ppm) spectrum of compound **14c**.

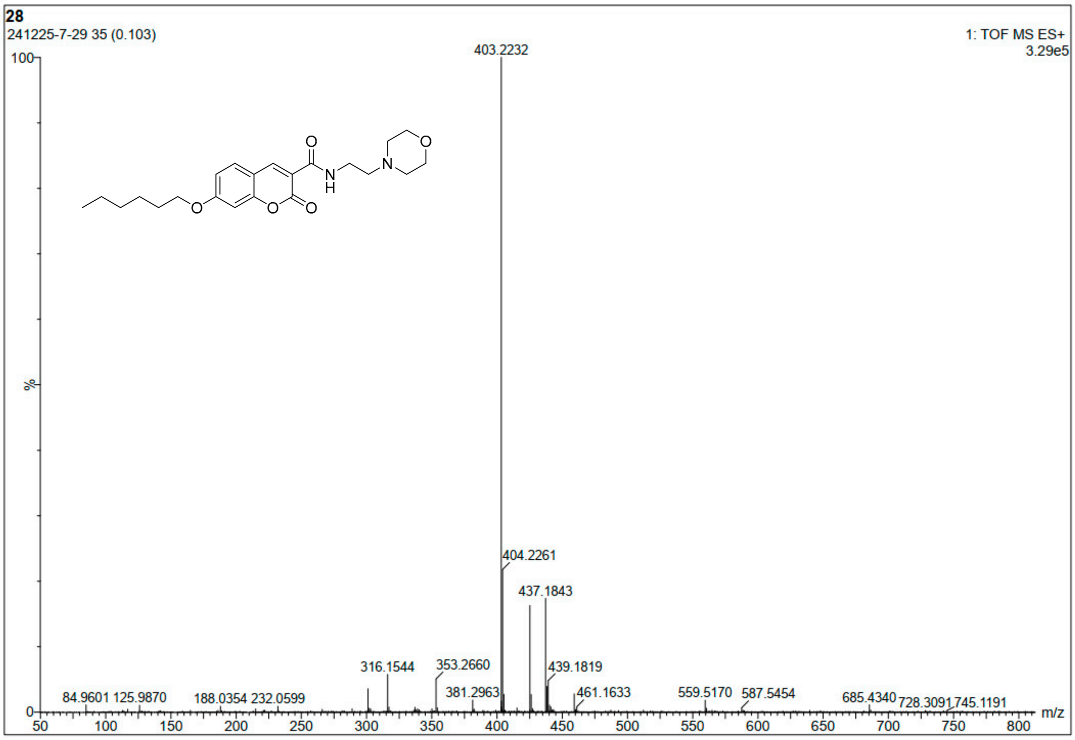

Figure S46. HRMS ([M+H]$^+$) spectrum of compound **14c**.

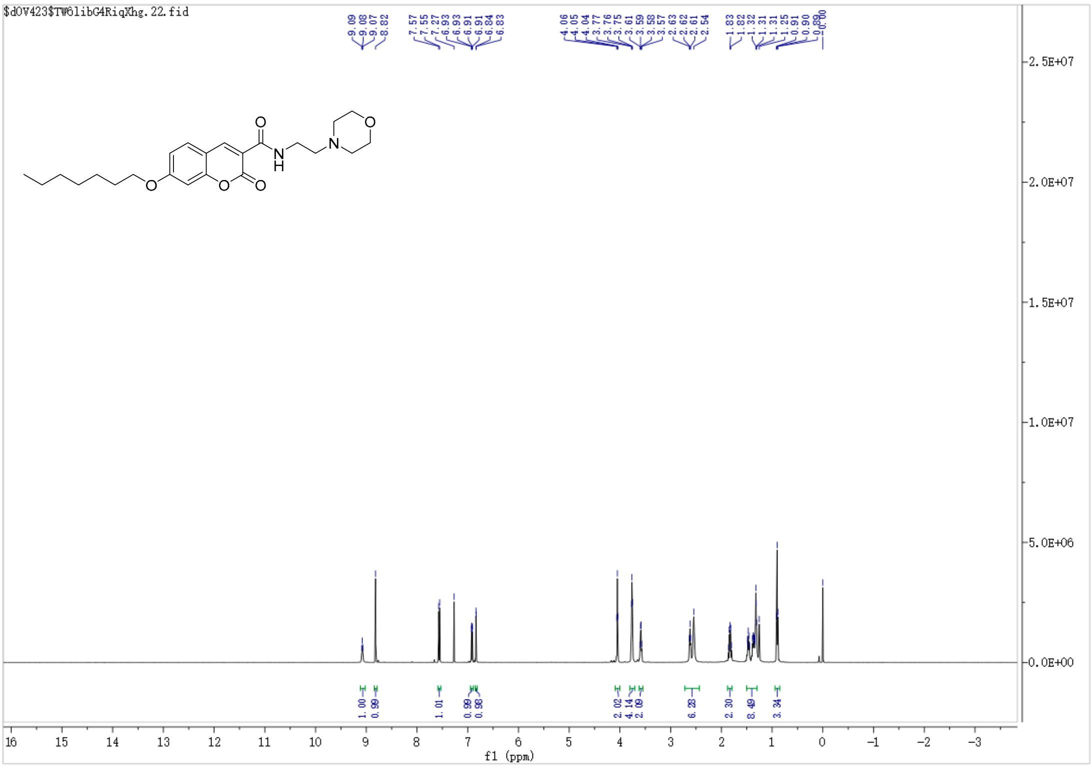

Figure S47. ^{1}H-NMR (CDCl$_3$, 500 MHz, ppm) spectrum of compound **14d**.

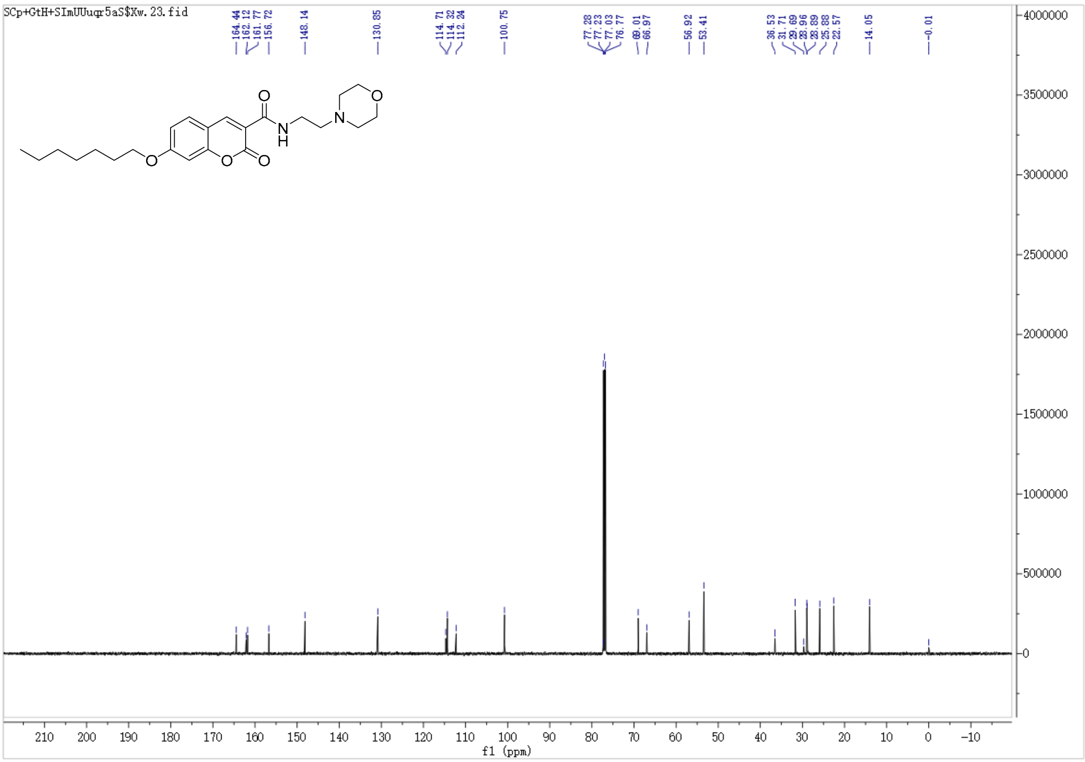

Figure S48. ^{13}C-NMR ($CDCl_3$, 126 MHz, ppm) spectrum of compound **14d**.

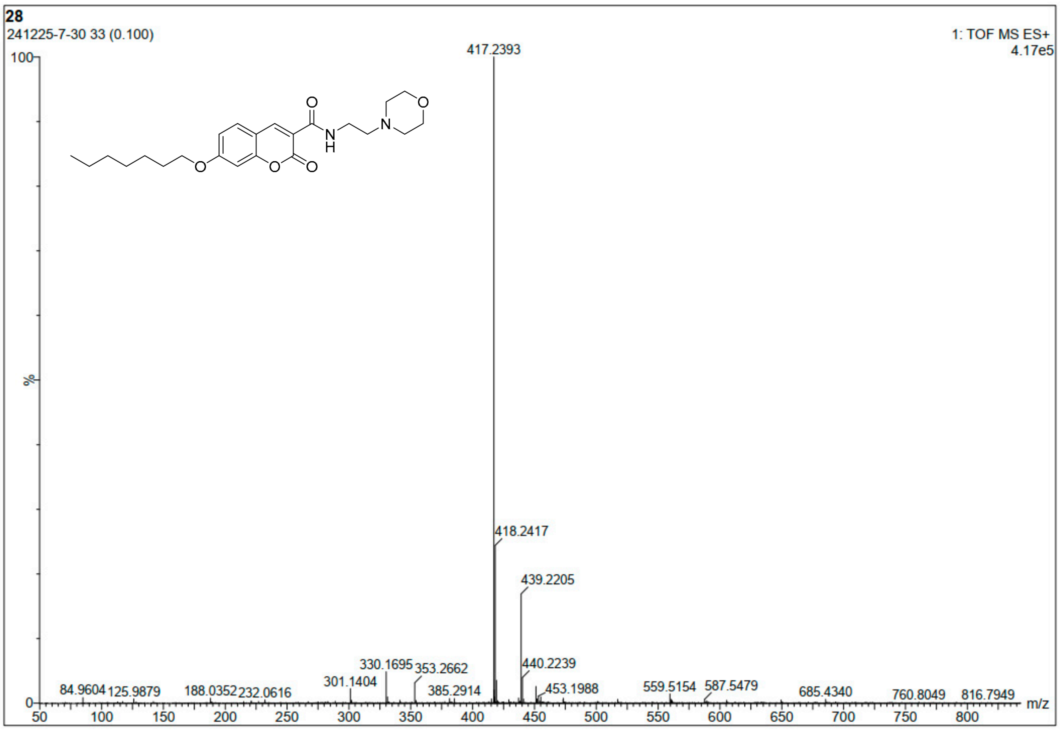

Figure S49. HRMS ($[M+H]^+$) spectrum of compound **14d**.

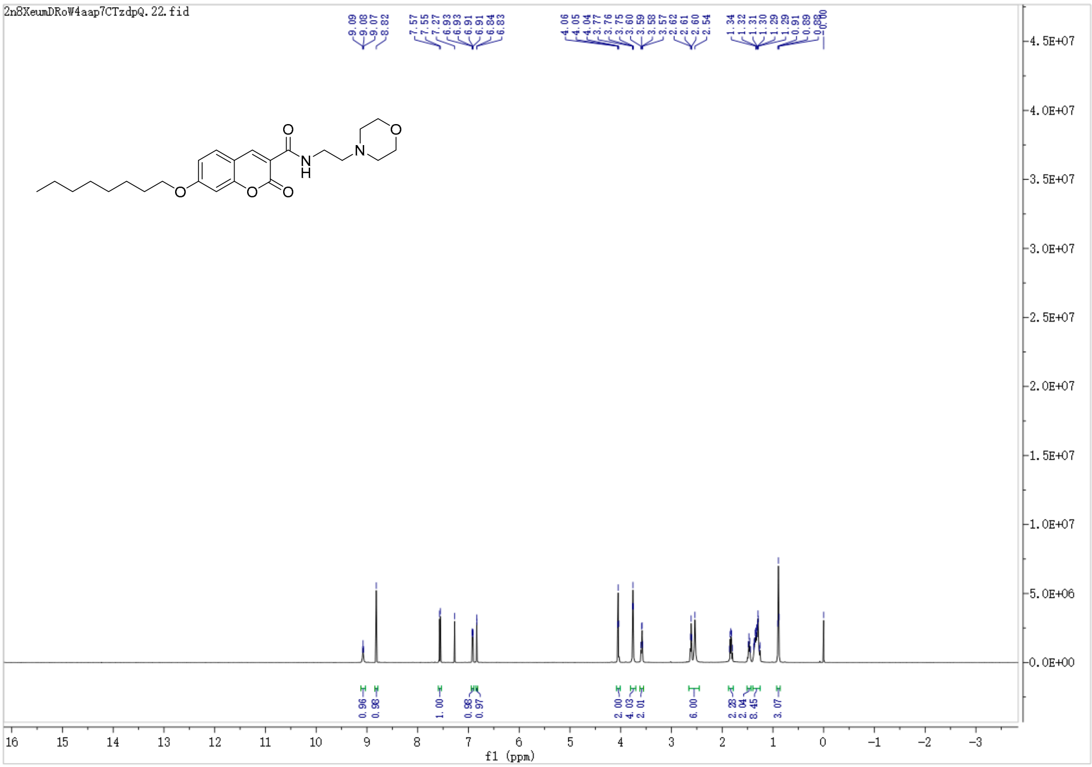

Figure S50. ^{1}H-NMR ($CDCl_3$, 500 MHz, ppm) spectrum of compound **14e**.

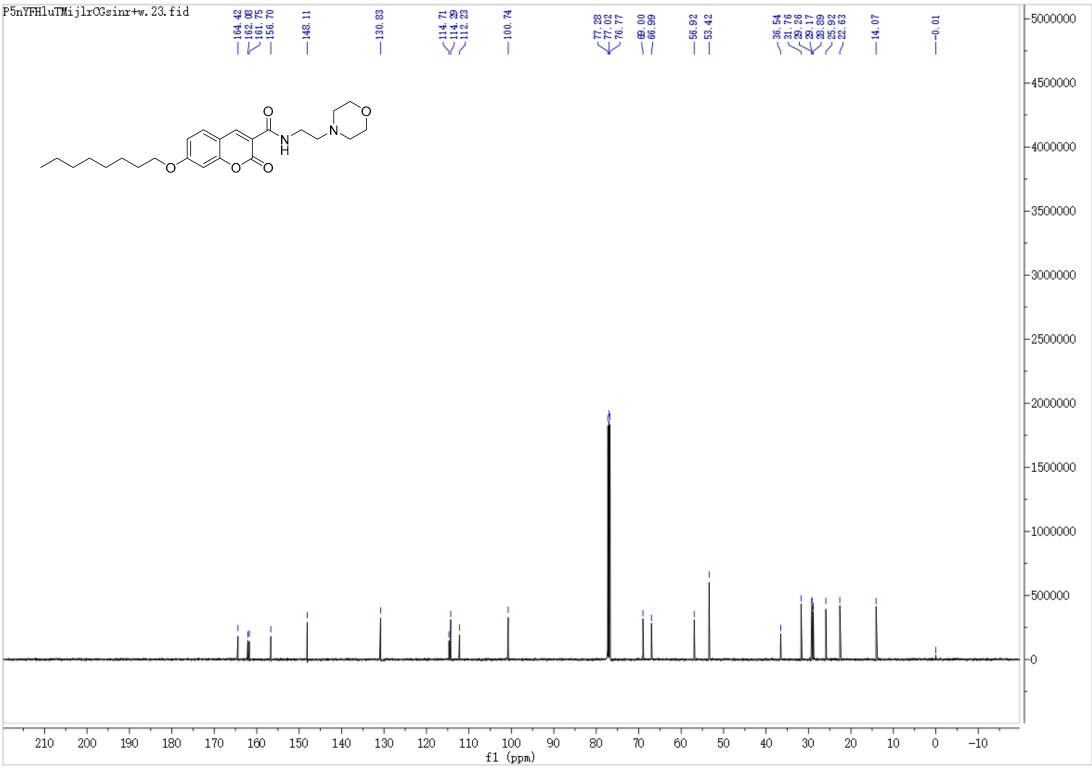

Figure S51. ^{13}C-NMR ($CDCl_3$, 126 MHz, ppm) spectrum of compound **14e**.

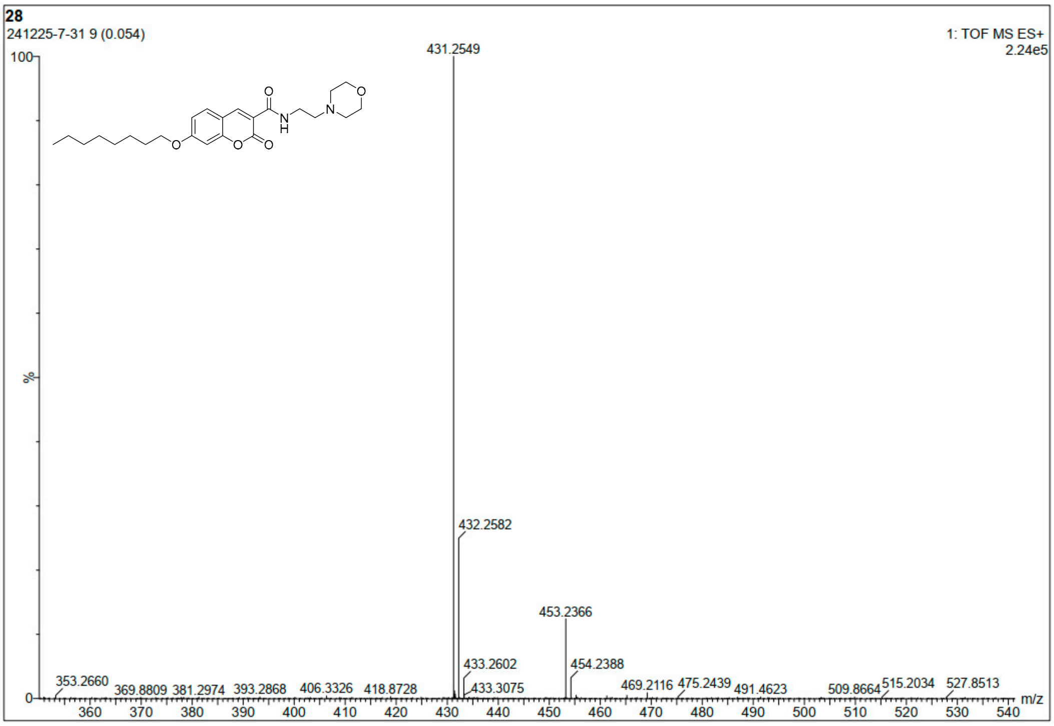

Figure S52. HRMS ([M+H]$^+$) spectrum of compound **14e**.

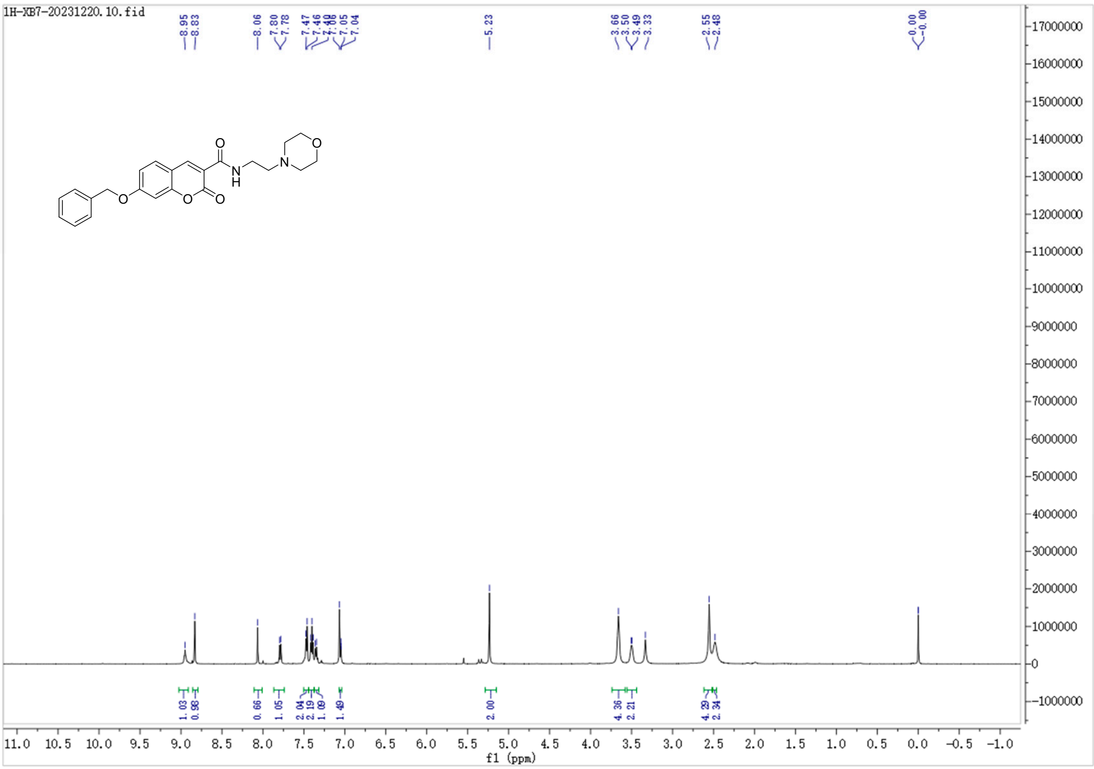

Figure S53. ^{1}H-NMR ($CDCl_3$, 500 MHz, ppm) spectrum of compound **14f**.

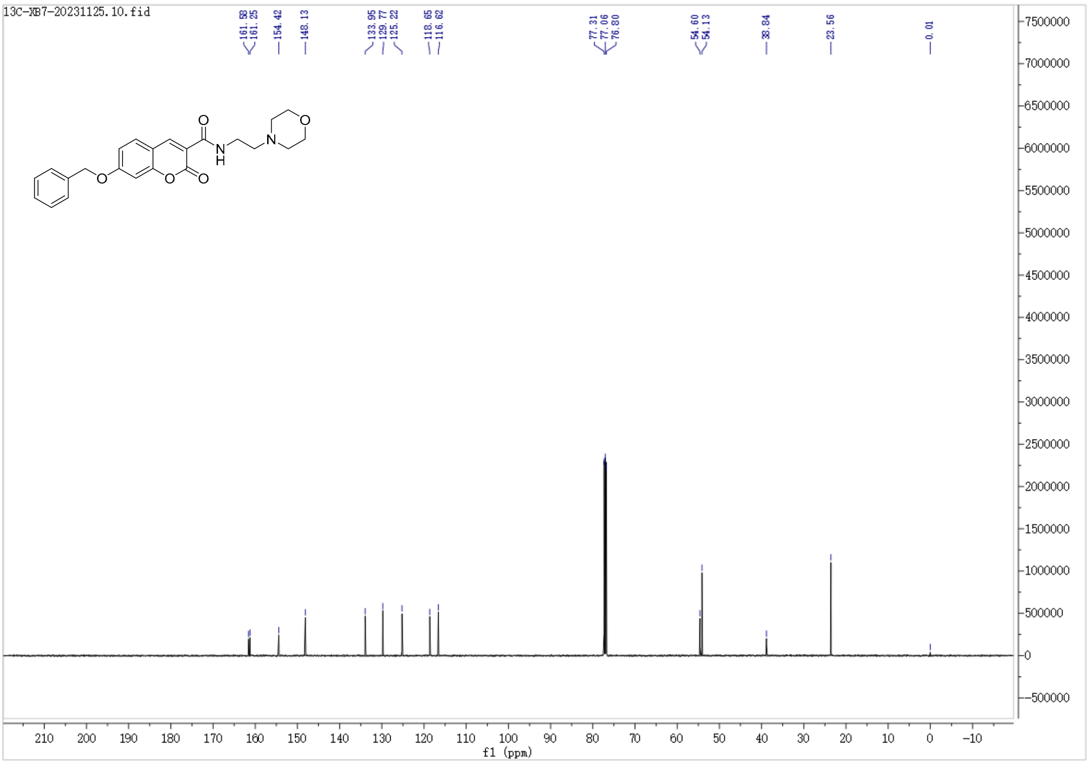

Figure S54. ^{13}C-NMR ($CDCl_3$, 126 MHz, ppm) spectrum of compound **14f**.

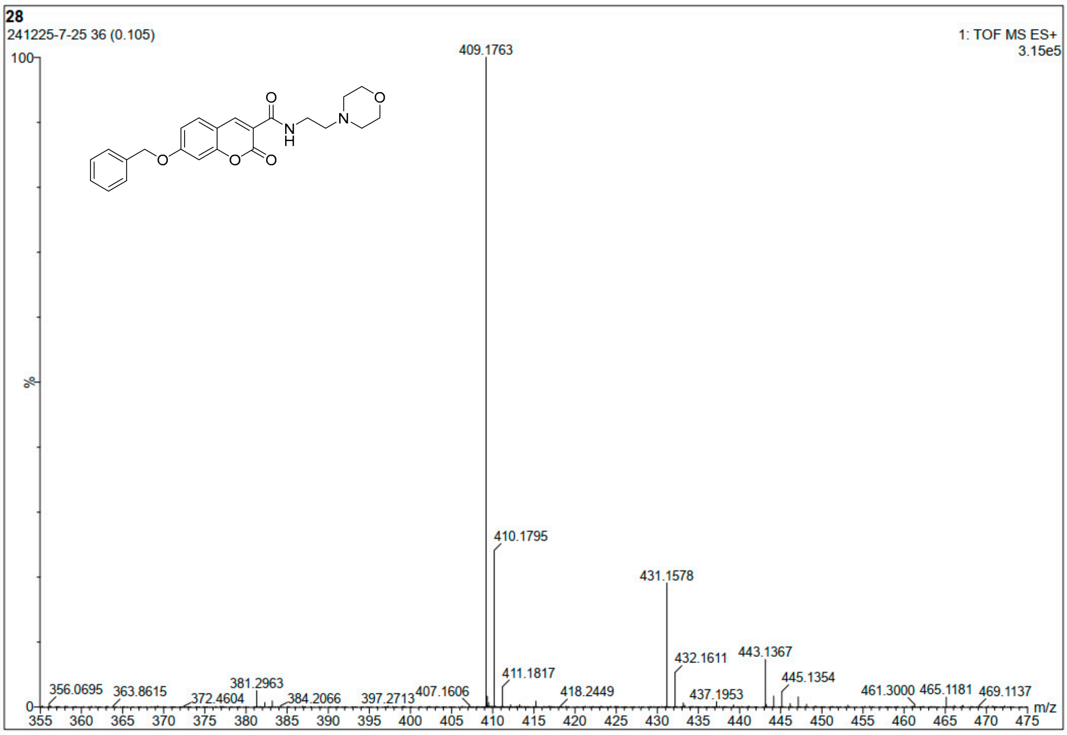

Figure S55. HRMS ($[M+H]^+$) spectrum of compound **14f**.

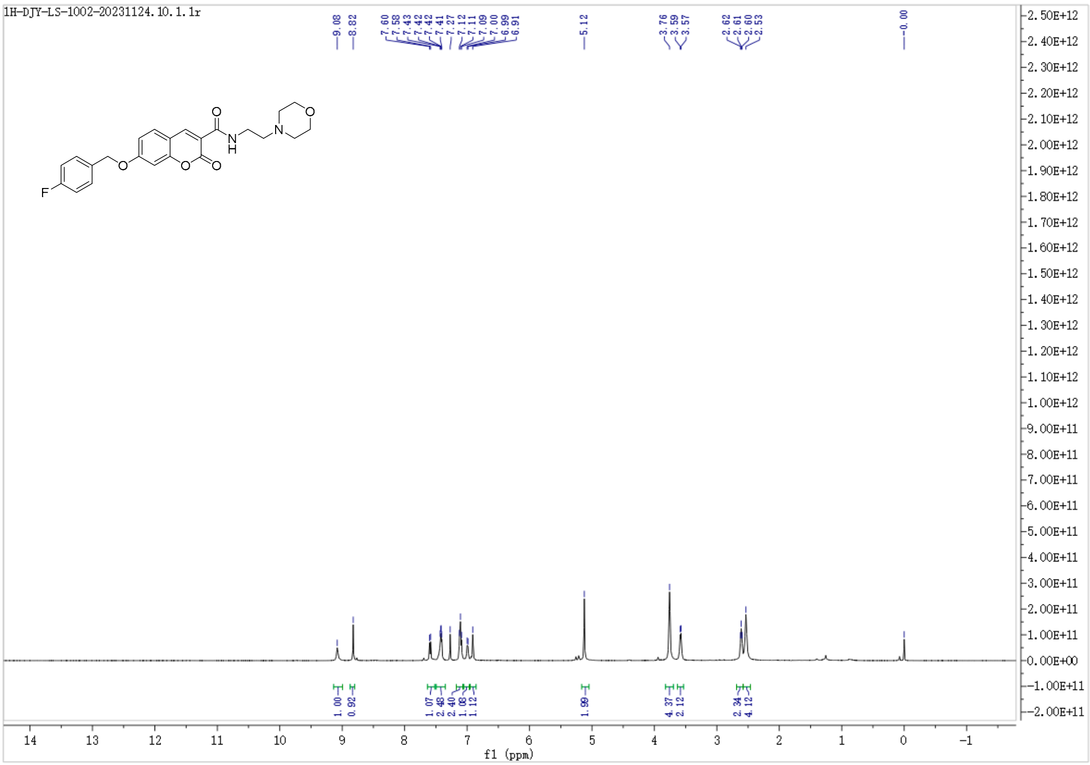

Figure S56. ^{1}H-NMR ($CDCl_3$, 500 MHz, ppm) spectrum of compound **14g**.

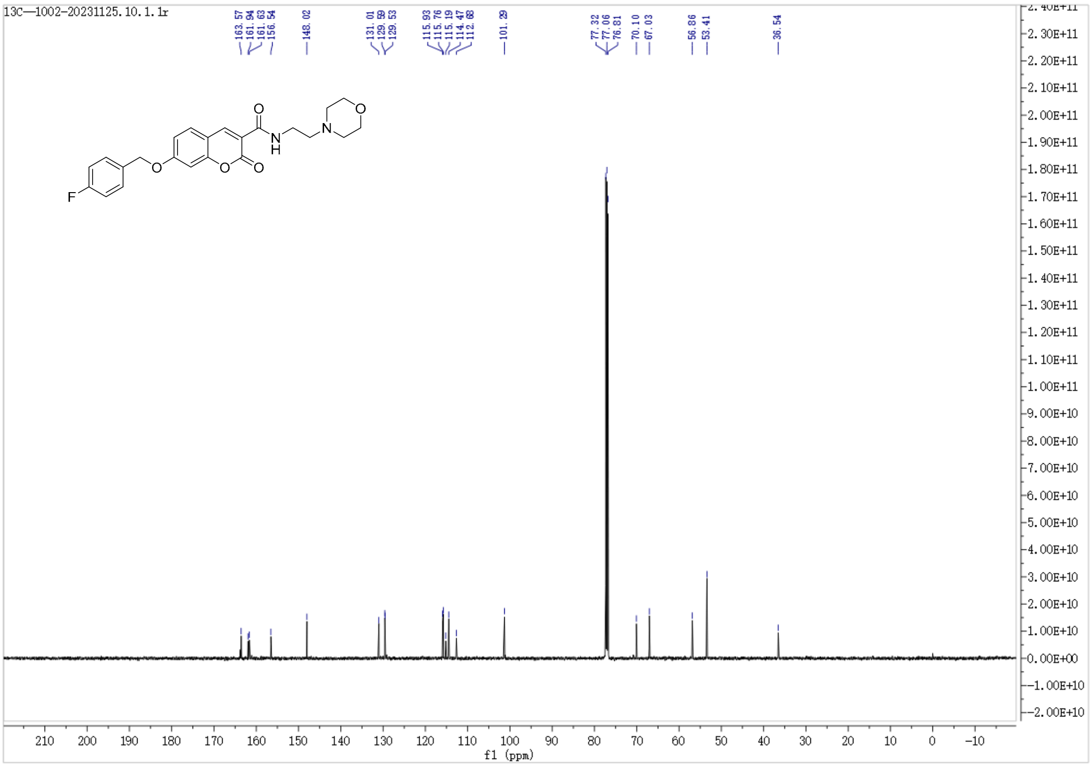

Figure S57. ^{13}C-NMR ($CDCl_3$, 126 MHz, ppm) spectrum of compound **14g**.

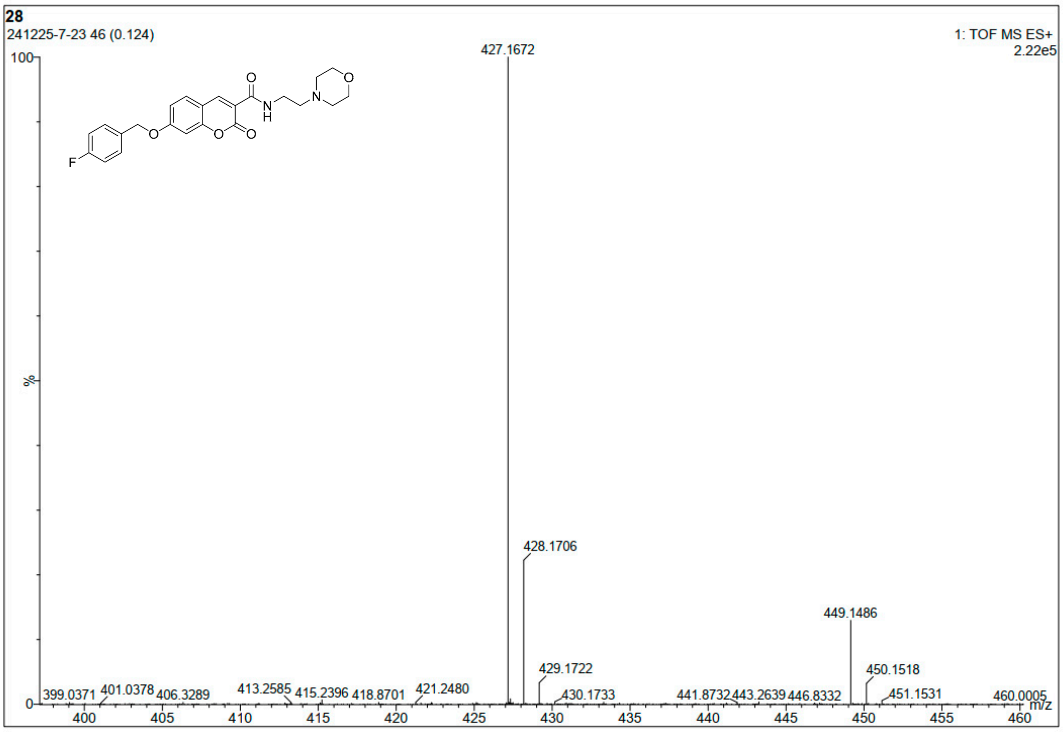

Figure S58. HRMS ([M+H]$^+$) spectrum of compound **14g**.

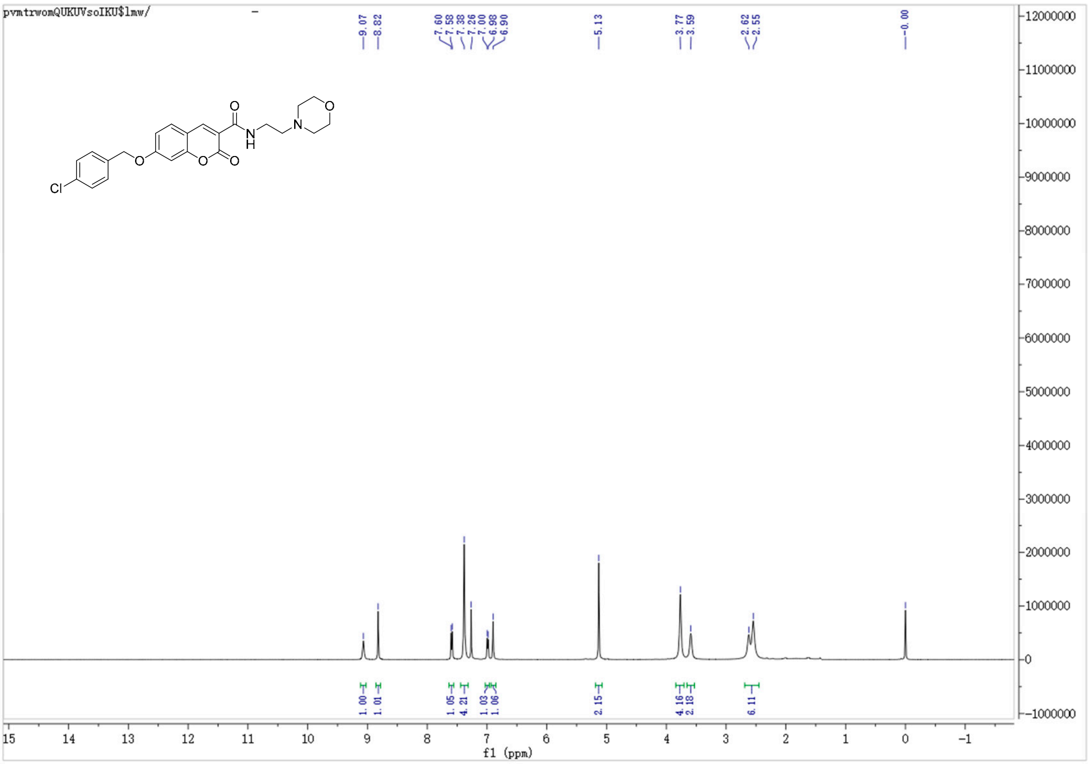

Figure S59. ^{1}H-NMR (CDCl$_3$, 500 MHz, ppm) spectrum of compound **14h**.

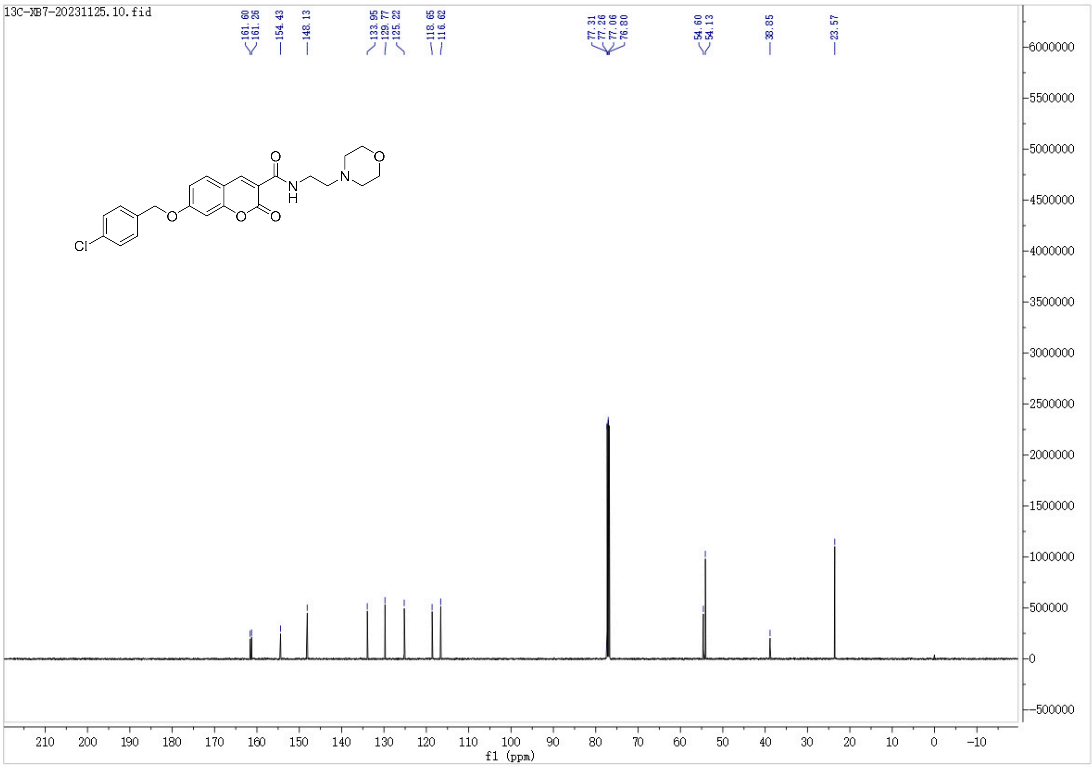

Figure S60. ^{13}C-NMR ($CDCl_3$, 126 MHz, ppm) spectrum of compound **14h**.

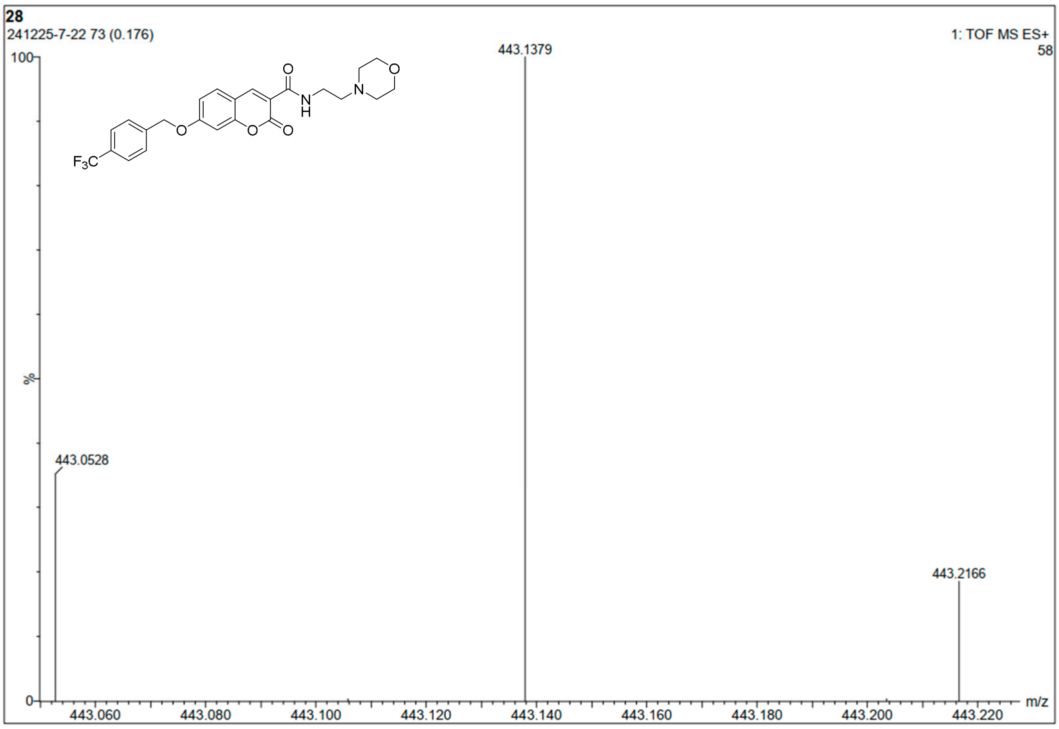

Figure S61. HRMS ($[M+H]^+$) spectrum of compound **14h**.

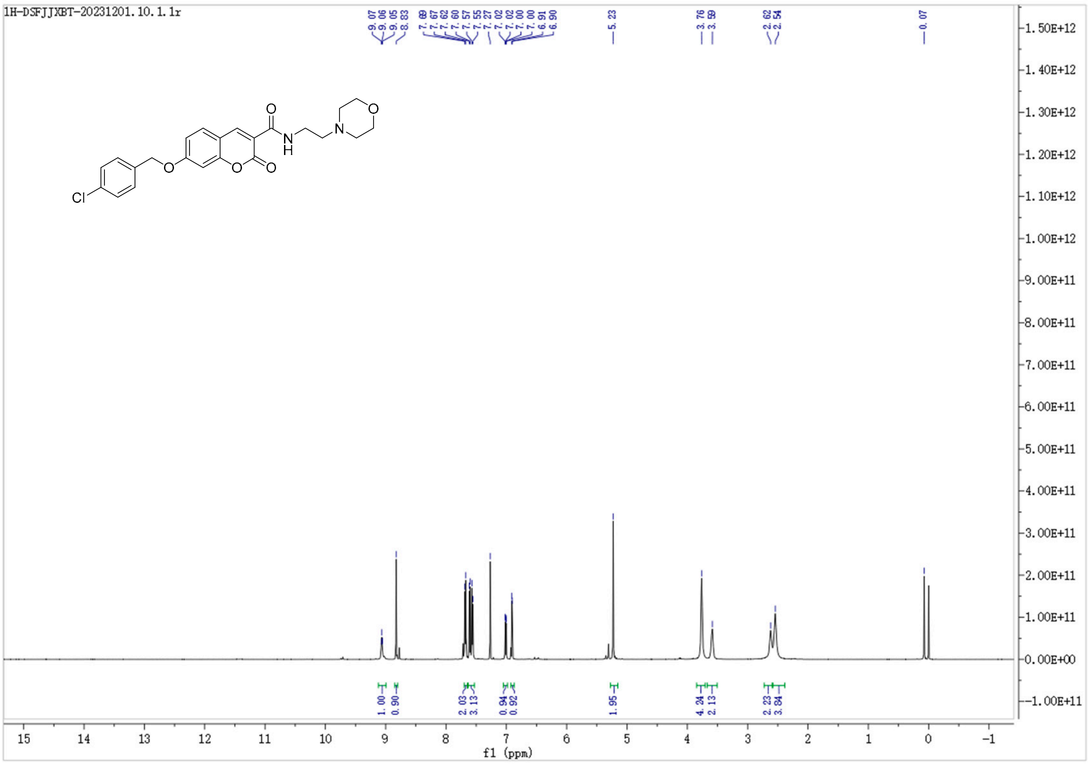

Figure S62. ^{1}H-NMR ($CDCl_3$, 500 MHz, ppm) spectrum of compound **14i**.

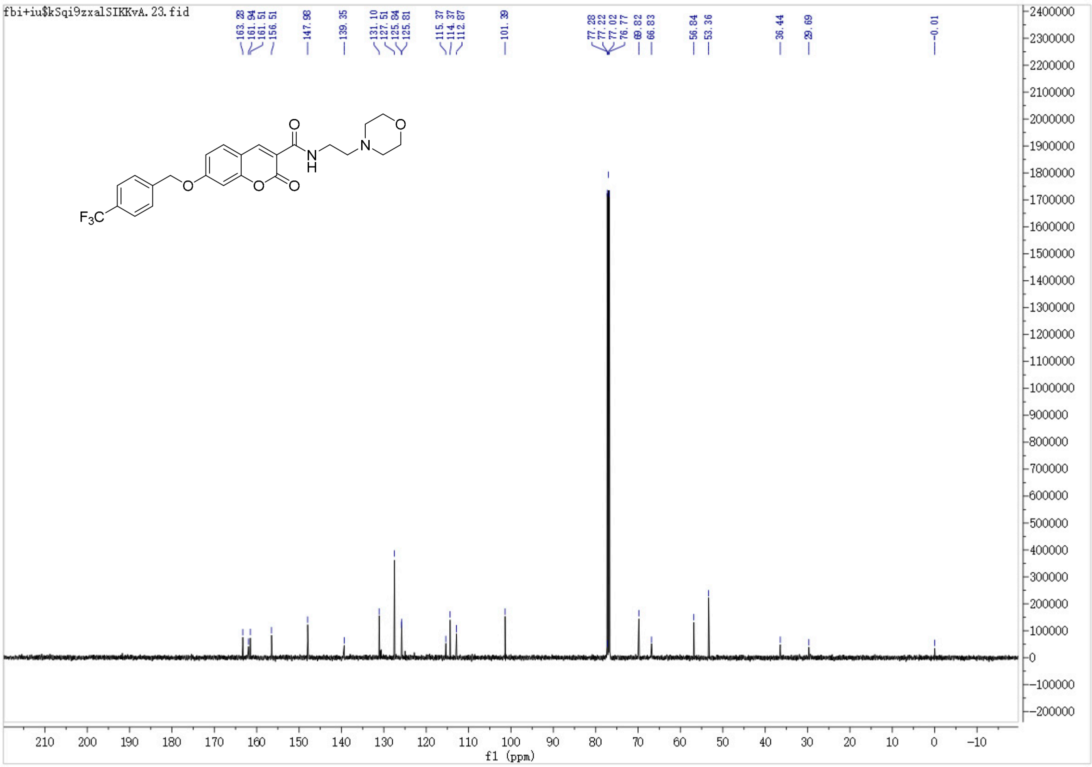

Figure S63. ^{13}C-NMR ($CDCl_3$, 126 MHz, ppm) spectrum of compound **14i**.

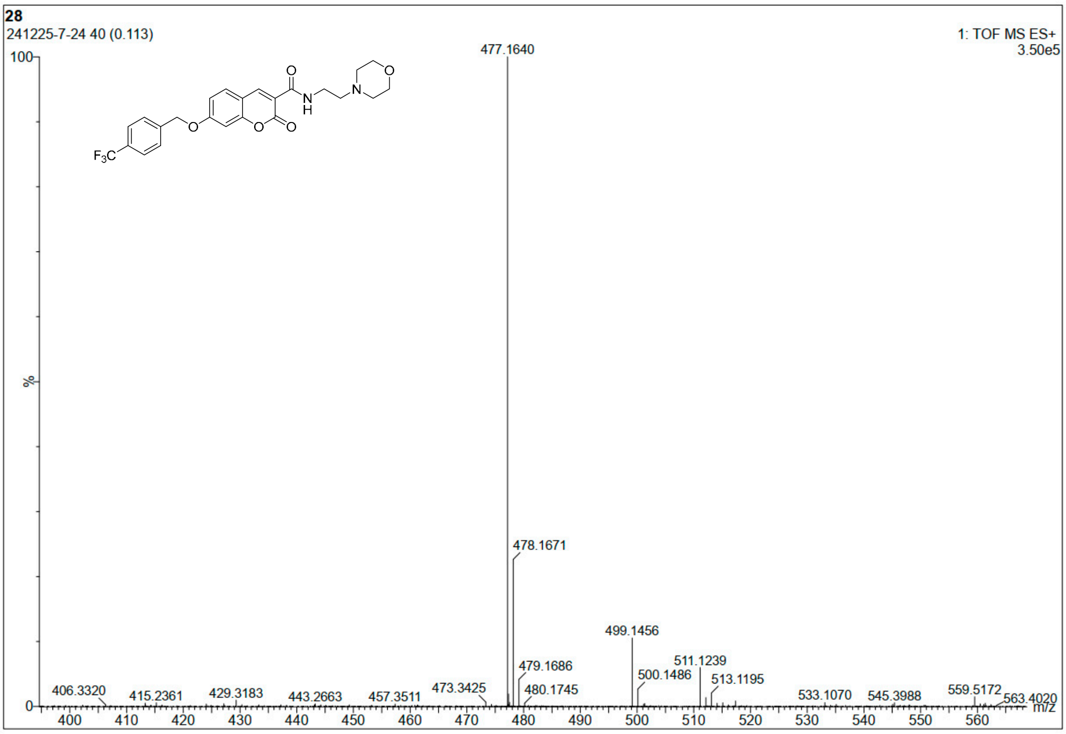

Figure S64. HRMS ($[M+H]^+$) spectrum of compound **14i**.

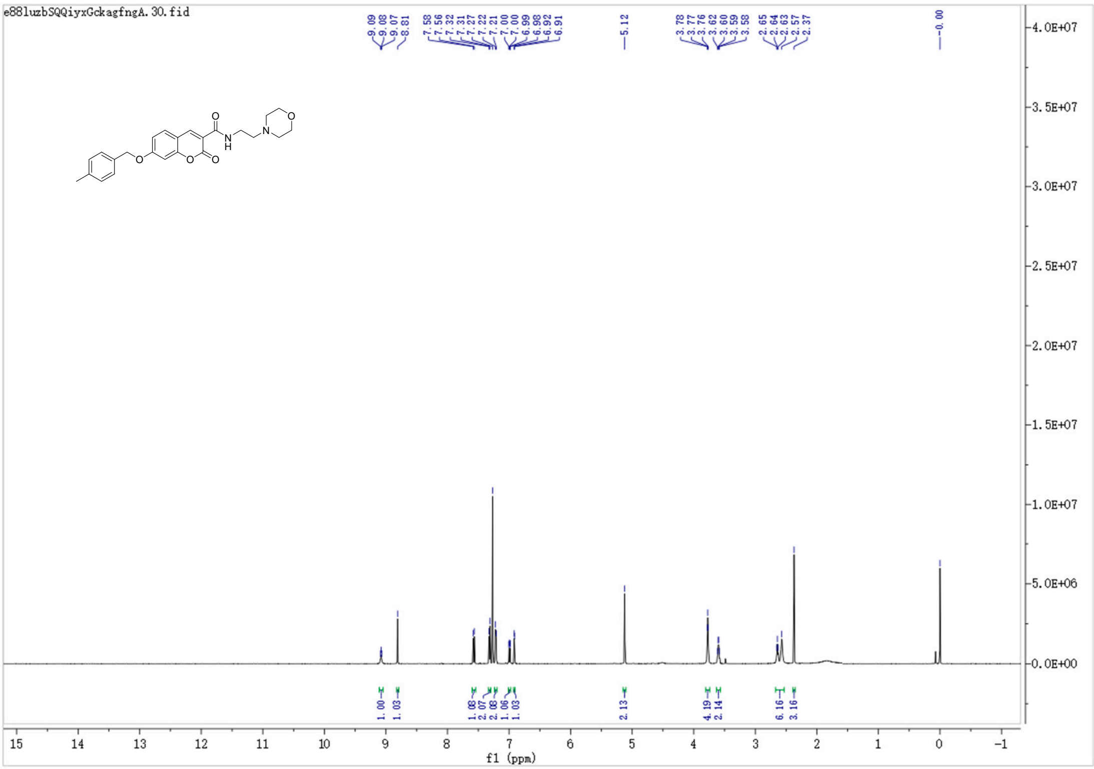

Figure S65. ^{1}H-NMR ($CDCl_3$, 500 MHz, ppm) spectrum of compound **14j**.

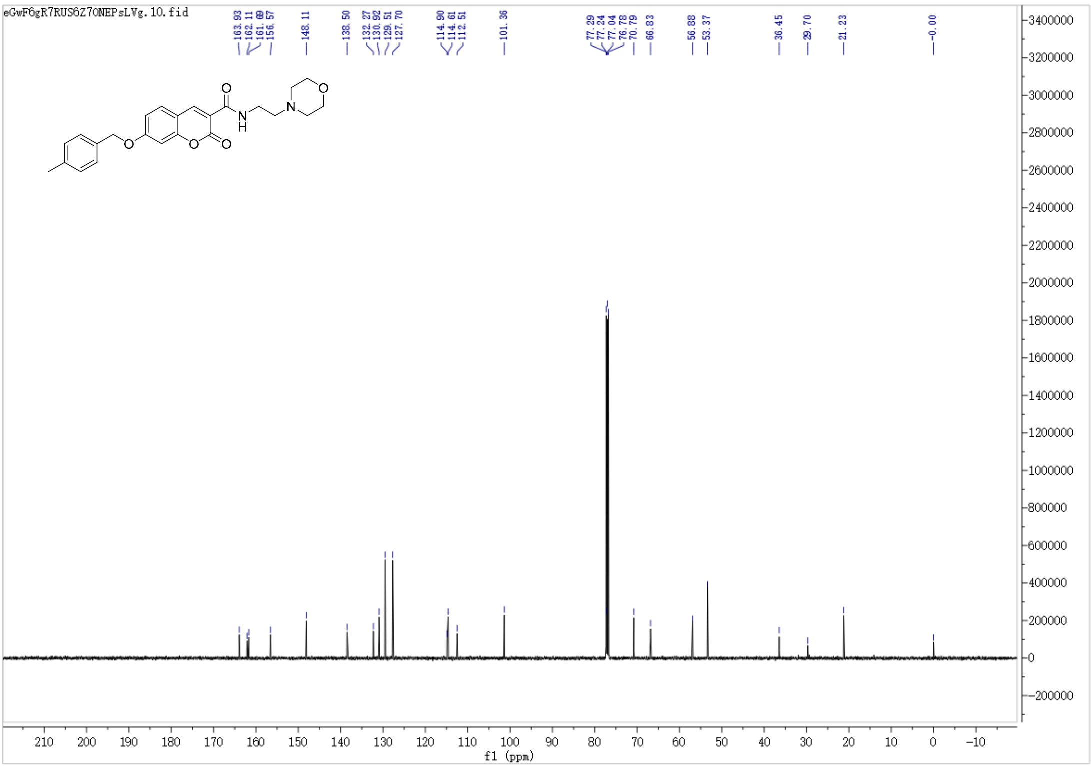

Figure S66. ^{13}C-NMR ($CDCl_3$, 126 MHz, ppm) spectrum of compound **14j**.

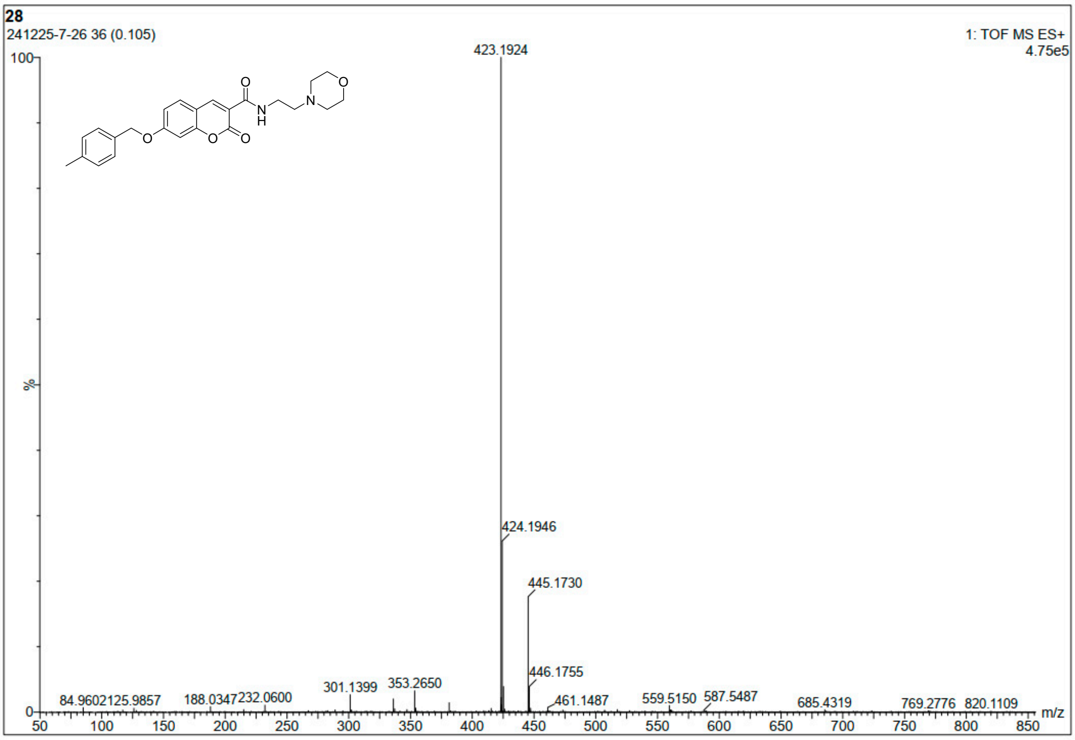

Figure S67. HRMS ($[M+H]^+$) spectrum of compound **14j**.

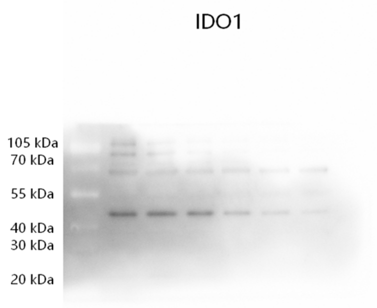

Figure S68. Expression levels of IDO1.

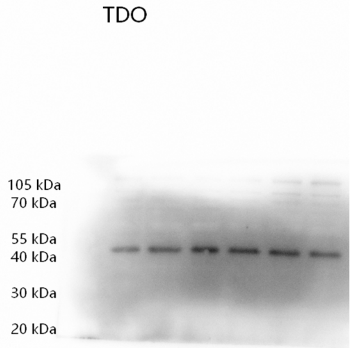

Figure S69. Expression levels of TDO.

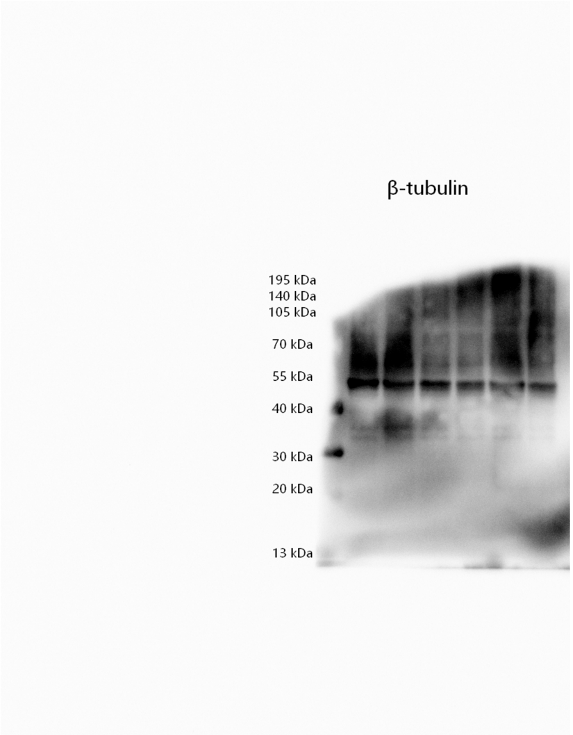

Figure S70. Expression levels of β-tubulin.

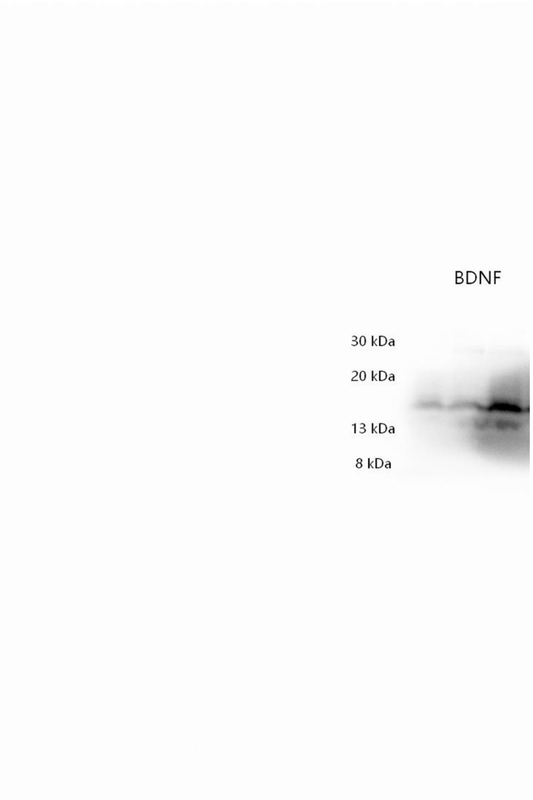

Figure S71. Expression levels of BDNF.

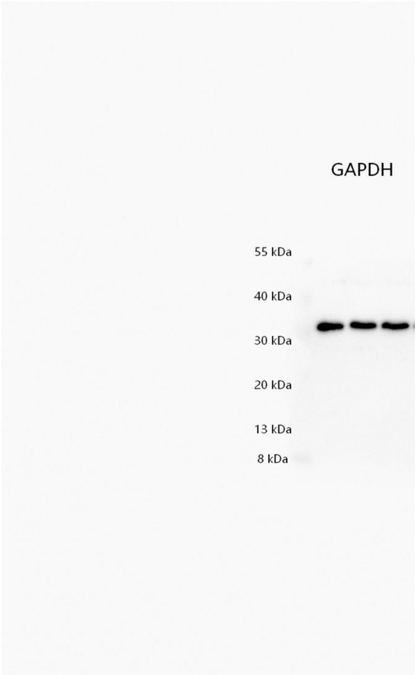

Figure S72. Expression levels of GAPDH.

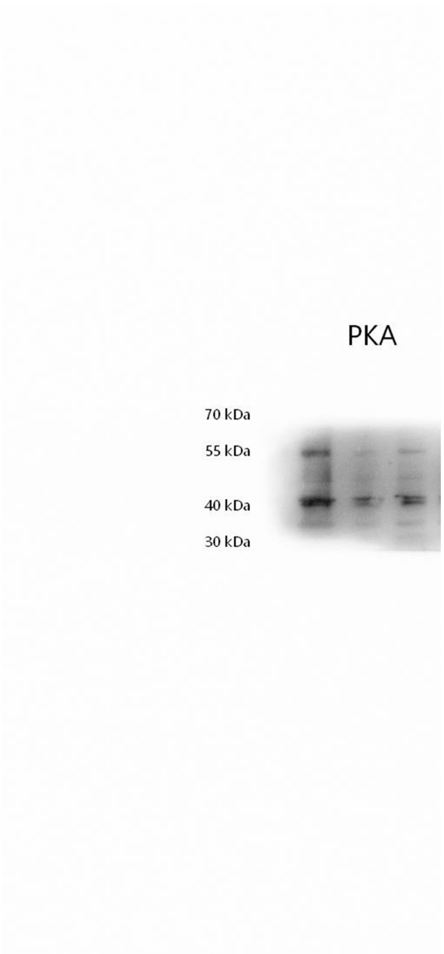

Figure S73. Expression levels of PKA.
